# Measures of Homozygosity and Relationship to Genetic Diversity in the Bearded Collie Breed

**DOI:** 10.3390/genes16040378

**Published:** 2025-03-27

**Authors:** Janelle M. Belanger, Liza C. Gershony, Jerold S. Bell, Marjo K. Hytönen, Hannes Lohi, Kerstin Lindblad-Toh, Katarina Tengvall, Elsa Sell, Thomas R. Famula, Anita M. Oberbauer

**Affiliations:** 1 Department of Animal Science, University of California, Davis, CA 95616, USA; jmbelanger@ucdavis.edu (J.M.B.); lcgershony@ucdavis.edu (L.C.G.); trfamula@ucdavis.edu (T.R.F.); 2 Department of Clinical Sciences, Tufts Cummings School of Veterinary Medicine, North Grafton, MA 01536, USA; jerold.bell@tufts.edu; 3 Department of Medical and Clinical Genetics, Faculty of Medicine, University of Helsinki, 00014 Helsinki, Finland; marjo.hytonen@helsinki.fi (M.K.H.); hannes.lohi@helsinki.fi (H.L.); 4 Department of Veterinary Biosciences, Faculty of Veterinary Medicine, University of Helsinki, 00014 Helsinki, Finland; 5 Folkhälsan Research Center, 00290 Helsinki, Finland; 6 Department of Medical Biochemistry and Microbiology, Uppsala University, 752 37 Uppsala, Sweden; kerstin.lindblad-toh@imbim.uu.se (K.L.-T.); katarina.tengvall@imbim.uu.se (K.T.); 7 SciLifeLab, Uppsala University, 752 37 Uppsala, Sweden; 8 Broad Institute of MIT and Harvard, Cambridge, MA 02142, USA; 9 Bearded Collie Foundation for Health (BeaCon), Milner, GA 30257, USA; tillyrusty7@gmail.com

**Keywords:** Bearded Collie, inbreeding, homozygosity, pedigree, diversity, dog

## Abstract

**Background:** Genetic diversity in closed populations, such as pedigree dogs, is of concern for maintaining the health and vitality of the population in the face of evolving challenges. Measures of genetic diversity rely upon estimates of homozygosity without consideration of whether the homozygosity is desirable or undesirable or if heterozygosity has a functional impact. Pedigree coefficients of inbreeding have been the classical approach yet they are inadequate unless based upon the entire population. **Methods:** Homozygosity measures based upon pedigree analyses (*n* = 11,898), SNP array data (*n* = 244), and whole genome sequencing (*n* = 23) were compared in the Bearded Collie, as well as a comparison of SNP array data to a pedigree cohort (*n* = 5042) and a mixed-breed cohort (*n* = 1171). **Results:** Molecular measures based upon DNA are more informative on an individual’s homozygosity levels than pedigree analyses, although SNP coefficients of inbreeding overestimate the level of inbreeding based on the nature of SNP array methodology. Whole genome sequence (WGS) analyses revealed that the heterozygosity observed is generally in variants having neutral or low impact, which would indicate that the variability may not contribute substantially to functional diversity in the population. The majority of high-impact variants were observed in the shortest runs of homozygosity (ROH) reflecting ancestral breeding and domestication practices. As expected, mixed-breed dogs displayed higher measures of genomic diversity than either Bearded Collies or other pedigree dogs as a whole using the current paradigm algorithm models to calculate homozygosity. **Conclusions:** Using typical DNA-based measures reflect only a single individual and not the population thereby failing to account for regions of homozygosity that reflect ancestral breeding, domestication history, breed-defining regions, or regions positively selected for health traits. Incorporating measures of genetic diversity into dog breeding schemes is meritorious. However, until measures of diversity can distinguish between breed-defining homozygosity and homozygosity associated with positive health alleles, the measures to use as selection tools need refinement before their widespread implementation.

## 1. Introduction

Many posit that pedigree (purebred) dogs suffer from a degree of inbreeding that unnecessarily subjects them to a myriad of health disorders [1]. A recent publication eloquently summarized and catalogued the published papers detailing concerns surrounding pedigree dog breeding practices, highlighting that open registries allow for reduced levels of inbreeding [2]. However, they also point out that, despite an open registry, selection for breed-defining characteristics and the popularity of certain sires still plays a significant role in promoting inbreeding within a dog breed [2]. The genetic selection of dogs based on breeder preferences for particular traits has had a strong impact on the genetic structure of purebred populations for generations [3,4]. Inbreeding increases autozygosity through increased runs of homozygosity (ROH), reducing heterozygosity and permitting the expression of disorders controlled by recessive alleles [5]. Studies have associated the level of inbreeding within a breed with an elevated risk of inherited disorders [6,7,8,9], though some studies failed to find a significant association or found only a minimal association [10,11,12,13]. Selective inbreeding can also occur with the intentional removal of deleterious alleles which also increases autozygosity. The purging of deleterious alleles tends to be most successful within a population of limited genetic variability, such as following a genetic bottleneck, wherein the disorder has a higher likelihood of expression and purging may be achievable [14]. Regardless of intent, uniform selection for desirable traits and against observed instances of genetic disease and deleterious alleles will increase autozygosity.

Closed gene pools, popular sire effects, and consanguinity are typical in pedigree dog breeds and contribute to elevated measures of inbreeding. Inbreeding depression in several dog breeds has been correlated with reduced longevity and litter sizes [15,16] and balancing selection to maintain heterozygosity is observed in natural populations, although a recent study of the dog would indicate the “selection footprints” of balancing selection seem to be restricted to critical genomic regions adjacent to genes involved in cancer, immune function, and skeletal development regulation [17]. Thus, maintaining genetic diversity in domesticated canine populations is an important consideration for long-term health and viability, yet what measure of diversity (whether it be pedigree or DNA analysis) to use in breeding programs to achieve that objective is less clear. Especially when considering that genetic homozygosity for breed-defining traits is required to create a breed with phenotypic consistency. Furthermore, when health is emphasized in selection, genes regulating negative health traits may have less diversity, which is desirable. For example, in the Border Collie breed, ancestral inbreeding reduced hip and elbow dysplasia indicating a “purge” of deleterious mutations [18]. Stabilizing and purifying selection optimizes the realization of expected phenotype including positive health traits and when appropriately combined with balancing selection to maintain genetic diversity could optimize both [19]. Therefore, breeding goals must balance the maintenance of breed type, health advances, and functional diversity.

Functional diversity requires a knowledge of genetic variants that have an impact on gene expression, translation, or product function including cellular localization, protein–protein interactions, or secretory capacity. Single nucleotide polymorphisms (SNPs) in genomic DNA, while present and useful as genetic markers due to their variability between individuals, may not reflect substantive differences in overall gene function [20]. In humans, it is estimated that genomic DNA sequences differ approximately 0.1% between individuals with less than 1% of that variation impacting protein function [21].

Early practices to reduce the level of inbreeding within a dog breed relied upon pedigree analyses [11,22,23] with efforts to reduce inbreeding coefficients (*F_PED_*). Precise *F_PED_* relies upon accurate and complete pedigrees. The generational depth of pedigree information affects the ability to accurately identify individual dog homozygosity. The advent of SNP array technologies expanded the ability to assess inbreeding to the genetic level by characterizing ROH and individual SNP variability. Using SNP data, different dog breeds show a sequence diversity of 0.1% for intronic regions and 0.04% for coding regions [24], which matches the individual data for humans described above. A study comparing SNP data to pedigree inbreeding coefficient calculations found that the pedigree values underestimated genomic variability; however, generation depth and completeness of pedigrees were not indicated [25]. The availability of whole genome sequence (WGS) data permits an even greater accuracy of homozygosity determination and the possibility of ascribing functionality to the observed sequence variation.

In the present study, we sought to catalog the level of homozygosity and inbreeding coefficients as assessed by pedigrees, SNP array data, and WGS for the Bearded Collie breed and their relationship to breed genetic diversity. We evaluated these three modes of variation determination in a Bearded Collie population to identify how well these measurements align with one another and also compared those measures to those of pedigree dogs and mixed-breed dogs. We hypothesized that the genomic coefficient of inbreeding (*F_ROH_*) measurement would more accurately define inbreeding compared to pedigree-based measurements, and there would be differences between SNP and WGS measures.

As a corollary, we investigated the variants that were observed in the whole genome sequence data for their predicted impacts and possible functional variation within the breed as well as the homozygosity of putative high-impact variants.

## 2. Materials and Methods

### 2.1. Study Cohorts

An extended pedigree using 11,989 Bearded Collies representing 28.0 + 2.2 generations was available for analysis through the BeaCon Bearded Collie health database. The extended Bearded Collie pedigree was analyzed by the commercially available Pedscope version 2.5 pedigree program (Tenset Technologies Ltd., Cambridge, UK). Wright’s inbreeding coefficient (*F_PED_*) for all generations, and for the most recent 10- and 5-generation periods were calculated for the entire extended pedigree, and for a 23 Bearded Collie subset cohort. The number of unique ancestors in the individual pedigrees of the subset was also determined.

Owners or veterinarians submitted buccal swabs or blood samples from Bearded Collies. DNA extraction and quantification were carried out as previously described [26,27]. DNA samples were stored at −20 °C until further analysis. All dogs were housed and cared for by their owners. Investigators did not interact with or handle any of the dogs in the study. Samples from 244 Bearded Collies were genotyped using the 173k SNP Illumina CanineHD BeadChip (Illumina, San Diego, CA, USA). The cohort included 136 females and 108 males from various geographical locations (Australia and New Zealand (*n* = 10), North America (*n* = 146), and Europe (*n* = 88). Aliquots of DNA for 23 Bearded Collies (12 females and 11 males) from the 146 North American Bearded Collies used for SNP array analysis were submitted for whole genome sequencing (Novogene, Beijing, China). Within the subset of 23 WGS dogs, two were siblings, 9 shared a popular grandsire, and 14 were unrelated at the grandparent level.

### 2.2. SNP Data

To compare SNP data for the larger Bearded Collie cohort with pedigree and mixed-breed cohorts, publicly available 173k SNP Illumina CanineHD BeadChip genotype data were downloaded as PLINK binary files from the Dryad Digital Repository [28,29,30,31,32,33,34,35,36,37], the Lupa consortium [38], and our laboratory pedigree dog SNP data, including the 244 Bearded Collies. After an initial data merge in PLINK v.1.9 [39] using the --bmerge function, datasets with strand issues, identified in PLINK as SNPs with 3+ alleles, were converted to a dbSNP forward strand allele orientation using the open bioinformatics program convert_bim_allele.pl (https://www.openbioinformatics.org/gengen, accessed on 31 May 2022) using the perl command line (v5.32.1). The final merged dataset consisted of 12,402 dogs and 173,662 SNPs. Data filtering in PLINK removed duplicate individuals (--genome), individuals with less than 94% call rate (--mind 0.06), SNPs with less than 95% call rate (--geno 0.05), and any breed that had less than 15 samples. The standard individual call rate of 95% is often used in genome-wide association studies; however, an individual call rate of 94% was used for all cohorts to include as many individuals as possible. The filtering resulted in 158,378 autosomal SNPs for the 244 Bearded Collies, 156,693 autosomal SNPs for 5042 pedigree dogs representing 41 breeds, and 157,371 autosomal SNPs for 1171 mixed-breed dogs (consisting of mixed- and free-breeding dogs).

### 2.3. WGS Data

WGS was obtained for the 23 Bearded Collies using a PCR-free library with fragment sizes of 350 bp generating 150 bp paired-end reads with an average of 15× coverage using the Illumina HiSeq X™ Ten platform. Raw sequence data files were processed as previously described [40], following the Broad Institute’s Genome Analysis Tool Kit (GATK) best practices workflow for small germline variants [41]. Hard filters were applied to germline variants and indels separately. Using GATK’s best practices workflow for hard filtering, separate SNPs-only and indels-only subset files were created using GATK’s SelectVariants tool. Filter flags were added to variants with a phred score (QUAL) less than 30, QUAL score normalized by allele depth (QD) less than 2.0, strand odds ratio (SOR) greater than 3.0, Fisher strand (FS) value greater than 60, root mean square mapping quality (MQ) less than 40.0, mapping quality Rank Sum (MQRankSum) less than −12.5 and read position Rank Sum (ReadPosRankSum) less than −8.0. Filter flags were added to indel variants with QUAL less than 30, QD less than 2.0, FS value greater than 200.0, and ReadPosRankSum less than −20.0. A total of 207,956 flagged variants were removed using VCFtools version 0.1.17 [42], with 7,016,290 variants remaining for analysis.

To compare WGS data for the 23 Bearded Collie subset cohort with pedigree and mixed-breed cohorts, WGS variant data were accessed from the Dog Biomedical Variant Database Consortium (DBVDC) [43], which currently comprises variant calls from 804 dog and 9 wolf genomes. Bearded Collies, wolves, wolf-hybrids, and unknown breeds were removed from the DBVDC dataset leaving 762 dog samples for analysis. The two cohorts from the DBVDC dataset were created: pedigree dogs (*n* = 695 dogs representing 131 breeds) and mixed-breed dogs (*n* = 67 dogs defined as mixed and free-breeding dogs). The GATK best practices workflow was used for the whole genome variant calling and filtering [43,44], and VCFtools version 0.1.17 [42] was used to create variant files for each cohort with a total of 42,346,403 variants remaining for analysis.

Before PLINK analyses, whole genome vcf files for the 23 Bearded Collies and the DBVDC cohorts were converted to binary files (bed, bim, and fam) using PLINK v.2.0 [39] with parameters: --const-fid, --allow-extra-chr, --snps-only --max-alleles 2, --chr 1–38 and --set-all-var-ids @_#; the last parameter was necessary for downstream analyses and replaced all variant names listed in the vcf file from a period to chromosome and position. Data filtering removed duplicate individuals (--genome), individuals with less than 94% call rate (--mind 0.06), and variants with less than 95% call rate (--geno 0.05). The filtering resulted in 5,349,886 autosomal variants for the 23 Bearded Collies, 27,801,941 autosomal variants for 669 pedigree dogs (26 dogs were removed), and 25,181,113 autosomal variants for 65 mixed-breed dogs (2 dogs were removed).

### 2.4. ROH and F_ROH_

ROH is an effective method to estimate inbreeding in populations of interest [45]. *F_ROH_*, defined as the proportion of the complete genome held within ROH [46], is the preferred measure for dogs [47]. Although there are other ROH identification methods, such as Hidden Markov Models (HMMs) and identity-by-descent (IBD) haplotype analysis, the PLINK window-based approach was used to identify runs of homozygosity. This widely used method, commonly used in dogs and livestock species, identifies consecutive stretches of homozygous SNPs in individual samples for both SNP arrays and WGS [45,47,48,49,50,51,52]. To compare overall homozygosity and *F_ROH_* within and across the different dog cohorts, the ROH was calculated in PLINK [39] for the SNP and WGS variant data (Supplemental File S1). The minimum SNP and SNP window parameters were defined using a formula to calculate the average observed heterozygosity across individuals and SNPs to minimize false positives in each cohort [50,53,54,55]. The length of the SNP ROH was set at 1000 kb because that is believed to capture both recent and ancestral autozygosity [56] and to exclude short ROH due to linkage disequilibrium (LD) [55,57]. The length of the WGS ROH was set at 70 kb with zero heterozygotes to most closely generate comparable homozygosity analyses between the SNP array data and that of WGS as detailed in the published literature [58]. Each cohort’s average observed homozygosity and heterozygosity were calculated using PLINK’s --het option, where SNPs and variants scanned are non-missing, non-monomorphic autosomal variants. *F_ROH_* for both SNP and WGS data were calculated using the R package detectRUNS (version 0.9.6) [59] available through the public domain statistical language R [60]. The PLINK files for ped, map, and hom were read into the detectRUNS script (Supplemental File S1) for each cohort of Bearded Collies, the pedigree dog cohort, and the mixed-breed dog cohort. Significant differences were calculated using a one-way ANOVA in VassarStats [61]. For both SNP and WGS data, the quality control parameters of minor allele frequency (--maf), the Hardy–Weinberg equilibrium test (--hwe) and LD pruning (--indep) were not performed according to that described by Meyermans’ review of ROH parameters using PLINK [50]. However, to show differences between pruned and unpruned data, a *F_ROH_* comparison of the 23 Bearded Collie WGS subset cohort was performed using the PLINK function --indep 50 5 2 with similar ROH parameters defined in Dreger [58] (Table S1).

### 2.5. ROH Class Lengths and LD Decay

The ROH segments in the PLINK hom file for the Bearded Collie SNP data (*n* = 244) were divided into length classes: 1–2 Mb, 2–4 Mb, 4–8 Mb, 8–16 Mb, and >16 Mb and the *F_ROH_* was calculated for each length class using the R package detectRuns [59]. Genome coverage percentage was calculated using methods as detailed in the published literature [62,63]. The genome size used for the final genome percent calculation was based on the cohort’s total autosomal genome length calculated by detectRuns [59]. The dataset of 158,374 autosomal SNPs for the 244 Bearded Collie cohort was used to measure LD among SNPs per chromosome, calculated using the --r2 function in PLINK. The same methods from another canine study were used to calculate the LD decay, the effective population size (N_e_), and the increase in inbreeding (∆F) [64]. However, we allowed all possible SNP pairwise combinations within a 1 Mb window with the following PLINK functions --r2 --ld-window-kb 1000 --ld-window-r2 0 --ld-window 99,999 [65]. Average correlation coefficient (*r*^2^) values were sorted into bins [64] using the R ntile command in tidyverse [66] which sorts the list of base-pair counts and divides the number of lines by the desired bin size. The bins contained the same sample size with differing lengths between the minimum and maximum of each bin.

### 2.6. SNP F_ST_

The fixation index (*F_ST_*) [67,68] between the SNP data for the Bearded Collies (*n* = 244) and that for the mixed-breed dogs (*n* = 1171) was calculated using the --fst command in PLINK. The two datasets were merged in PLINK using the --bmerge command on the binary files (bed, bim, fam). A total of 164,068 SNPs were evaluated using a --within text file indicating the two cohorts to be evaluated: BC (Bearded Collie) and MIX (mixed breed). One study [69] suggests removing first-degree relatives using a --king-cutoff of 0.177 in PLINK to evaluate *F_ST_* between two populations. Sixty-two dogs were removed from the BC cohort and 26 dogs were removed from the MIX cohort. The --fst command was run on both datasets with and without first-degree relatives and *F_ST_* values were compared. There were no significant differences in *F_ST_* values whether the first-degree relatives were included or excluded (*p* = 1). Therefore, *F_ST_* values described herein are for the entire cohorts of BC and MIX including first-degree relatives. Using the recommendation of Cagan and Blass [70], *F_ST_* values above 0.75 were viewed as representing functional genomic variants. Ensembl gene names and positions (bp) are indicated for the CanFam3.1 reference genome.

### 2.7. SNP ROH Overlapping Windows

To determine SNP ROH shared by individuals within and across the Bearded Collie and mixed-breed cohorts, each chromosome was separated into 100 kb windows. A C++ program was developed to determine the SNP ROH shared by individuals within and across the Bearded Collie and mixed-breed cohorts. Using the PLINK hom files, the program sectioned each chromosome into 100 kb windows and counted the number of dogs with an ROH that overlapped or was contained within each window, ensuring distinct counts for the Bearded Collies and mixed breeds were maintained. The counts, expressed as a percentage of the sample size within each group of dogs, were plotted using the package ggplot2 [71] in R [60]. Regions having >90% overlap were then explored for genes that might be driving homozygosity. Ensembl gene names and positions (bp) are indicated for the CanFam3.1 reference genome.

### 2.8. WGS Variant Impact

All WGS variant files were annotated using SnpEff version 5.0e [72] based on the CanFam3.1 reference genome (Ensembl annotation version 104). VCFtools was then used to create variant files for each of the 23 Bearded Collies and for each DBVDC cohort (pedigree and mixed breed). SnpSift [73] was then used to extract genotypes for those variants with predicted high (e.g., frameshift variant), moderate (e.g., missense variant), low (e.g., synonymous variant), and modifier (e.g., 5′ UTR variant) impacts [72]. The low and modifier impact files were merged using bcftools version 1.15 [74] to create a “neutral” impact file. Variants with more than one predicted effect (i.e., missense and synonymous) could be present in different variant impact files; therefore, a single variant could be counted more than once.

Each individual Bearded Collie and the two DBVDC cohorts’ vcf impact files (high, moderate and neutral) were run in PLINK using the --geno-counts command to count the number of genotypes for each variant as homozygous reference, heterozygous, homozygous alternate, or missing genotype. Each geno-counts output file was then counted in R Studio [75] using R version 4.2.2 with a proprietary script to give overall totals for all variants for each of the 23 Bearded Collies or consortia cohorts for each impact file. To allow for consistency and comparison across vcf files, the alternate allele was considered the effect allele for each impact [76]. Percent homozygosity for each impact was based on the homozygote alternate counts for each dog or cohort compared to their overall total counts (homozygous reference, heterozygous and homozygous alternate, excluding missing variants). Due to the configuration of the available data from the DBVDC consortia, the counts of total and predicted impactful variants are identical for the pedigree and mixed-breed cohorts. Correlation coefficients (*r*^2^) between *F_ROH_* and percent impact, and ROH lengths, were calculated using a Pearson Correlation Coefficient calculator (https://www.socscistatistics.com/, accessed on 13 January 2025).

## 3. Results

**Figure 1:**
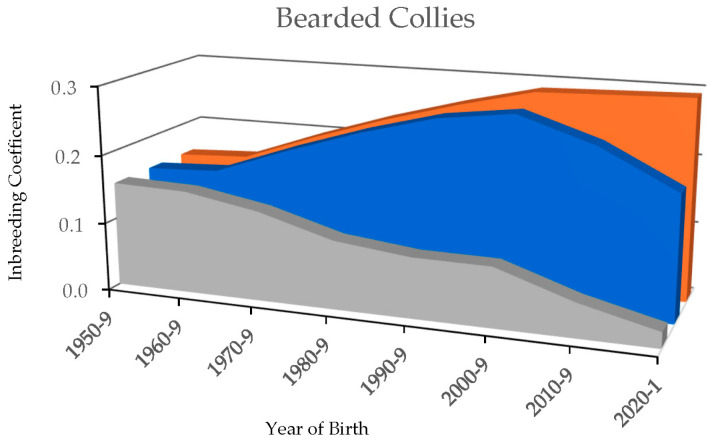
Change in pedigree-based inbreeding coefficients (*F_PED_*) over ten-year periods based on Bearded Collie pedigrees when the estimate included five (grey), ten (blue), or all possible (orange) generations.

The highest *F_PED_* from the pedigree analyses was observed when the full depth of available pedigree was included in the calculations and was statistically different from the *F_PED_* calculated from just 10 generations (*p* < 0.01, Table 1). The overall *F_PED_* estimate for the entire Bearded Collie cohort was 0.29 ± 0.04 presented as mean ± standard deviation (SD) with the individual dogs’ *F_PED_* values ranging from 0.21 to 0.41. The calculated *F_PED_* declined when fewer generations were included (Figure 1, Table S2). Restricting the calculations to the most recent five generations dramatically underestimated inbreeding coefficients and was most discordant with those derived from all generations, with an average of 0.07 ± 0.05 for the entire cohort and a range of 0.01 to 0.22 observed for individual dogs. Dogs with a greater number of unique ancestors in their pedigrees did not have the lowest *F_PED_* values.

The genomic inbreeding coefficient (*F_ROH_*) derived from assessing ROH in SNP array data exceeded the pedigree *F_PED_* calculations for every individual dog (*p* < 0.01, Table 2). In contrast, when *F_ROH_* inbreeding measures based on WGS variants were compared to the *F_PED_* for individual dogs, there were no obvious trends. For some dogs, the measures of inbreeding were higher when based on the WGS variants and in other cases lower than the *F_PED_* values. The *F_ROH_* based on SNP array data was consistently greater than the *F_ROH_* based on WGS variants. When the individual dogs’ values were averaged, the *F_ROH_* for WGS variants was not significantly different from that of the extended pedigree *F_PED_* nor significantly different than the SNP array data. However, the average *F_ROH_* for the SNP data significantly differed from the *F_ROH_* for the all-generation pedigree (*p* < 0.01, Table 2). The absolute number of ROH segments in either the SNP array data or the WGS data was not correlated with the *F_ROH_* having *r*^2^ correlation coefficients of 0.02 and 0.05 for array data and WGS data, respectively. However, the composite length of ROH as summed across the genome was remarkably similar for the SNP array and the WGS variants (Table 3, Table S3) with the average summed length of the ROH segments of 781,741 kb and 710,595 kb, respectively. In contrast, the average length of the ROH segments was much shorter for the WGS variant data (293 kb) than for the SNP array data (6854 kb) and the Bearded Collie average length of the ROH segment was 1.8-fold greater than that of the mixed breed and 1.3-fold greater than that for the pedigree cohort.

The average *F_ROH_* based on the SNP array data for the 244 Bearded Collies was calculated to be 0.31 ± 0.06 (Table 4 and Table S4), which was significantly less (*p* < 0.01) than that observed for the subset cohort of 23 Bearded Collies (0.35 ± 0.05) even though the majority of the dogs in the subset was unrelated at the grandparent level. Bearded Collies displayed higher *F_ROH_* than the pedigree cohort and both of those cohorts were significantly greater than that observed for mixed breeds. Bearded Collies also have similar ROH segments (*p* > 0.1) and observed homozygosity was significantly lower in the Bearded Collie subset than in the pedigree cohort (*p* < 0.01). Based on SNP data, the mixed-breed cohort had the lowest *F_ROH_* and higher heterozygosity compared to Bearded Collies and pedigree cohorts (all are significantly different from each other *p* < 0.01). In comparing the WGS cohorts, the Bearded Collies and the pedigree dog cohort have similar *F_ROH_*, although Bearded Collies are slightly higher (0.31 vs. 0.25, *p* < 0.01). Average *F_ROH_* values for the SNP array and WGS variant data for each breed in the pedigree cohort are shown in Table S5. Assessing the *F_ROH_* based on the WGS variant data revealed very similar inbreeding measures as that obtained by the SNP array data. The extent of homozygosity across the detected variants (SNPs and WGS variants) revealed that for SNPs, the pedigree cohort was the highest, the Bearded Collie cohort was intermediate and the lowest homozygosity was observed in the mixed breeds, although some mixed-breed individuals had homozygosity comparable to that seen in the pedigree dogs. In contrast, the Bearded Collies showed the least homozygosity for the WGS variants and the mixed breed was intermediate. Despite the reduction in individual variant homozygosity, the ROH segments for both the SNP array and the WGS variant data for Bearded Collies were approximately 125% longer than that for pedigree dogs and more than 350% longer than the mixed-breed dogs (Table 3).

ROH is another comparator used to assess genetic homozygosity and ancestral and recent inbreeding. Although differing across the study cohorts, within each cohort, that is for the Bearded collie, the pedigree, and the mixed-breed dogs, the average total ROH length summed over the entire genome was reasonably comparable in length for the SNP and WGS data (Table 3). The Bearded collie had a greater degree of the genome in ROH than the pedigree dogs and the mixed-breed dogs had the least mirroring what was seen for the average *F_ROH_*. The average lengths of the individual ROH were much less from WGS when compared to those derived from the SNP array data, though the trends of Bearded Collies having longer ROH than pedigree dogs which in turn had longer ROH than mixed-breed dogs were the same for both data sets. The number of ROH was equivalent in Bearded Collies and pedigree dogs for SNP array data whereas for WGS data, the number of ROH detected in Bearded Collies was less than that for pedigree dogs; the least number of ROH were detected in the mixed-breed cohort. 

Categorizing the individual ROH by class length can also provide insight into the historical age of the ROH in the population. For the SNP array data, ROH accounted for 31.38% of the variable genome in the Bearded Collie, with the greatest proportion of that coverage (40%) due to ROHs greater than 16 Mb in length (Table 5); the shortest length segments, although representing the least genome coverage, exhibited the highest number of ROH and the highest mean *F_ROH_* that was equivalent to the overall SNP *F_ROH_* seen in Table 4.

**Figure 2:**
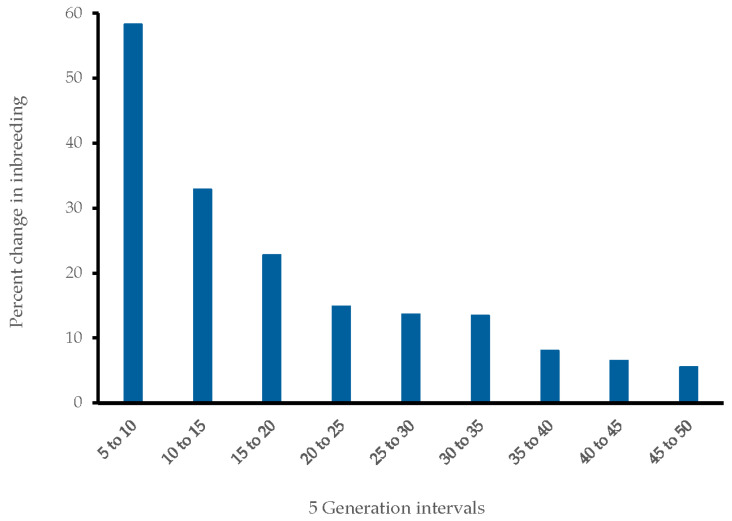
Percent change in inbreeding (∆F) per generation interval over time starting with the most recent interval from 5 to 10 generations ago.

The decay of LD as measured through correlation (*r*^2^) between SNP pairs can indicate loss of heterozygosity reflective of inbreeding. The maximum mean *r*^2^ in the Bearded Collie was 0.53 similar to that of the Dalmatian 0.56 [64] and the 50% decay was reached for SNPs 58.5 kb apart. For SNPs 1 Mb apart, the LD decreased to an average *r*^2^ value of 0.15 (compared to 0.11 in the Dalmatian). Over the most recent 50 generations, the N_e_ has been steadily declining with the maximum N_e_ at 78 down to an N_e_ average of 15 with the most recent generation. The increase in inbreeding (∆F) reached a maximum of 0.036 and a minimum of 0.021 in individual Bearded Collies in the most recent generation. Comparing inbreeding levels of 50 generations ago to the most recent 5 generations ago, based on SNP data, the change in inbreeding rose 367% (from 0.0061 to 0.0285) and 58.3% within the most recent generation interval of 10 generations ago to 5 (Figure 2).

To identify regions of commonality within and across the dog cohorts, which would indicate intentional conservation of homozygosity that may reflect either breed-defining or species-defining traits, the overlapping ROH were compared. All chromosomes showed overlapping ROH within the Bearded Collie breed and also with mixed-breed dogs (Figure S1). For instance, CFA18 showed an intergenic region wherein 50% of the mixed-breed dogs and 49% of the Bearded Collies had common overlapping ROH (Table S6). The chromosome having the highest overlap regions in the Bearded Collie was CFA 8 with more than 94% of the Bearded Collies and 42.5% of the mixed-breed dogs having ROH in this region. The probability of ROH overlapping in the Bearded Collie and mixed-breed dogs for CFA 8 is shown in Supplemental Figure S2. The highest proportion of overlap was in the first 18 windows of CFA8:72,077–1,869,862 which had a total of 743 windows of overlap and encompassed 24 unique characterized genes, a third of which were olfactory receptor genes (Table 6).

The SNP *F_ST_* for Bearded Collies was calculated relative to SNPs in the mixed-breed cohort to assess the regions that appear to be at or near fixation in the Bearded Collie which may reflect breed-defining regions or areas of reduced heterozygosity indicative of selection. The highest *F_ST_* values were for SNPs BICF2P1167655 (*F_ST_* = 0.94) and BICF2P1256259 (*F_ST_* = 0.92), both on CFA8 (Table S7). These SNPs are located in an intergenic region 16 kb upstream of a *U4* snRNA gene and 227 kb upstream of *NOVA1*, respectively. Seventeen chromosomes had one or more SNPs with *F_ST_* values greater than 0.75 and both *CLEC2D* and *CRADD* genes were represented by more than one SNP.

With the goal of assessing the functional impact of the variation observed in WGS across the cohorts, the number and predicted impact of the variants were determined for individual Bearded Collies, pedigree dogs, and mixed-breed dogs. The proportion of those impactful variants that were homozygous was also calculated (Table 7). Overall counts for each impact category are given for the 23 Bearded Collies (Table S8). The vast majority of variants (>99%) were predicted to have a neutral impact in all three cohorts. The *r^2^* correlation between the *F_ROH_* and % homozygous high-impact variants was 0.42, and for % homozygous moderate impact variants was 0.62 and for % homozygous neutral impact variants was 0.89 suggesting that much of the inbreeding observed was captured in homozygosity of variants predicted as having neutral impact. Of those having high impact, 12.7% were homozygous for the alternate, impactful allele in the Bearded Collie whereas the percentage was much lower for the pedigree dogs as a whole and very similar between the pedigree and mixed-breed cohorts (2.03 and 1.77, respectively). This profile was quite similar for the moderate impacts (13.1, 1.93, and 1.69, for Bearded Collies, pedigree breeds, and mixed breeds, respectively). The homozygosity for variants with neutral impact was nearly double what was observed for either high and moderate impact, and the Bearded Collies represented ~23%. Of the high-impact variants, only 30 were in ROH and all were in the shortest ROH length class.

## 4. Discussion

There is little controversy that functional diversity is important for genomic resilience in the face of changing environmental conditions including pathogen exposures. Recent discussions advocating for genomic diversity cite improved health, greater litter size and avoidance of inbreeding depression and reduced lifespan [16]. Several recent studies have assessed genetic diversity in various dog breeds and their impact on health [11,77,78,79,80,81,82,83]. Yet defining the best measure of genomic diversity is less clear. Simply cataloging overall homozygosity fails to account for genomic regions harboring breed-defining traits that are necessarily homozygous in order for a breed to perpetuate those traits to subsequent generations. Or that particular chromosomal regions must be homozygous for cell functionality and organismal viability (centromeres, genes essential for cell division, etc.). Current analytical pipelines that measure genomic diversity exclude monomorphic variants; therefore, only variable regions are used to estimate homozygosity and inbreeding [84]. It is estimated that 98–99% of the dog’s genome is monomorphic [49,85] and protein-coding sequence accounts for about 1% [86]. The canine SNP array targets SNPs that exhibit high polymorphism across breeds [85] and have a measurable population minor allele frequency, resulting in an underrepresentation of rare SNPs with an overestimate of more common SNPS, both of which can contribute to an overestimation of homozygosity. Additionally, SNP-based diversity assessments utilizing non-coding DNA SNPs will not account for the fact that non-coding DNA is subjected to greater evolutionary change and, therefore, will show greater heterozygosity, yet those variants present may not reflect functional variation in the genome [87]. Whole genome sequencing avoids bias inherent in SNP results [84], because WGS accommodates rare variants and, more importantly, breed-defining variants, which are more likely to be revealed with its higher density of variants that the SNP array does not capture.

In dogs, inbreeding coefficient calculations based on pedigrees, although rather simple to obtain and historically relied upon, tend to be of limited utility due to the imperfect knowledge of the ancestors beyond a few generations as well as the possibility of incorrect parentage. The approach of using *F_PED_* calculations based on the frequency of ancestors in the Bearded Collie pedigrees grossly underestimated inbreeding when a few generations were included in the calculation. The *F_PED_* for Bearded Collies based upon all available ancestors was nearly six times greater than that from only a five-generation pedigree and better aligns with that derived from SNP and WGS data. The calculated *F_PED_* within the breed inclusive of all ancestors exceeds that expected from father–daughter or full-sib mating, yet relying on the *F_PED_* for a five-generation pedigree would suggest much greater diversity than actually exists. One study reported that the majority of breeds show a declining trend of inbreeding in recent generations [58], reflecting what was observed from 2000 to 2021 in the present Bearded Collie study based on pedigree data, indicating efforts to intentionally avoid breeding close relatives. However, the degree of genetic diversity within a breed is often determined at the initial stages of breed development and despite recent efforts, population inbreeding coefficients continue to increase. Another study reported that recent breeding practices when compared to the past selection practices have negatively impacted canine health [9] although the authors also note that the current population inbreeding load likely reflects the level of genetic diversity in the dogs used in breed derivation. Using pedigree inbreeding coefficients based on eight generations, it was reported that inbreeding levels and health were not correlated in a closed colony of Labrador retriever guide dogs [10]. Whether the calculated non-inbred dogs were or were not less genetically related to the inbred dogs at the genomic level is unknown in those Labradors, but the health conditions being uniformly distributed supports the concept that the population health, in general, reflects that of the founder dogs of the breed.

More accurate measures of homozygosity are based on DNA data. It is well established that genomic inbreeding coefficients exceed those based on pedigrees [88] and this was observed in the present data. Including more generations of Bearded Collies resulted in a pedigree-based measure of inbreeding more similar to the genomic estimate derived from SNP array data or WGS, although they still were more than a 10% lower estimate. When comparing measures of inbreeding from DNA data, the estimate derived from the SNP array data overestimates the inbreeding when compared to the WGS. Measures of homozygosity, and, therefore, inbreeding coefficients, defined from SNP data, will always be an overestimate because the lengths of ROH are greater in SNP array data. That is a result of the configuration of the SNP arrays [48]. True heterozygous variants that lie between two homozygous SNPs will fail to be recognized and the stretch between those two SNPs will be called as a single long ROH suggesting greater homozygosity than actually exists. Those heterozygous loci would, however, be detected by WGS and result in shorter ROH in WGS data as was corroborated by the greater number of observed shorter ROH lengths in WGS data.

Genomic inbreeding coefficients (*F_ROH_*) based on SNP array data in other domesticated species range from a high of 0.48 in highly specialized horse breeds [89] to 0.23 for a closed line of beef cattle [90] to 0.09 in sheep [91]. In pedigree dogs, many breeds exhibit similar if not greater *F_ROH_* to the Bearded Collies in the present study although some breeds do have values in the 0.16 range [9,47,55,77,92] and Table S5. A composite WGS *F_ROH_* from 15 herding breeds was estimated at 0.29 [93] and 0.26 based on a single Bearded Collie [58] were both slightly lower than the average *F_ROH_* we report here for a greater number of Bearded Collies that were whole genome sequenced (though individual Bearded Collies ranged from 0.18 to 0.43). Published *F_ROH_* based on village dogs (0.09 and 0.12) and Chinese Indigenous dogs (0.05) [49,93] was very similar to that reported here for mixed breeds.

Subramanian and Kumar [94] found that a WGS *F_ROH_* greater than 24% was associated with higher morbidity than for breeds with a WGS *F_ROH_* of 10%. Interestingly, though the level of inbreeding might be considered elevated compared to some other breeds [47], the Bearded Collie has a lifespan in the top 15% of the breeds evaluated [95], and exceeded that of the mixed breeds included in that study. Of note from the MacMillan study, several breeds had lower median survival ages, such as the Siberian Husky, the Beagle, and the Chihuahua, and were reported in the literature as having low WGS-based *F_ROH_* values of 0.084 to 0.1 [58]. This reinforces the concept that recent inbreeding is insufficient to explain the observed accumulation of deleterious alleles, which suggests that there are ancient, historical explanations [96]. In agreement with that concept, the shortest lengths of ROH in the Bearded Collie, reflective of ancestral breeding practices, appear to drive the overall population *F_ROH_* despite the highest proportion of the ROH being in long ROH, which are indicative of more recent inbreeding events.

Regions of substantial shared ROH are viewed as “targets of positive selection” resulting in decreased heterozygosity for selected loci [97]. Analysis of human populations suggests that regions of abundant ROH are not uniformly distributed across the genome and represent regions of positive selection and, in some cases, purifying selection against variants responsible for autosomal dominant Mendelian disease [97]. Purposeful outbreeding can disrupt advances made for health selection as well as other adaptive gene clusters, referred to as outbreeding depression in the conservation literature [98]. Areas of ROH in domesticated animals, more particularly breeds within a domesticated species, likely reflect selection for breed-defining traits [91], although ROH may be well-tolerated in the absence of selection pressure if deleterious variants are absent in the region. Thus, while ROHs are associated with increased morbidity [94] and disease [47], they may also reflect important regions of homozygosity for breed-defining or health-based traits as well as regions having limited negative impact and regions under purifying selection [14,18,23,99]. Vigorous selection for particular traits and breed development can, however, result in genetic bottlenecks and excessive accumulation of deleterious gene variants that may compromise a breed. Clear examples can be seen in dog breeds such as the Norwegian Lundehund, suffering from intestinal problems along with cancer, eye, and heart diseases [100,101].

The longer segments of ROH in the Bearded Collie would suggest that the observed inbreeding reflects recent inbreeding practices [47,102] supported by a rise in overall inbreeding in the most recent time frames. As noted above, inbreeding coefficients will also rise with concerted efforts to purge deleterious and undesirable alleles [14], which may be the case with the Bearded Collie. Historically, the first specific reference to the Bearded Collie as a distinct breed name was in 1891, with litters registered in the early 1900s [103], and the breed was recognized by The Kennel Club in the UK in 1959 [104]. The inbreeding data, aligning with the historical accounts, suggest that prior to the 1930s, the Bearded Collie was inbred at a level equivalent to a mixed-breed dog and likely lacked the cohesion expected of a purebred dog. The degree of inbreeding and loss of heterozygosity is a rather recent phenomenon as evidenced by the ROH and LD decay data. Other pedigree dog breeds having a longer historical background, such as the Dalmatian, exhibit a similar pattern of inbreeding [64] suggesting 20th-century breeding practices to refine breed characteristics spanned across the breeds resulting in similar levels of homozygosity, albeit in breed-specific patterns.

Overlapping regions of ROH within the Bearded Collie breed revealed regions under positive selection. The genes *RSPO2* and *FGF5* are genes involved in furnishings and coat length, respectively [105], and have also been seen in domestic dog selective sweeps [106]. The Bearded Collies showed high ROH overlap (82–84% of the dogs) in three ROH windows spanning the *RSPO2* gene. One window spanning the *FGF5* gene showed less overlap, where 35% of Bearded Collies and 10% of mixed-breed dogs had ROH. Assessing the *F_ST_* of particular SNPs also demonstrated regions of positive selection. One such SNP lies within *MITF*, a gene responsible for coat color white spotting, and the Bearded Collie breed standard specifies dogs can be born with or without white markings [107]. The genes *CLEC2D* and *CRADD* were represented by more than one SNP in the *F_ST_* results. *CLEC2D* is involved in cell death recognition [108] and *CRADD* is involved in neuronal apoptosis [109]. One chromosome, CFA 10, demonstrates substantial evidence of selective sweep patterns across many different dog breeds [110]. *FSHR* is located on CFA 10 and is known to regulate litter size in pigs and goats [111,112]. It was seen to be highly homozygous in the Bearded Collie and has been the target of selection signatures for domestication in other species [113,114,115]. Interestingly, only three SNPs (one each on CFA 2, 19, and 29) exhibiting *F_ST_* values indicative of positive selection fell within ROH segments and two of those ROH segments were completely intergenic, but the region on CFA 29 contained the *NSMAF* gene that functions in inflammation and apoptosis [116]. A common ROH across all dogs was observed on CFA 8 harboring conserved genes that reflect olfactory function, as was shown across the species [117] and within the dog [118], again reinforcing the view that ROH may reflect critical biological functionality.

Among the Bearded Collie cohort, the number of high-impact variants present in the dogs was roughly equivalent despite different individual dogs having markedly different levels of *F_ROH_* inbreeding determinations. However, those high-impact variants that were present in a homozygous state did differ across individual dogs (11.0% to 14.6%) and that coupled with individual Bearded Collies having WGS *F_ROH_* values as low as 0.18 suggests that there exists variation in the breed that could be leveraged in breeding schemes. To assess functional diversity, the impact of the observed variants in the WGS were catalogued. Most of the variants were of neutral impact, and they were twice as likely to be homozygous as either the high or moderate impact variants suggesting that an elevated homozygosity in some dogs may not reflect functional impact. The presence of retrocopies in a genome that is rarely expressed and thus not subjected to selection can also accumulate variants although genetic diversity in those regions may or may not be significant for the health of the animal [119,120]. What also needs to be considered is whether in the absence of an ROH, suggesting greater genetic variability, the actual composition of the genes is the same. In other words, there may be lower *F_ROH_* but there may be the exact same functional gene sequences and thus functional variability would be the same despite seemingly greater heterozygosity.

The rationale for applying diversity measures to dog breeding is often modeled on conservation plans for endangered species (species survival plans or SSP) [121]. The objective of an SSP program is to maximize genetic diversity within a particular captive species for conservation goals which is not the same objective of pedigree dogs which, by intention, have limited genetic diversity for breed-defining traits. In doing so, deleterious variants are spread throughout the population and result in sporadic, unpredictable expression of disease. Sporadic expression impedes dog breeders from purposefully avoiding deleterious health conditions. Calls to outbreed to enhance genetic diversity to preserve modern day dog breeds [9,15] fail to account for breed-defining homozygosity, homozygosity following intentional purging of deleterious alleles, and that outbreeding will result in a dispersion of deleterious alleles across the entire population that then will make it difficult to intentionally avoid breeding dogs carrying undesirable variants. It was noted that bottlenecks, for example, the original domestication process or breed derivation while elevating “genetic load” can be managed through selection practices that are holistically applied to avoid the accumulation of deleterious variants [122]. A Finnish study reported that 87.5% of 250 impactful variants were seen across all dogs and although pedigree dogs may have greater homozygosity, 57% of all dogs possessed at least one copy of a deleterious allele [78,123] illustrating that some of the deleterious variants are very ancestral in the formation of the dog; the LD decay data here supports the concept that ancestral breeding is the greatest contributor to *F_ROH_* as does the impactful variants found in the shorter ROH. Another recent study found that purposefully crossbreeding pedigree dogs to form “designer breeds” further exemplifies this [124]. They found that purposeful crossbred dogs were not better or worse for health traits than their pedigree parent breeds. A shorter ROH is indicative of more ancestral breeding practices [47,93] and the finding that the high-impact variants found in ROH were all in the short ROH supposes these are genetic variants that accompany early dog/breed formation. Thus, whereas it sounds reasonable to suggest outbreeding and heterozygosity for a breed’s genetic diversity and to prevent the expression of deleterious genes, practically speaking the goal of pedigree dog breeding is to create homozygosity of positive breed-specific traits and, importantly, health-related traits through targeted selection.

One must be aware that the current measures of homozygosity or diversity reflect the status of an individual animal and not the structure of the population. As shown in the present data, even within a breed, there are individuals having substantial differences in their measures of inbreeding and homozygosity with impactful variants underscoring the need to reflect on the variability present in the breed population when assessing single animals.

Utilizing measures of inbreeding to reduce loss of diversity in the breeding population is a vital tool provided the measures used are both accurate and truly informative. These data raise questions as to how breed genetic diversity is being measured, and its relationship to the measurements of homozygosity. Individual dog homozygosity is based on both breed-wide ROHs that are breed-defining, plus any ROH from the genetic relationship between the parents. Breed-wide ROHs reflect breed-defining selections that are distinct from the parental relationship (inbreeding) of the dog being analyzed. Importantly, current approaches to measuring homozygosity do not differentiate between positively selected homozygosity versus homozygosity that may harbor deleterious alleles. Different breeds based on the genetic relationship of their founders may have higher or lower average homozygosity, but that does not necessarily relate to their genetic health or lifespan, which is based on the breed’s genetic load of deleterious variants. It is only through the selection for health and against disease-associated variants that genetic disease can be prevented and reduced in frequency to improve breed health.

## 5. Conclusions

In conclusion, inbreeding estimates for a given dog based upon SNPs tend to overestimate that seen at the whole genome level. Additionally, the pedigree coefficient of inbreeding calculations based on five generations grossly underestimates the actual degree of relatedness and should not be relied upon to inform breeding decisions to address reduced genetic diversity in a breed; much more accurate estimations are based upon all generations of an individual dog. Single nucleotide polymorphisms are defined as those more frequent within a population, potentially germline transmitted, and being observed in more than one percent of the population. In contrast, a single nucleotide variant or genetic variant identified in WGS may be abundant within a population, or it may reflect an individual’s specific variation and not contribute to the overall population variance, or diversity, through germline transmission, which may be responsible for the lower estimates of homozygosity when looking at WGS measures. What this reflects is that neither DNA measure perfectly captures genetic diversity and more importantly, functionality. Nor do such measures account for the fact that prevalent ROH includes selection signatures that are breed-defining. SNP arrays are more financially accessible but overestimate inbreeding and it is imperative to have valid measures when incorporating diversity into breeding schemes. Incorporating measures of “genetic diversity” into breeding schemes requires accurate and informative tools. Genetic diversity within regions of the genome that are impactful while still accommodating breed-defining homozygosity is vital and the currently available measures do not account for these nuances associated with homozygosity. More work into the algorithms to define DNA diversity is necessary before widespread implementation. Especially considering that DNA measures reflect that of an individual and not the population as a whole.

## Figures and Tables

**Table 1:** Calculated pedigree-based inbreeding coefficient (*F_PED_*) for 23 individual Bearded Collies and the entire available cohort (*n* = 11,989) for all generations available, ten generations, and five generations. Averages are presented as mean ± standard deviation (SD).

Dog ID	All Possible Generations	Unique Ancestors	All Generation *F*_PED_	Ten Generation *F*_PED_	Five Generation *F*_PED_
BC1570	23	192	0.31	0.31	0.13
BC1696	26	247	0.29	0.28	0.09
BC1838	26	256	0.31	0.30	0.11
BC1852	27	344	0.32	0.27	0.13
BC1893	28	399	0.27	0.23	0.06
BC1894	28	289	0.27	0.25	0.05
BC1895	26	270	0.26	0.24	0.05
BC1896	23	192	0.31	0.31	0.13
BC1899	25	289	0.25	0.24	0.04
BC1901	30	504	0.29	0.23	0.01
BC1904	24	196	0.33	0.33	0.07
BC1905	27	368	0.26	0.22	0.05
BC1909	29	425	0.32	0.23	0.04
BC1911	27	331	0.28	0.23	0.01
BC1916	27	405	0.25	0.23	0.01
BC1918	27	270	0.34	0.29	0.11
BC1936	26	396	0.27	0.26	0.07
BC1943	23	266	0.21	0.21	0.03
BC1944	25	219	0.29	0.28	0.11
BC1945	28	317	0.29	0.27	0.07
BC1952	30	557	0.27	0.15	0.01
BC0057	27	253	0.41	0.39	0.22
BC0058	29	502	0.28	0.17	0.05
Average Individual	26.5 ± 2.1	326 ± 104	0.29 ± 0.04	0.26 ± 0.05	0.07 ± 0.05
Average Population	28.0 ± 2.2	432 ± 134	0.29 ± 0.05	0.24 ± 0.06	0.05 ± 0.05

**Table 2:** Comparison of measures of inbreeding based on pedigree calculations, SNP array data, and WGS variant data. Averages are presented as mean ± SD.

Dog ID	All Generation *F*_PED_	*F_ROH_* for SNPs	*F_ROH_* for WGS
BC1570	0.31	0.37	0.27
BC1696	0.29	0.39	0.35
BC1838	0.31	0.35	0.34
BC1852	0.32	0.37	0.36
BC1893	0.27	0.43	0.40
BC1894	0.27	0.32	0.24
BC1895	0.26	0.34	0.33
BC1896	0.31	0.38	0.36
BC1899	0.25	0.28	0.18
BC1901	0.29	0.31	0.30
BC1904	0.33	0.44	0.39
BC1905	0.26	0.31	0.28
BC1909	0.32	0.31	0.29
BC1911	0.28	0.32	0.30
BC1916	0.25	0.35	0.33
BC1918	0.34	0.45	0.40
BC1936	0.27	0.34	0.33
BC1943	0.21	0.24	0.22
BC1944	0.29	0.31	0.29
BC1945	0.29	0.36	0.34
BC1952	0.27	0.38	0.37
BC0057	0.41	0.46	0.43
BC0058	0.28	0.35	0.35
Average	0.29 ± 0.04 ^a^	0.35 ± 0.05 ^b^	0.32 ± 0.06 ^ab^

Means carrying different superscripts indicate they significantly differ *p* < 0.01.

**Table 3:** SNP array and WGS cohort averages ± SD for total length of runs of homozygosity (ROH), length of ROH, and number of ROH.

Cohort	SNP Array Avg Total ROH Length (kb)	WGS Avg Total ROH Length (kb)	SNP Array Avg ROH Length (kb)	WGS Avg ROH Length (kb)	SNP Array Avg ROH	WGS Avg ROH
Bearded Collie	691,315 ± 122,637 ^b^	ND ^	6586 ± 1101 ^a^	ND	105 ± 12 ^a^	ND
Bearded Collie (subset)	781,741 ± 119,771 ^a^	710,594 ± 133,720 ^a^	6854 ± 1122 ^a^	293 ± 64 ^a^	114 ± 9 ^a^	2468 ± 392 ^b^
Pedigree	543,716 ± 205,664 ^c^	570,736 ± 240,100 ^b^	5060 ± 1339 ^b^	190 ± 53 ^b^	106 ± 28 ^a^	2989 ± 1062 ^a^
Mixed breed	187,952 ± 230,523 ^d^	150,968 ± 167,839 ^c^	3654 ± 3473 ^c^	141 ± 50 ^c^	43 ± 20 ^b^	924 ± 563 ^c^

Means within a column carrying different superscripts indicate they significantly differ *p* < 0.01. ^ ND: not performed.

**Table 4:** Average *F_ROH_* ± SD (range), observed homozygosity (O_HOM_) in four dog cohorts based on SNP array and WGS variant data.

Cohort	Total Genotyped Dogs	SNP *F_ROH_*	SNP O_HOM_ (%)	Total WGS Dogs	WGS Variant *F_ROH_*	WGS Variant O_HOM_ (%)
Bearded Collie	244	0.31 ± 0.06 ^c^ (0.17–0.51)	71 ± 2 ^b^ (64–79)	ND ^	ND ^	ND ^
Bearded Collie (subset)	23	0.35 ± 0.05 ^d ^ (0.24–0.46)	68 ± 3 ^d ^ (63–74)	23	0.32 ± 0.06 ^c^ (0.18–0.43)	65 ± 2 ^a^ (62–70)
Pedigree	5042	0.25 ± 0.09 ^b^ (0.01–0.62)	74 ± 3 ^c^ (65–87)	669	0.26 ± 0.11 ^b^ (0.01–0.59)	90 ± 2 ^c^ (82–94)
Mixed breed	1171	0.09 ± 0.10 ^a^ (0.01–0.49)	68 ± 4 ^a^ (61–82)	65	0.07 ± 0.07 ^a^ (0.01–0.51)	84 ± 2 ^b^ (82–92)

Means within a column carrying different superscripts indicates they significantly differ *p* < 0.01. ^ ND: not performed.

**Table 5:** The average number of ROH and *F_ROH_* for different length classes for 244 Bearded Collie SNP array data.

Length Class in Mb (Average Length Within That Class)	Average Number of ROH per Dog in Class	Genome Coverage (%) in Class	Mean *F_ROH_* (± SD) in Class
1–2 (1.41)	34.87	2.23	0.31 ± 0.06
2–4 (2.84)	23.40	3.02	0.29 ± 0.06
4–8 (5.68)	20.47	5.27	0.26 ± 0.06
8–16 (11.26)	16.08	8.22	0.21 ± 0.06
>16 (26.39)	10.55	12.63	0.13 ± 0.05
Total		31.38	

**Table 6:** ROH overlapping SNP windows greater than 90% presented in order from highest to lowest percent in the Bearded Collie cohort (*n* = 244) compared to the mixed-breed cohort (*n* = 1171). Ensembl gene names and positions (bp) are indicated for the top regions for the CanFam3.1 reference genome.

CFA	Window Number	Overlapping Window Start Position (bp)	Overlapping Window End Position (bp)	Total Bearded Collies	BC %	Total Mixed Breed	Mixed Breed %	Ensembl Genes in the Overlapping Region
8	2	171,954	271,830	234	95.9	421	36.0	*ZNF496*,* NLRP3*
8	3	271,831	371,707	234	95.9	421	36.0	*NLRP3*,* OR2H10*
8	4	371,708	471,584	234	95.9	421	36.0	*OR2B11*,* OR2W11*,* GCSAML*,* OR2C3*
8	5	471,585	571,461	234	95.9	421	36.0	*GCSAML*
8	6	571,462	671,338	234	95.9	423	36.1	*OR6AA6*,* ENSCAFG00000055652*
8	7	671,339	771,215	234	95.9	423	36.1	*ENSCAFG00000029235*,* OR6C4*,* ENSCAFG00000042211*
8	8	771,216	871,092	234	95.9	426	36.4	*OR4L1*
8	9	871,093	970,969	234	95.9	427	36.5	*CATSPERB*
8	10	970,970	1,070,846	234	95.9	471	40.2	*CATSPERB*
8	11	1,070,847	1,170,723	234	95.9	472	40.3	*TC2N*,* FBLN5*
8	12	1,170,724	1,270,600	234	95.9	498	42.5	*FBLN5*,* TRIP11*
8	13	1,270,601	1,370,477	234	95.9	453	38.7	*TRIP11*,* ATXN3*,* CPSF2*,* ENSCAFG0000005868*
8	16	1,570,232	1,670,108	233	95.5	377	32.2	*SLC24A4*
8	17	1,670,109	1,769,985	233	95.5	441	37.7	*SLC24A4*,* RIN3*,* LGMN*
8	18	1,769,986	1,869,862	233	95.5	472	40.3	*RIN3*,* LGMN*
8	1	72,077	171,953	232	95.1	415	35.4	*ENSCAFG00000046615*,* ZNF496*
8	14	1,370,478	1,470,354	231	94.7	381	32.5	*CPSF2*
8	15	1,470,355	1,570,231	231	94.7	363	31.0	*SLC24A4*

**Table 7:** Proportion of WGS high, moderate, and neutral impact variants for Bearded Collies (*n* = 23), a mixed-breed cohort (*n* = 67), and a pedigree cohort (*n* = 695). Variant files were annotated to the CanFam3.1 reference genome.

Dog ID	Total Variants	Total HIGH-Impact Variant Count	Percent Homozygous Alternate HIGH-Impact Variants	Total MODERATE Impact Variant Count	Percent Homozygous Alternate MODERATE Impact Variants	Total NEUTRAL Impact Variant Count	Percent Homozygous Alternate NEUTRAL Impact Variants
BC1570	7,940,287	4494	12.9	29,435	13.2	7,906,358	22.9
BC1696	8,113,127	4505	12.0	29,454	13.0	8,079,168	23.5
BC1838	8,145,732	4577	12.6	29,637	12.6	8,111,518	23.0
BC1852	8,140,001	4565	13.1	29,554	13.1	8,105,882	24.2
BC1893	8,112,085	4527	12.8	29,484	14.4	8,078,074	25.2
BC1894	7,990,385	4532	13.0	29,581	13.2	7,956,272	22.1
BC1895	8,121,517	4537	12.8	29,525	12.6	8,087,455	22.6
BC1896	8,127,437	4550	11.7	29,539	12.7	8,093,348	23.4
BC1899	7,964,369	4503	11.0	29,492	11.8	7,930,374	20.6
BC1901	8,145,187	4590	11.9	29,633	12.0	8,110,964	22.6
BC1904	8,133,943	4553	14.2	29,415	13.8	8,099,975	25.1
BC1905	8,130,514	4538	13.0	29,490	13.0	8,096,486	22.6
BC1909	8,106,692	4503	12.5	29,362	12.2	8,072,827	22.4
BC1911	8,134,998	4576	12.3	29,589	12.7	8,100,833	22.2
BC1916	8,138,035	4547	11.7	29,499	13.0	8,103,989	23.3
BC1918	8,128,449	4556	14.6	29,449	14.6	8,094,444	25.6
BC1936	8,144,075	4597	13.7	29,622	13.9	8,109,856	23.6
BC1943	8,109,118	4475	11.3	29,395	11.3	8,075,248	20.7
BC1944	8,118,503	4513	11.5	29,506	12.6	8,084,484	22.1
BC1945	8,103,332	4450	11.9	29,333	13.0	8,069,549	23.2
BC1952	8,142,011	4581	13.9	29,586	13.8	8,107,844	24.0
BC0057	8,131,063	4555	14.6	29,531	14.8	8,096,977	26.0
BC0058	8,135,341	4584	13.5	29,635	13.5	8,101,122	23.5
Average Bearded Collie	8,106,791	4539	12.7	29,511	13.1	8,072,741	23.2
Average Pedigree	42,346,403	43,580	2.03	209,404	1.93	42,093,419	3.75
Average Mixed breed	42,346,403	43,580	1.77	209,404	1.69	42,093,419	3.29

## Data Availability

The original contributions presented in this study are included in the article/Supplementary Materials. Further inquiries can be directed to the corresponding author.

## Supplementary Materials

The following supporting information can be downloaded at: https://www.mdpi.com/article/10.3390/genes16040378/s1, Supplemental File S1: PLINK ROH and R detectRuns parameters; Figure S1: The probability of SNP ROH overlaps in 100,000 bp windows for Bearded Collie (*n* = 244) compared to mixed breed (*n* = 1171) across all autosomes (CFA1—38); Figure S2: The probability of SNP ROH overlaps for CFA8 in 100,000 bp windows for Bearded Collie (*n* = 244) compared to mixed breed (*n* = 1171); Table S1. *F_ROH_* for 23 Bearded Collie WGS variants (*n* = 5,349,886) compared to *F_ROH_* for pruned variants (*n* = 242,880) using the PLINK function --indep 50 5 2. The same PLINK ROH parameters were used on both datasets except for the minimal number of SNPs to call an ROH calculated using a formula proposed by Lencz [54]; Table S2. Data generated from the pedigree analysis of 11,989 Bearded Collies. The colors (gray, blue, and orange) correlate to 5, 10, and all possible generations, respectively, in Figure 1; Table S3. Individual Bearded Collie SNP array data and WGS data for total autosomal variants, total length of ROH, average length of ROH, and number of ROH segments per dog; Table S4. *F_ROH_* for SNP array data for 244 Bearded Collies. *F_ROH_* for WGS data for the 23 Bearded Collies is noted for comparison; Table S5. *F_ROH_* for SNP array data and WGS variants averaged across breeds. SNP array PLINK files were downloaded from the Dryad Digital Repository [28,29,30,31,32,33,34,35,36,37], the Lupa consortium [38], and WGS data was accessed from the Dog Biomedical Variant Database Consortium (DBVDC) [43]; Table S6. The highest ROH SNP windows overlapping between the Bearded Collie cohort (*n* = 244) and the mixed-breed cohort (*n* = 1171). Ensembl gene names and positions (bp) of the SNPs are based on the CanFam3.1 reference genome, a dash indicates an intergenic region; Table S7. SNP *F_ST_* values greater than 0.75 for Bearded Collie (*n* = 244) relative to mixed breed (*n* = 1171). Ensembl gene names and positions (bp) of the SNPs are based on the CanFam3.1 reference genome, a dash indicates an intergenic region, and lncRNA genes are labeled purple; Table S8. Overall counts for each impact category (HIGH, MODERATE, and NEUTRAL) with the total number of homozygote alternate variants for 23 Bearded Collies. Variant files were annotated to the CanFam3.1 reference genome.

## Abbreviations

The following abbreviations are used in this manuscript:

LD	Linkage Disequilibrium
ROH	Runs of Homozygosity
SD	Standard Deviation
SNP	Single Nucleotide Polymorphism
WGS	Whole Genome Sequencing

## References

[1] Hedhammar Å.A., Malm S., Bonnett B. (2011). International and collaborative strategies to enhance genetic health in purebred dogs. Vet. J..

[2] Velie B.D., Wilson B.J., Arnott E.R., Early J.B., McGreevy P.D., Wade C.M. (2021). Inbreeding levels in an open-registry pedigreed dog breed: The Australian working kelpie. Vet. J..

[3] Farrell L.L., Schoenebeck J.J., Wiener P., Clements D.N., Summers K.M. (2015). The challenges of pedigree dog health: Approaches to combating inherited disease. Canine Genet. Epidemiol..

[4] Proschowsky H.F., Arendt M.L., Bonnett B.N., Bruun C.S., Czycholl I., Fredholm M., O’Neill D., Serpell J.A., Sandøe P. (2025). A New Future for Dog Breeding. Anim. Welf..

[5] Ku C.S., Naidoo N., Teo S.M., Pawitan Y. (2011). Regions of homozygosity and their impact on complex diseases and traits. Hum. Genet..

[6] Mandigers P.J., Ubbink G.J., Vanden Broek J., Bouw J. (1994). Relationship between litter size and other reproductive traits in the Dutch Kooiker dog. Vet. Q..

[7] Ubbink G.J., van de Broek J., Hazewinkel H.A., Rothuizen J. (1998). Cluster analysis of the genetic heterogeneity and disease distributions in purebred dog populations. Vet. Rec..

[8] Ubbink G.J., Knol B.W., Bouw J. (1992). The relationship between homozygosity and the occurrence of specific diseases in Bouvier Belge des Flandres dogs in the Netherlands: Inbreeding and disease in the bouvier dog. Vet. Q..

[9] Yordy J., Kraus C., Hayward J.J., White M.E., Shannon L.M., Creevy K.E., Promislow D.E.L., Boyko A.R. (2020). Body Size, Inbreeding, and Lifespan in Domestic Dogs. Conserv. Genet..

[10] Cecchi F., Vezzosi T., Branchi G., Barsotti G., Macchioni F. (2020). Inbreeding and health problems prevalence in a colony of guide dogs: A cohort of 40 Labrador Retrievers. Acta Agric. Scand. Sect. A—Anim. Sci..

[11] Jansson M., Laikre L. (2014). Recent breeding history of dog breeds in S weden: Modest rates of inbreeding, extensive loss of genetic diversity and lack of correlation between inbreeding and health. J. Anim. Breed. Genet..

[12] Mäki K., Groen A.F., Liinamo A.-E., Ojala M. (2001). Population structure, inbreeding trend and their association with hip and elbow dysplasia in dogs. Anim. Sci..

[13] Mooney J.A., Yohannes A., Lohmueller K.E. (2021). The impact of identity by descent on fitness and disease in dogs. Proc. Natl. Acad. Sci. USA.

[14] Wellmann R., Pfeiffer I. (2009). Pedigree analysis for conservation of genetic diversity and purging. Genet. Res..

[15] Chu E.T., Simpson M.J., Diehl K., Page R.L., Sams A.J., Boyko A.R. (2019). Inbreeding depression causes reduced fecundity in Golden Retrievers. Mamm. Genome.

[16] Leroy G., Phocas F., Hedan B., Verrier E., Rognon X. (2015). Inbreeding impact on litter size and survival in selected canine breeds. Vet. J..

[17] Halvoník A., Moravčíková N., Vostrý L., Vostra-Vydrova H., Mészáros G., Demir E., Chalupková M., Kasarda R. (2025). Heterozygosity-Rich Regions in Canine Genome: Can They Serve as Indicators of Balancing Selection?. Animals.

[18] Ács V., Kövér G., Farkas J., Bokor Á., Nagy I. (2020). Effects of Long-Term Selection in the Border Collie Dog Breed: Inbreeding Purge of Canine Hip and Elbow Dysplasia. Animals.

[19] Wellmann R. (2023). Selection index theory for populations under directional and stabilizing selection. Genet. Sel. Evol..

[20] Syvänen A.-C. (2001). Accessing genetic variation: Genotyping single nucleotide polymorphisms. Nat. Rev. Genet..

[21] Venter J.C., Adams M.D., Myers E.W., Li P.W., Mural R.J., Sutton G.G., Smith H.O., Yandell M., Evans C.A., Holt R.A. (2001). The Sequence of the Human Genome. Science.

[22] Karjalainen L., Ojala M. (1997). Generation intervals and inbreeding coefficients in the Finnish Hound and the Finnish Spitz. J. Anim. Breed. Genet..

[23] Leroy G. (2011). Genetic diversity, inbreeding and breeding practices in dogs: Results from pedigree analyses. Vet. J..

[24] Brouillette J.A., Andrew J.R., Venta P.J. (2000). Estimate of nucleotide diversity in dogs with a pool-and-sequence method. Mamm. Genome.

[25] Wade C.M. (2011). Inbreeding and genetic diversity in dogs: Results from DNA analysis. Vet. J..

[26] Rincon G., Tengvall K., Belanger J.M., Lagoutte L., Medrano J.F., André C., Thomas A., Lawley C.T., Hansen M.S., Lindblad-Toh K. (2011). Comparison of Buccal and Blood-Derived Canine DNA, Either Native or Whole Genome Amplified, for Array-Based Genome-Wide Association Studies. BMC Res. Notes.

[27] Gershony L.C., Belanger J.M., Hytönen M.K., Lohi H., Oberbauer A.M. (2019). Novel Locus Associated with Symmetrical Lupoid Onychodystrophy in the Bearded Collie. Genes.

[28] Dryad Digital Repository. https://datadryad.org/.

[29] Baker P.R., Baschal E.E., Fain P.R., Triolo T.M., Nanduri P., Siebert J.C., Armstrong T.K., Babu S.R., Rewers M.J., Gottlieb P.A. (2010). Haplotype Analysis Discriminates Genetic Risk for DR3-Associated Endocrine Autoimmunity and Helps Define Extreme Risk for Addison’s Disease. J. Clin. Endocrinol. Metab..

[30] Dahlgren S., Ziener M.L., Lingaas F. (2016). A genome-wide association study identifies a region strongly associated with symmetrical onychomadesis on chromosome 12 in dogs. Anim. Genet..

[31] Forsberg S.K.G., Kierczak M., Ljungvall I., Merveille A.-C., Gouni V., Wiberg M., Lundgren Willesen J., Hanås S., Lequarré A.-S., Mejer Sørensen L. (2015). The Shepherds’ Tale: A Genome-Wide Study across 9 Dog Breeds Implicates Two Loci in the Regulation of Fructosamine Serum Concentration in Belgian Shepherds. PLoS ONE.

[32] Kawakami T., Raghavan V., Ruhe A.L., Jensen M.K., Milano A., Nelson T.C., Boyko A.R. (2022). Early Onset Adult Deafness in the Rhodesian Ridgeback Dog Is Associated with an In-Frame Deletion in the EPS8L2 Gene. PLoS ONE.

[33] Olsson M., Tengvall K., Frankowiack M., Kierczak M., Bergvall K., Axelsson E., Tintle L., Marti E., Roosje P., Leeb T. (2015). Genome-Wide Analyses Suggest Mechanisms Involving Early B-Cell Development in Canine IgA Deficiency. PLoS ONE.

[34] Pilot M., Malewski T., Moura A.E., Grzybowski T., Oleński K., Ruść A., Kamiński S., Ruiz Fadel F., Mills D.S., Alagaili A.N. (2015). On the Origin of Mongrels: Evolutionary History of Free-Breeding Dogs in Eurasia. Proc. R. Soc. B.

[35] Slavney A.J., Kawakami T., Jensen M.K., Nelson T.C., Sams A.J., Boyko A.R. (2021). Five genetic variants explain over 70% of hair coat pheomelanin intensity variation in purebred and mixed breed domestic dogs. PLoS ONE.

[36] Stern J.A., Hsue W., Song K.-H., Ontiveros E.S., Luis Fuentes V., Stepien R.L. (2015). Severity of Mitral Valve Degeneration Is Associated with Chromosome 15 Loci in Whippet Dogs. PLoS ONE.

[37] Wolf Z.T., Brand H.A., Shaffer J.R., Leslie E.J., Arzi B., Willet C.E., Cox T.C., McHenry T., Narayan N., Feingold E. (2015). Genome-Wide Association Studies in Dogs and Humans Identify ADAMTS20 as a Risk Variant for Cleft Lip and Palate. PLoS Genet..

[38] Vaysse A., Ratnakumar A., Derrien T., Axelsson E., Rosengren Pielberg G., Sigurdsson S., Fall T., Seppälä E.H., Hansen M.S.T., Lawley C.T. (2011). Identification of Genomic Regions Associated with Phenotypic Variation between Dog Breeds Using Selection Mapping. PLoS Genet..

[39] Chang C.C., Chow C.C., Tellier L.C., Vattikuti S., Purcell S.M., Lee J.J. (2015). Second-generation PLINK: Rising to the challenge of larger and richer datasets. GigaScience.

[40] Gershony L.C., Belanger J.M., Hytönen M.K., Lohi H., Oberbauer A.M. (2021). Whole Genome Sequencing Reveals Multiple Linked Genetic Variants on Canine Chromosome 12 Associated with Risk for Symmetrical Lupoid Onychodystrophy (SLO) in the Bearded Collie. Genes.

[41] Auwera G., van der O’Connor B.D. (2020). Genomics in the Cloud: Using Docker, GATK, and WDL in Terra.

[42] Danecek P., Auton A., Abecasis G., Albers C.A., Banks E., DePristo M.A., Handsaker R.E., Lunter G., Marth G.T., Sherry S.T. (2011). The Variant Call Format and VCFtools. Bioinformatics.

[43] Jagannathan V., Drögemüller C., Leeb T., Dog Biomedical Variant Database Consortium (DBVDC) (2019). A comprehensive biomedical variant catalogue based on whole genome sequences of 582 dogs and eight wolves. Anim. Genet..

[44] DePristo M.A., Banks E., Poplin R., Garimella K.V., Maguire J.R., Hartl C., Philippakis A.A., Del Angel G., Rivas M.A., Hanna M. (2011). A Framework for Variation Discovery and Genotyping Using Next-Generation DNA Sequencing Data. Nat. Genet..

[45] Alemu S.W., Kadri N.K., Harland C., Faux P., Charlier C., Caballero A., Druet T. (2021). An Evaluation of Inbreeding Measures Using a Whole-Genome Sequenced Cattle Pedigree. Heredity.

[46] McQuillan R., Leutenegger A.-L., Abdel-Rahman R., Franklin C.S., Pericic M., Barac-Lauc L., Smolej-Narancic N., Janicijevic B., Polasek O., Tenesa A. (2008). Runs of Homozygosity in European Populations. Am. J. Hum. Genet..

[47] Sams A.J., Boyko A.R. (2019). Fine-Scale Resolution of Runs of Homozygosity Reveal Patterns of Inbreeding and Substantial Overlap with Recessive Disease Genotypes in Domestic Dogs. G3 Genes|Genomes|Genet..

[48] Ceballos F.C., Hazelhurst S., Ramsay M. (2018). Assessing runs of Homozygosity: A comparison of SNP Array and whole genome sequence low coverage data. BMC Genom..

[49] Meadows J.R.S., Kidd J.M., Wang G.-D., Parker H.G., Schall P.Z., Bianchi M., Christmas M.J., Bougiouri K., Buckley R.M., Hitte C. (2023). Genome Sequencing of 2000 Canids by the Dog10K Consortium Advances the Understanding of Demography, Genome Function and Architecture. Genome Biol..

[50] Meyermans R., Gorssen W., Buys N., Janssens S. (2020). How to study runs of homozygosity using PLINK? A guide for analyzing medium density SNP data in livestock and pet species. BMC Genom..

[51] Lobo D., López-Bao J.V., Godinho R. (2023). The population bottleneck of the Iberian wolf impacted genetic diversity but not admixture with domestic dogs: A temporal genomic approach. Mol. Ecol..

[52] Dillon M.N., Thomas R., Mousseau T.A., Betz J.A., Kleiman N.J., Reiskind M.O.B., Breen M. (2023). Population Dynamics and Genome-Wide Selection Scan for Dogs in Chernobyl. Canine Med. Genet..

[53] Gorssen W., Meyermans R., Janssens S., Buys N. (2021). A publicly available repository of ROH islands reveals signatures of selection in different livestock and pet species. Genet. Sel. Evol..

[54] Lencz T., Lambert C., DeRosse P., Burdick K.E., Morgan T.V., Kane J.M., Kucherlapati R., Malhotra A.K. (2007). Runs of Homozygosity Reveal Highly Penetrant Recessive Loci in Schizophrenia. Proc. Natl. Acad. Sci. USA.

[55] Mastrangelo S., Biscarini F., Tolone M., Auzino B., Ragatzu M., Spaterna A., Ciampolini R. (2018). Genomic Characterization of the Braque Français Type Pyrénées Dog and Relationship with Other Breeds. PLoS ONE.

[56] Ferenčaković M., Hamzić E., Gredler B., Solberg T.R., Klemetsdal G., Curik I., Sölkner J. (2013). Estimates of Autozygosity Derived from Runs of Homozygosity: Empirical Evidence from Selected Cattle Populations. J. Anim. Breed. Genet..

[57] Mortlock S.-A., Khatkar M.S., Williamson P. (2016). Comparative Analysis of Genome Diversity in Bullmastiff Dogs. PLoS ONE.

[58] Dreger D.L., Rimbault M., Davis B.W., Bhatnagar A., Parker H.G., Ostrander E.A. (2016). Whole genome sequence, SNP chips and pedigree structure: Building demographic profiles in domestic dog breeds to optimize genetic trait mapping. Dis. Models Mech..

[59] Biscarini F., Cozzi P., Gaspa G., Marras G. (2018). detectRUNS: An R Package to Detect Runs of Homozygosity and Heterozygosity in Diploid Genomes. https://cran.r-project.org/web/packages/detectRUNS/vignettes/detectRUNS.vignette.html.

[60] R Core Team (2023). R: A Language and Environment for Statistical Computing.

[61] Lowry R. (2024). VassarStats: Website for Statistical Computation.

[62] Perfilyeva A., Bespalova K., Bespalov S., Begmanova M., Kuzovleva Y., Vishnyakova O., Nazarenko I., Abylkassymova G., Perfilyeva Y., Plakhov K. (2023). Homozygosity Mapping in the Kazakh National Dog Breed Tazy. Sci. Rep..

[63] Rocha R.D.F.B., Garcia A.O., Otto P.I., Da Silva M.V.B., Martins M.F., Machado M.A., Panetto J.C.D.C., Guimarães S.E.F. (2023). Runs of Homozygosity and Signatures of Selection for Number of Oocytes and Embryos in the Gir Indicine Cattle. Mamm. Genome.

[64] Vasiliadis D., Metzger J., Distl O. (2020). Demographic assessment of the Dalmatian dog—Effective population size, linkage disequilibrium and inbreeding coefficients. Canine Genet. Epidemiol..

[65] Boyko A.R., Quignon P., Li L., Schoenebeck J.J., Degenhardt J.D., Lohmueller K.E., Zhao K., Brisbin A., Parker H.G., vonHoldt B.M. (2010). A Simple Genetic Architecture Underlies Morphological Variation in Dogs. PLoS Biol..

[66] Wickham H., Averick M., Bryan J., Chang W., McGowan L., François R., Grolemund G., Hayes A., Henry L., Hester J. (2019). Welcome to the Tidyverse. JOSS.

[67] Weir B.S., Cockerham C.C. (1984). Estimating F-Statistics for the analysis of population structure. Evolution.

[68] Wright S. (1965). The Interpretation of Population Structure by F-Statistics with Special Regard to Systems of Mating. Evolution.

[69] Ukawa H., Akiyama N., Yamamoto F., Ohashi K., Ishihara G., Matsumoto Y. (2024). Negative Selection on a *SOD1* Mutation Limits Canine Degenerative Myelopathy While Avoiding Inbreeding. Genome Biol. Evol..

[70] Cagan A., Blass T. (2016). Identification of genomic variants putatively targeted by selection during dog domestication. BMC Evol. Biol..

[71] Wickham H. (2016). ggplot2: Elegant Graphics for Data Analysis.

[72] Cingolani P., Platts A., Wang L.L., Coon M., Nguyen T., Wang L., Land S.J., Lu X., Ruden D.M. (2012). A Program for Annotating and Predicting the Effects of Single Nucleotide Polymorphisms, SnpEff: SNPs in the Genome of Drosophila Melanogaster Strain W1118; Iso-2; Iso-3. Fly (Austin).

[73] Cingolani P., Patel V.M., Coon M., Nguyen T., Land S.J., Ruden D.M., Lu X. (2012). Using Drosophila Melanogaster as a Model for Genotoxic Chemical Mutational Studies with a New Program, SnpSift. Front. Gene..

[74] Danecek P., Bonfield J.K., Liddle J., Marshall J., Ohan V., Pollard M.O., Whitwham A., Keane T., McCarthy S.A., Davies R.M. (2021). Twelve Years of SAMtools and BCFtools. GigaScience.

[75] RStudio Team (2024). RStudio: Integrated Development for R.

[76] Lyon M.S., Andrews S.J., Elsworth B., Gaunt T.R., Hemani G., Marcora E. (2021). The variant call format provides efficient and robust storage of GWAS summary statistics. Genome Biol..

[77] Bannasch D., Famula T., Donner J., Anderson H., Honkanen L., Batcher K., Safra N., Thomasy S., Rebhun R. (2021). The Effect of Inbreeding, Body Size and Morphology on Health in Dog Breeds. Canine Genet. Epidemiol..

[78] Donner J., Freyer J., Davison S., Anderson H., Blades M., Honkanen L., Inman L., Brookhart-Knox C.A., Louviere A., Forman O.P. (2023). Genetic Prevalence and Clinical Relevance of Canine Mendelian Disease Variants in over One Million Dogs. PLoS Genet..

[79] Kania-Gierdziewicz J., Gierdziewicz M., Budzyński B. (2015). Genetic Structure Analysis of Tatra Shepherd Dog Population From Tatra Mountain Region. Ann. Anim. Sci..

[80] Letko A., Hédan B., Snell A., Harris A.C., Jagannathan V., Andersson G., Holst B.S., Ostrander E.A., Quignon P., André C. (2023). Genomic Diversity and Runs of Homozygosity in Bernese Mountain Dogs. Genes.

[81] Letko A., Minor K.M., Jagannathan V., Seefried F.R., Mickelson J.R., Oliehoek P., Drögemüller C. (2020). Genomic Diversity and Population Structure of the Leonberger Dog Breed. Genet. Sel. Evol..

[82] Soh P.X.Y., Hsu W.T., Khatkar M.S., Williamson P. (2021). Evaluation of genetic diversity and management of disease in Border Collie dogs. Sci. Rep..

[83] Wade C.M., Nuttall R., Liu S. (2023). Comprehensive analysis of geographic and breed-purpose influences on genetic diversity and inherited disease risk in the Doberman dog breed. Canine Med. Genet..

[84] Schmidt T.L., Jasper M., Weeks A.R., Hoffmann A.A. (2021). Unbiased population heterozygosity estimates from genome-wide sequence data. Methods Ecol. Evol..

[85] Lindblad-Toh K., Wade C.M., Mikkelsen T.S., Karlsson E.K., Jaffe D.B., Kamal M., Clamp M., Chang J.L., Kulbokas E.J., Zody M.C. (2005). Genome Sequence, Comparative Analysis and Haplotype Structure of the Domestic Dog. Nature.

[86] Lindblad-Toh K. (2020). What animals can teach us about evolution, the human genome, and human disease. Upsala J. Med. Sci..

[87] Kuderna L.F.K., Ulirsch J.C., Rashid S., Ameen M., Sundaram L., Hickey G., Cox A.J., Gao H., Kumar A., Aguet F. (2024). Identification of Constrained Sequence Elements across 239 Primate Genomes. Nature.

[88] Liu H., Sørensen A.C., Meuwissen T.H., Berg P. (2014). Allele frequency changes due to hitch-hiking in genomic selection programs. Genet. Sel. Evol..

[89] Metzger J., Karwath M., Tonda R., Beltran S., Águeda L., Gut M., Gut I.G., Distl O. (2015). Runs of Homozygosity Reveal Signatures of Positive Selection for Reproduction Traits in Breed and Non-Breed Horses. BMC Genom..

[90] Sumreddee P., Toghiani S., Hay E.H., Roberts A., Agrrey S.E., Rekaya R. (2019). Inbreeding depression in line 1 Hereford cattle population using pedigree and genomic information1. J. Anim. Sci..

[91] Nosrati M., Asadollahpour Nanaei H., Javanmard A., Esmailizadeh A. (2021). The pattern of runs of homozygosity and genomic inbreeding in world-wide sheep populations. Genomics.

[92] Boccardo A., Marelli S.P., Pravettoni D., Bagnato A., Busca G.A., Strillacci M.G. (2020). The German Shorthair Pointer Dog Breed (Canis lupus familiaris): Genomic Inbreeding and Variability. Animals.

[93] Sweetalana Nataneli S., Huang S., Mooney J.A., Szpiech Z.A. (2024). Genotypic and phenotypic consequences of domestication in dogs. bioRxiv.

[94] Subramanian S., Kumar M. (2024). The Association between the Abundance of Homozygous Deleterious Variants and the Morbidity of Dog Breeds. Biology.

[95] McMillan K.M., Bielby J., Williams C.L., Upjohn M.M., Casey R.A., Christley R.M. (2024). Longevity of companion dog breeds: Those at risk from early death. Sci. Rep..

[96] Marsden C.D., Ortega-Del Vecchyo D., O’Brien D.P., Taylor J.F., Ramirez O., Vilà C., Marques-Bonet T., Schnabel R.D., Wayne R.K., Lohmueller K.E. (2016). Bottlenecks and Selective Sweeps during Domestication Have Increased Deleterious Genetic Variation in Dogs. Proc. Natl. Acad. Sci. USA.

[97] Pemberton T.J., Absher D., Feldman M.W., Myers R.M., Rosenberg N.A., Li J.Z. (2012). Genomic Patterns of Homozygosity in Worldwide Human Populations. Am. J. Hum. Genet..

[98] Edmands S. (2007). Between a rock and a hard place: Evaluating the relative risks of inbreeding and outbreeding for conservation and management. Mol. Ecol..

[99] Urfer S.R., Kaeberlein M., Promislow D.E.L., Creevy K.E. (2020). Lifespan of companion dogs seen in three independent primary care veterinary clinics in the United States. Canine Genet. Epidemiol..

[100] Kettunen A., Daverdin M., Helfjord T., Berg P. (2017). Cross-Breeding Is Inevitable to Conserve the Highly Inbred Population of Puffin Hunter: The Norwegian Lundehund. PLoS ONE.

[101] Schoenebeck J.J., Ostrander E.A. (2014). Insights into Morphology and Disease from the Dog Genome Project. Annu. Rev. Cell Dev. Biol..

[102] Browning S.R., Browning B.L. (2012). Identity by Descent Between Distant Relatives: Detection and Applications. Annu. Rev. Genet..

[103] LeRoy J., Stuart R., Moorhead Mahigian C. (1995). Bearded Collie Club of America 1994 Yearbook: Silver Anniversary Commemorative Edition.

[104] The UK Kennel Club (2025). Bearded Collie Breed. Bearded Collie About This Breed. https://www.thekennelclub.org.uk/search/breeds-a-to-z/breeds/pastoral/bearded-collie/#:~:text=In%201955%20a%20new%20Bearded,Images%20The%20Pastoral%20group%20Colours.

[105] Cadieu E., Neff M.W., Quignon P., Walsh K., Chase K., Parker H.G., VonHoldt B.M., Rhue A., Boyko A., Byers A. (2009). Coat Variation in the Domestic Dog Is Governed by Variants in Three Genes. Science.

[106] Schlamp F., Van Der Made J., Stambler R., Chesebrough L., Boyko A.R., Messer P.W. (2016). Evaluating the performance of selection scans to detect selective sweeps in domestic dogs. Mol. Ecol..

[107] American Kennel Club (1978). AKC Bearded Collie Standard. https://images.akc.org/pdf/breeds/standards/BeardedCollie.pdf.

[108] Lai J.-J., Cruz F.M., Rock K.L. (2020). Immune Sensing of Cell Death through Recognition of Histone Sequences by C-Type Lectin-Receptor-2d Causes Inflammation and Tissue Injury. Immunity.

[109] Di Donato N., Jean Y.Y., Maga A.M., Krewson B.D., Shupp A.B., Avrutsky M.I., Roy A., Collins S., Olds C., Willert R.A. (2016). Mutations in CRADD Result in Reduced Caspase-2-Mediated Neuronal Apoptosis and Cause Megalencephaly with a Rare Lissencephaly Variant. Am. J. Hum. Genet..

[110] Akey J.M., Ruhe A.L., Akey D.T., Wong A.K., Connelly C.F., Madeoy J., Nicholas T.J., Neff M.W. (2010). Tracking Footprints of Artificial Selection in the Dog Genome. Proc. Natl. Acad. Sci. USA.

[111] Chen W., Han Y., Chen Y., Liu X., Liang H., Wang C., Khan M.Z. (2025). Potential Candidate Genes Associated with Litter Size in Goats: A Review. Animals.

[112] Zhang X.D., Zhu H.Y., Zhou J., Wang N., Zhou N., Huang L., Wu T., Feng Y.F., Ding Y.Y., Yin Z.J. (2015). Relationship between Polymorphisms in Exon 10 of FSHR Gene and Litter Size in Swine. Genet. Mol. Res..

[113] Tao L., He X., Wang F., Zhong Y., Pan L., Wang X., Gan S., Di R., Chu M. (2020). Luzhong Mutton Sheep: Inbreeding and Selection Signatures. J. Anim. Sci. Technol..

[114] Guo Y., Liang J., Lv C., Wang Y., Wu G., Ding X., Quan G. (2022). Sequencing Reveals Population Structure and Selection Signatures for Reproductive Traits in Yunnan Semi-Fine Wool Sheep (Ovis Aries). Front. Genet..

[115] Carneiro M., Piorno V., Rubin C.-J., Alves J.M., Ferrand N., Alves P.C., Andersson L. (2015). Candidate Genes Underlying Heritable Differences in Reproductive Seasonality between Wild and Domestic Rabbits. Anim. Genet..

[116] Rowell J.L., Rybaczyk L.A., Fenger J.M., Kosarek C.E., Chun R., McNiel E.A., Valli V.E., Alvarez C.E., Kisseberth W.C. (2011). Abstract 4825: Common Genetic Pathways Are Involved in Canine Diffuse Large B Cell Lymphoma Relapse and Human Diffuse Large B Cell Lymphoma Lympomagenesis. Cancer Res..

[117] Aloni R., Olender T., Lancet D. (2006). Ancient genomic architecture for mammalian olfactory receptor clusters. Genome Biol..

[118] Bougiouri K., Aninta S.G., Charlton S., Harris A., Carmagnini A., Piličiauskienė G., Feuerborn T.R., Scarsbrook L., Tabadda K., Blaževičius P. (2024). Imputation of ancient canid genomes reveals inbreeding history over the past 10,000 years. bioRxiv.

[119] Batcher K., Dickinson P., Maciejczyk K., Brzeski K., Rasouliha S.H., Letko A., Drögemüller C., Leeb T., Bannasch D. (2020). Multiple FGF4 Retrocopies Recently Derived within Canids. Genes.

[120] Cheetham S.W., Faulkner G.J., Dinger M.E. (2020). Overcoming challenges and dogmas to understand the functions of pseudogenes. Nat. Rev. Genet..

[121] Mabunda R.S., Makgahlela M.L., Nephawe K.A., Mtileni B. (2022). Evaluation of Genetic Diversity in Dog Breeds Using Pedigree and Molecular Analysis: A Review. Diversity.

[122] Bosse M., Megens H., Derks M.F.L., De Cara Á.M.R., Groenen M.A.M. (2019). Deleterious alleles in the context of domestication, inbreeding, and selection. Evol. Appl..

[123] Donner J., Anderson H., Davison S., Hughes A.M., Bouirmane J., Lindqvist J., Lytle K.M., Ganesan B., Ottka C., Ruotanen P. (2018). Frequency and Distribution of 152 Genetic Disease Variants in over 100,000 Mixed Breed and Purebred Dogs. PLoS Genet..

[124] Bryson G.T., O’Neill D.G., Brand C.L., Belshaw Z., Packer R.M.A. (2024). The doodle dilemma: How the physical health of ‘Designer-crossbreed’ Cockapoo, Labradoodle and Cavapoo dogs’ compares to their purebred progenitor breeds. PLoS ONE.

