# Supplementary material for: Measures of Homozygosity and Relationship to Genetic Diversity in the Bearded Collie Breed
Source: Genes (Basel). 2025 Mar 27;16(4):378. doi: 10.3390/genes16040378 (PMC12026756; doi:10.3390/genes16040378)
Supplement: Supplementary file 1 [file genes-16-00378-s001.zip › FigureS1.pdf]

CFA1

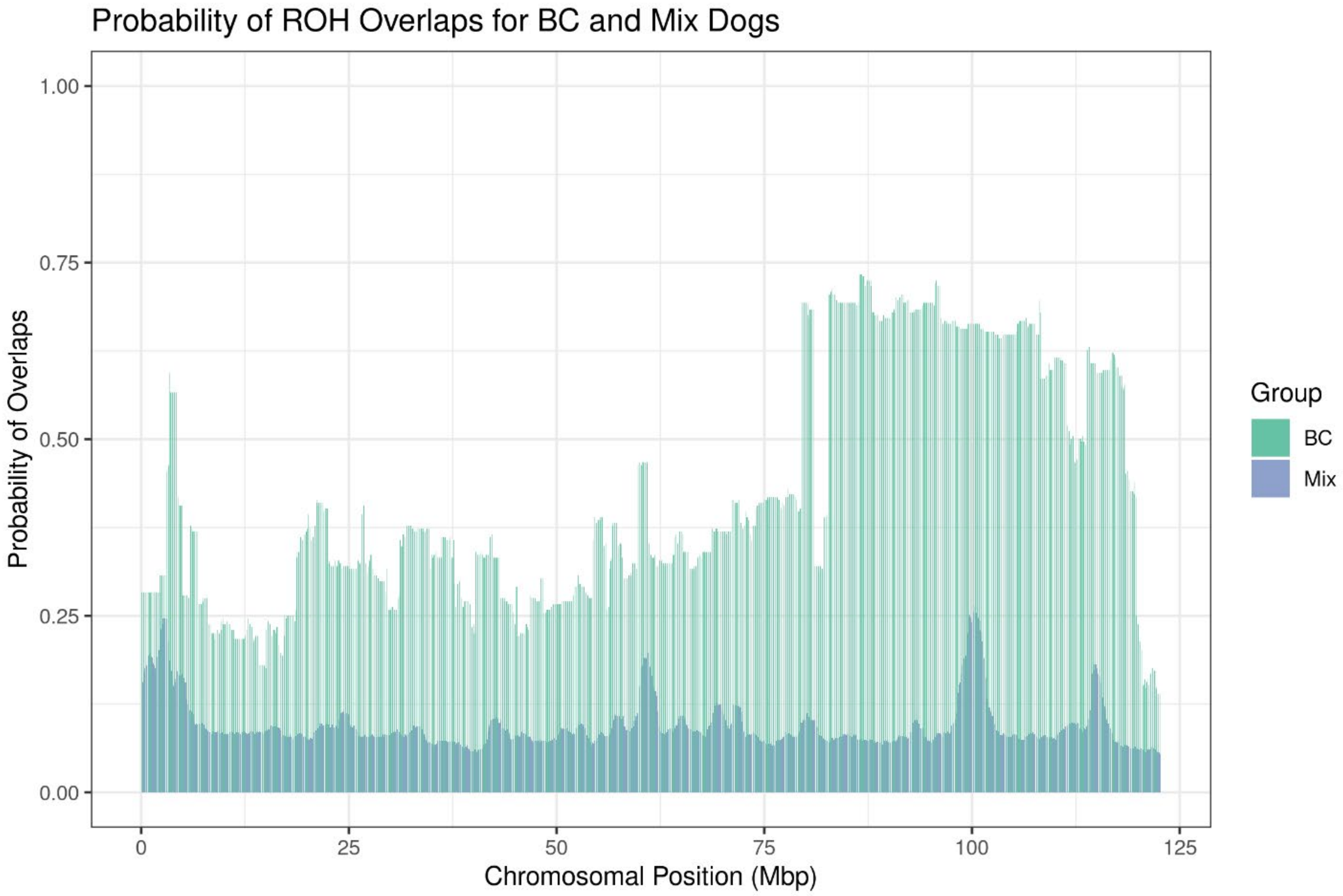
Probability of ROH Overlaps for BC and Mix Dogs
1.00
0.75
0.50
0.25
0.00
Probability of Overlaps
0
25
50
75
100
125
Chromosomal Position (Mbp)
Group
BC
Mix

CFA2

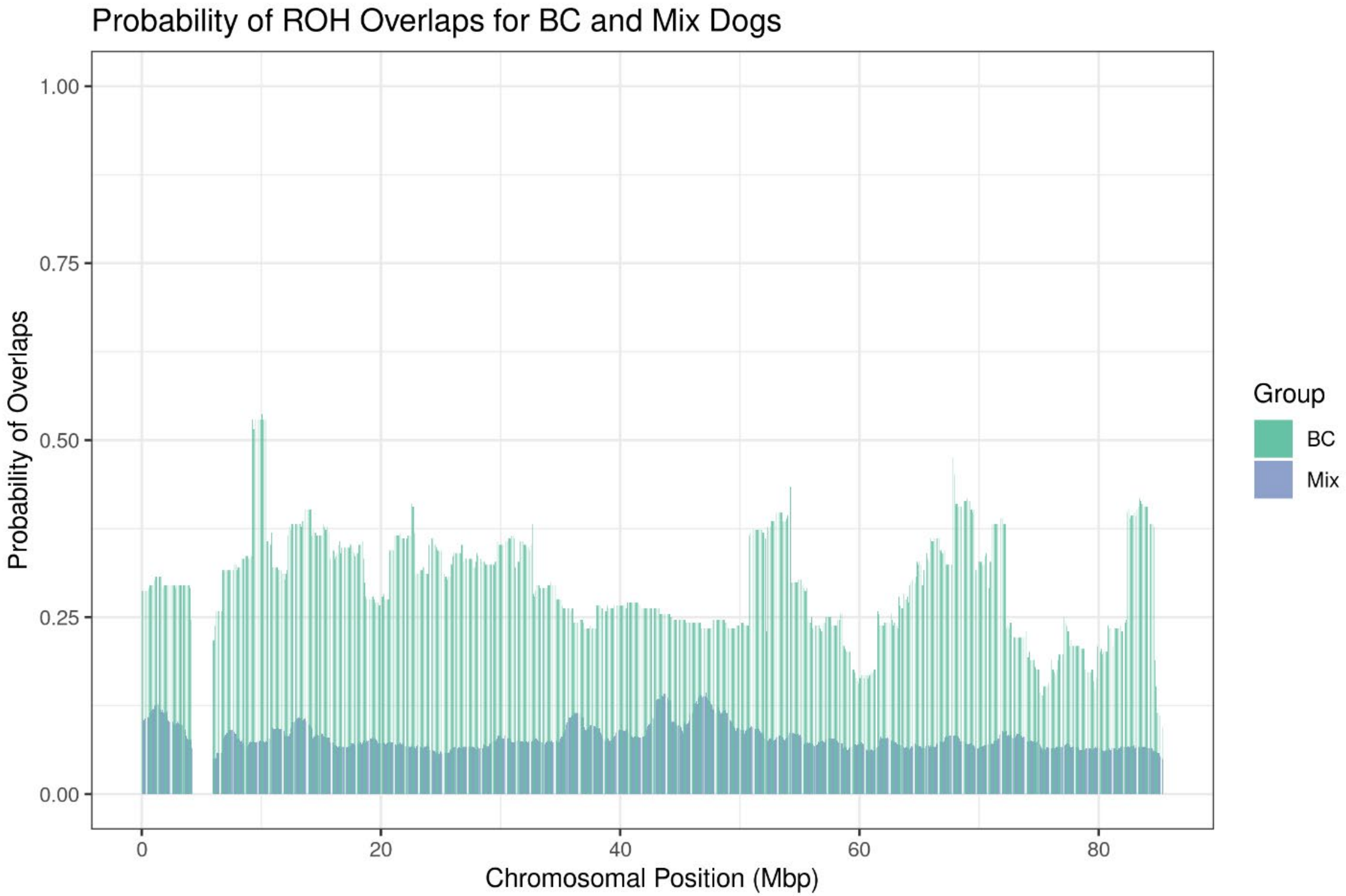
Probability of ROH Overlaps for BC and Mix Dogs
1.00
0.75
0.50
0.25
0.00
Probability of Overlaps
0
20
40
60
80
Chromosomal Position (Mbp)
Group
BC
Mix

CFA3

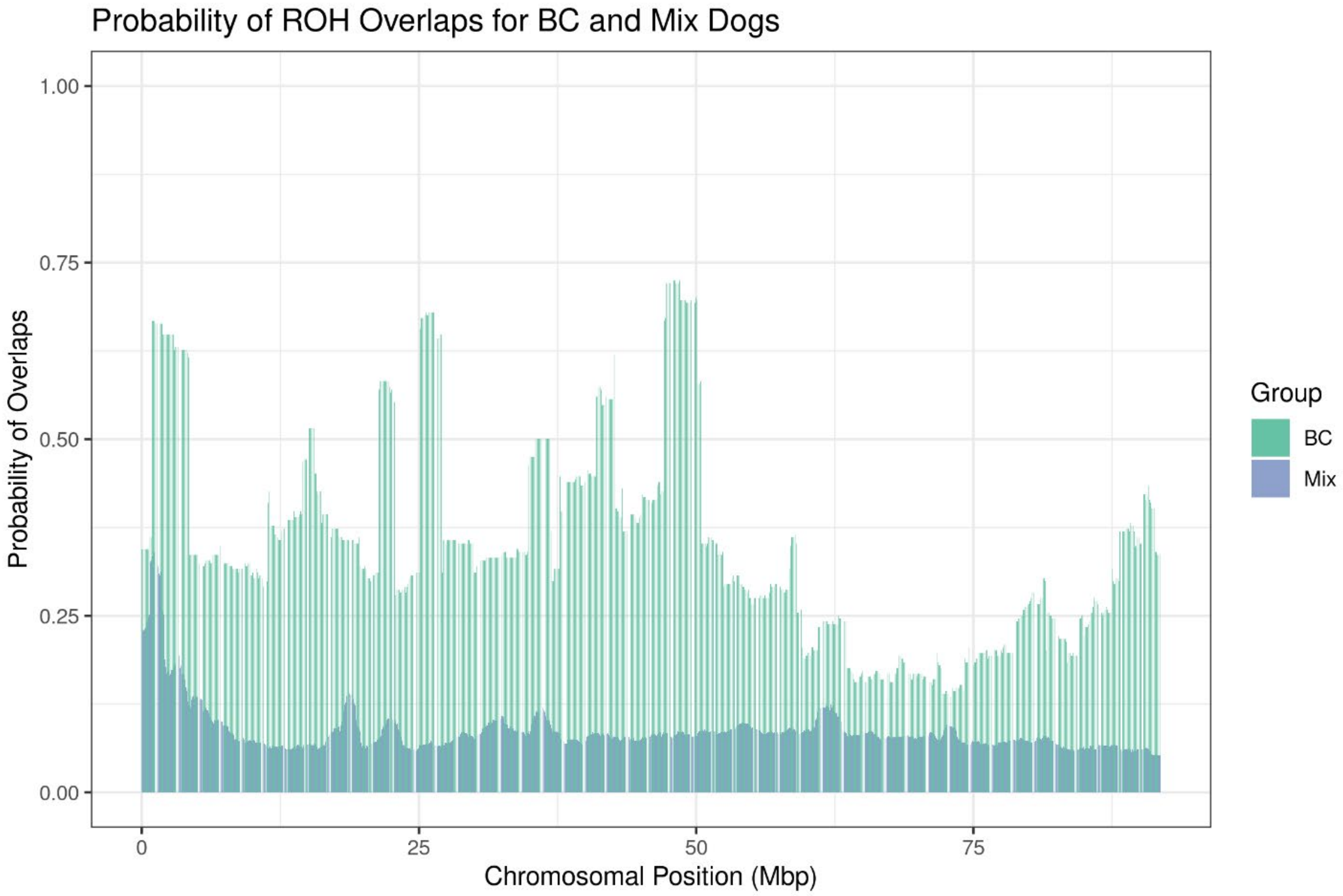

Probability of ROH Overlaps for BC and Mix Dogs
1.00
0.75
0.50
0.25
0.00
Probability of Overlaps
0
25
50
75
Chromosomal Position (Mbp)
Group
BC
Mix

CFA4

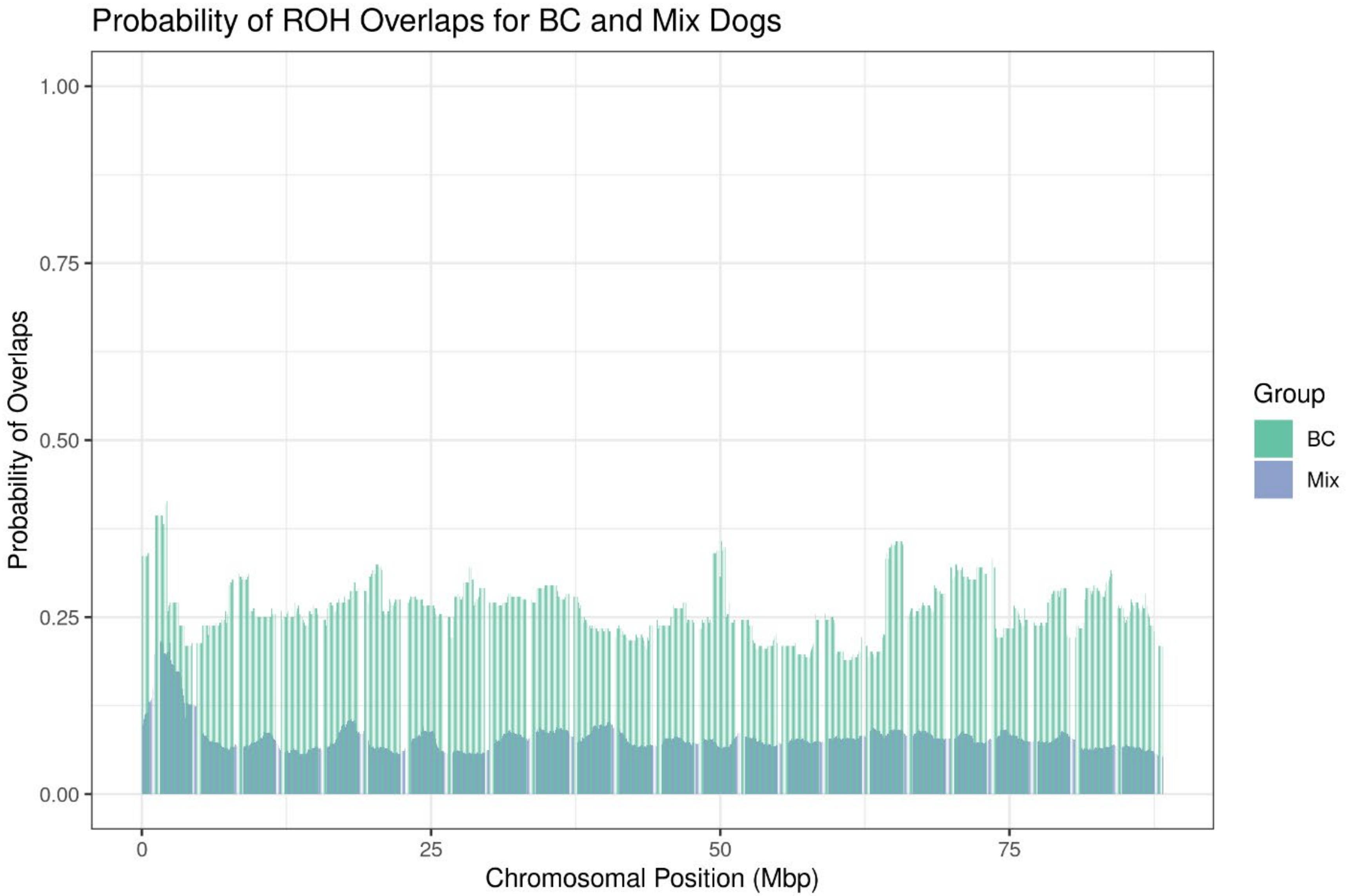

Probability of ROH Overlaps for BC and Mix Dogs
1.00
0.75
0.50
0.25
0.00
Probability of Overlaps
0
25
50
75
Chromosomal Position (Mbp)
Group
BC
Mix

CFA5

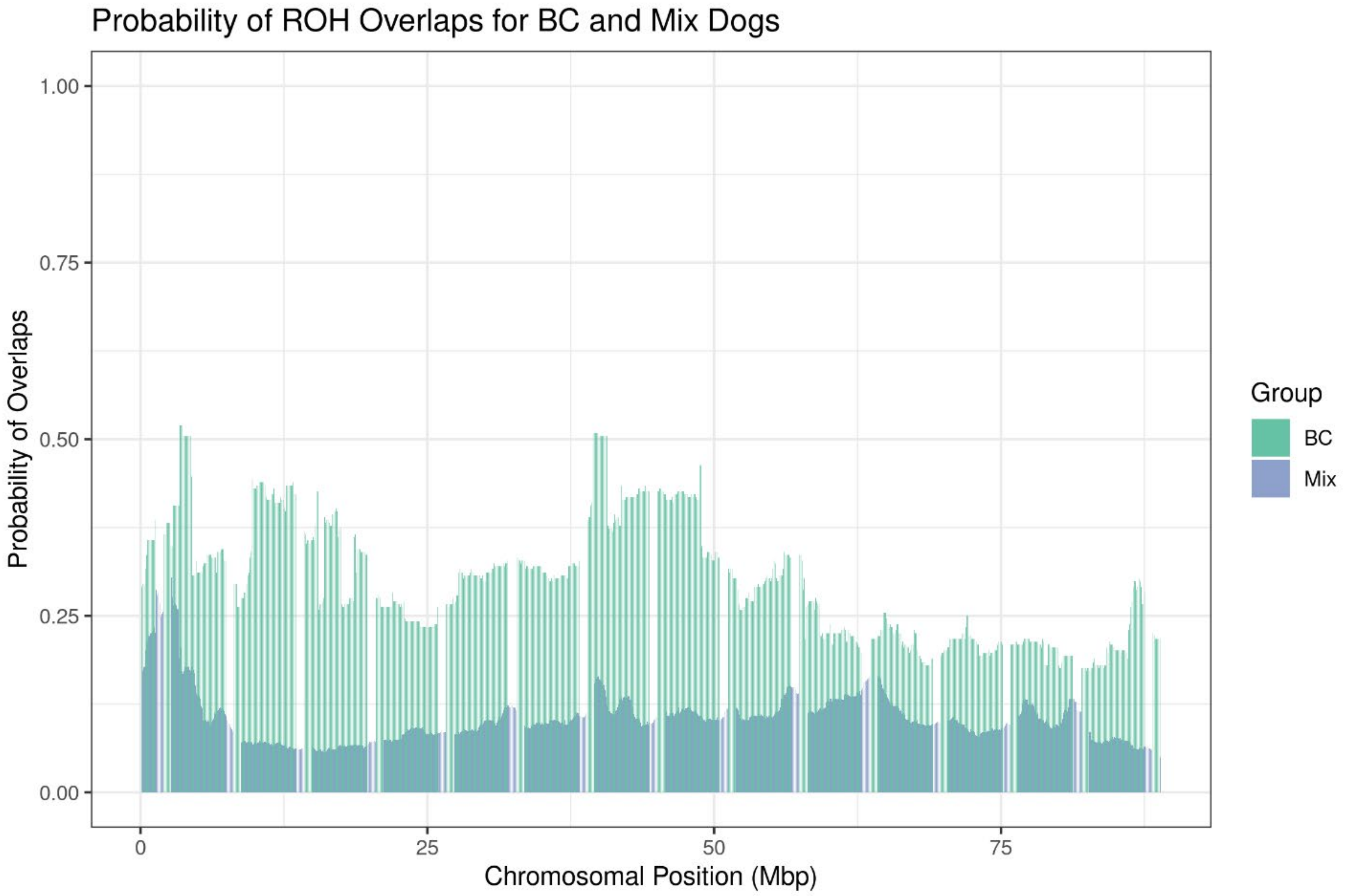
Probability of ROH Overlaps for BC and Mix Dogs
Probability of Overlaps
1.00
0.75
0.50
0.25
0.00
0
25
50
75
Chromosomal Position (Mbp)
Group
BC
Mix

CFA6

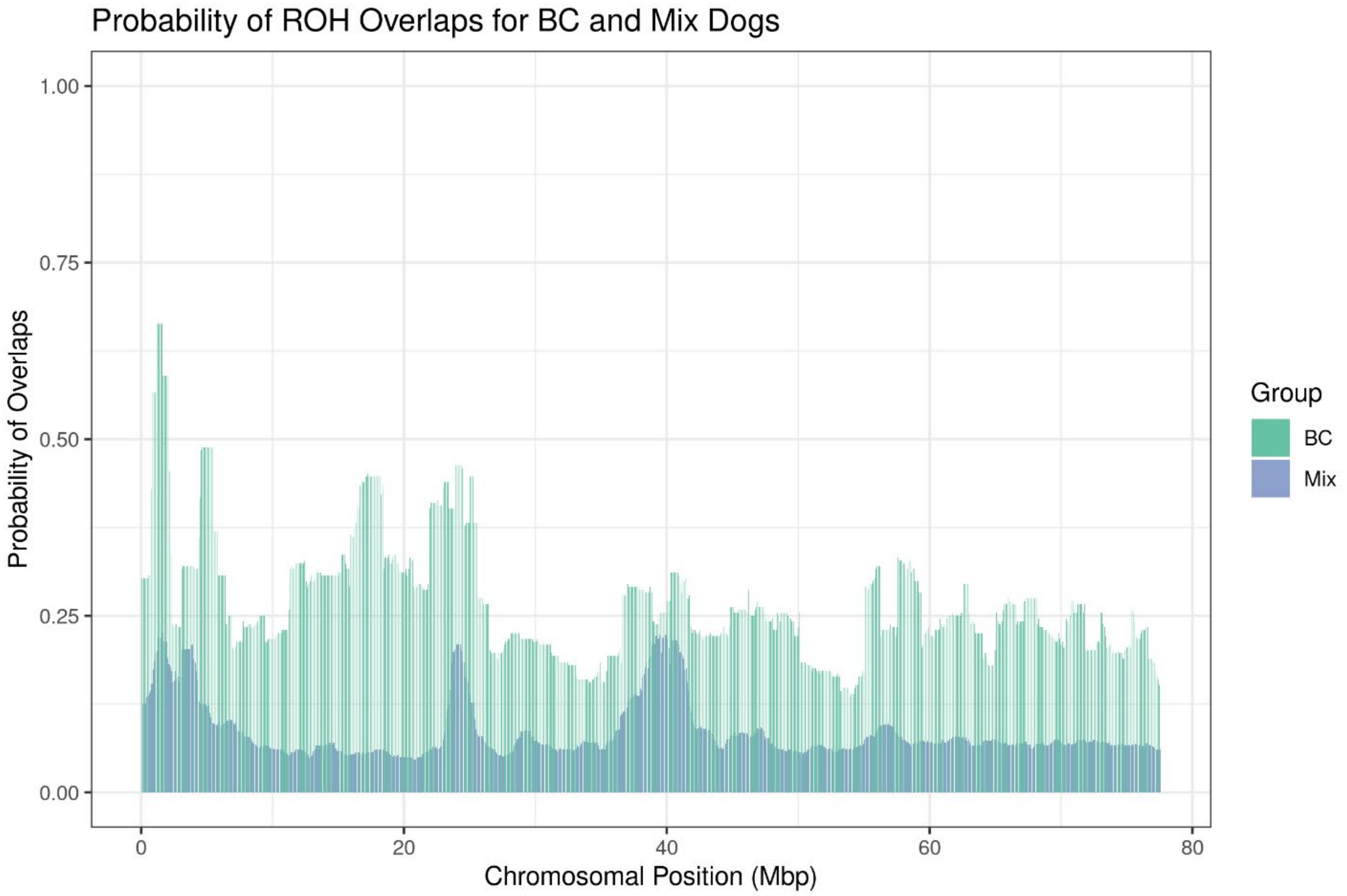

Probability of ROH Overlaps for BC and Mix Dogs
Probability of Overlaps
1.00
0.75
0.50
0.25
0.00
0
20
40
60
80
Chromosomal Position (Mbp)
Group
BC
Mix

CFA7

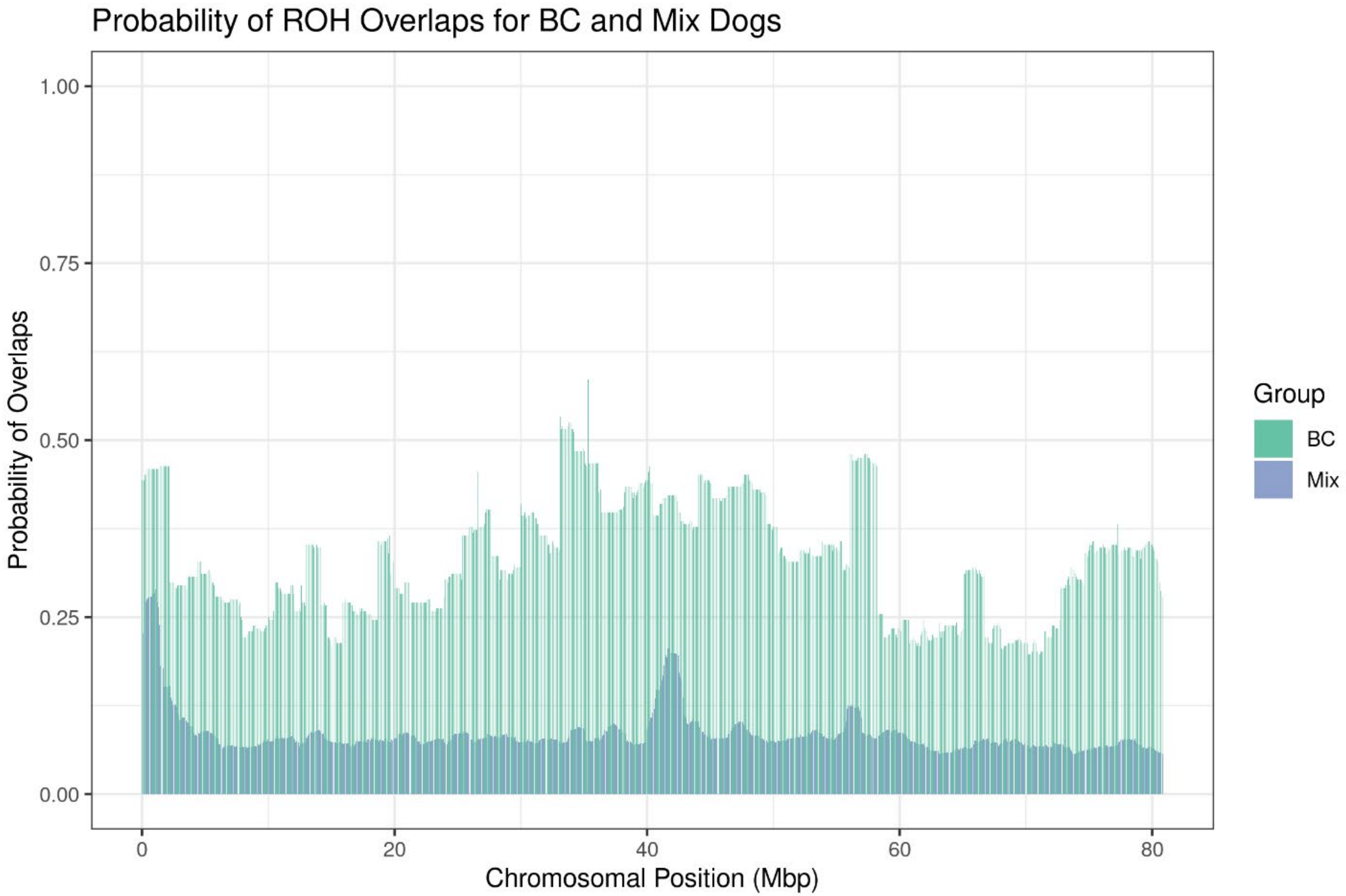
Probability of ROH Overlaps for BC and Mix Dogs
Probability of Overlaps
1.00
0.75
0.50
0.25
0.00
0
20
40
60
80
Chromosomal Position (Mbp)
Group
BC
Mix

CFA8

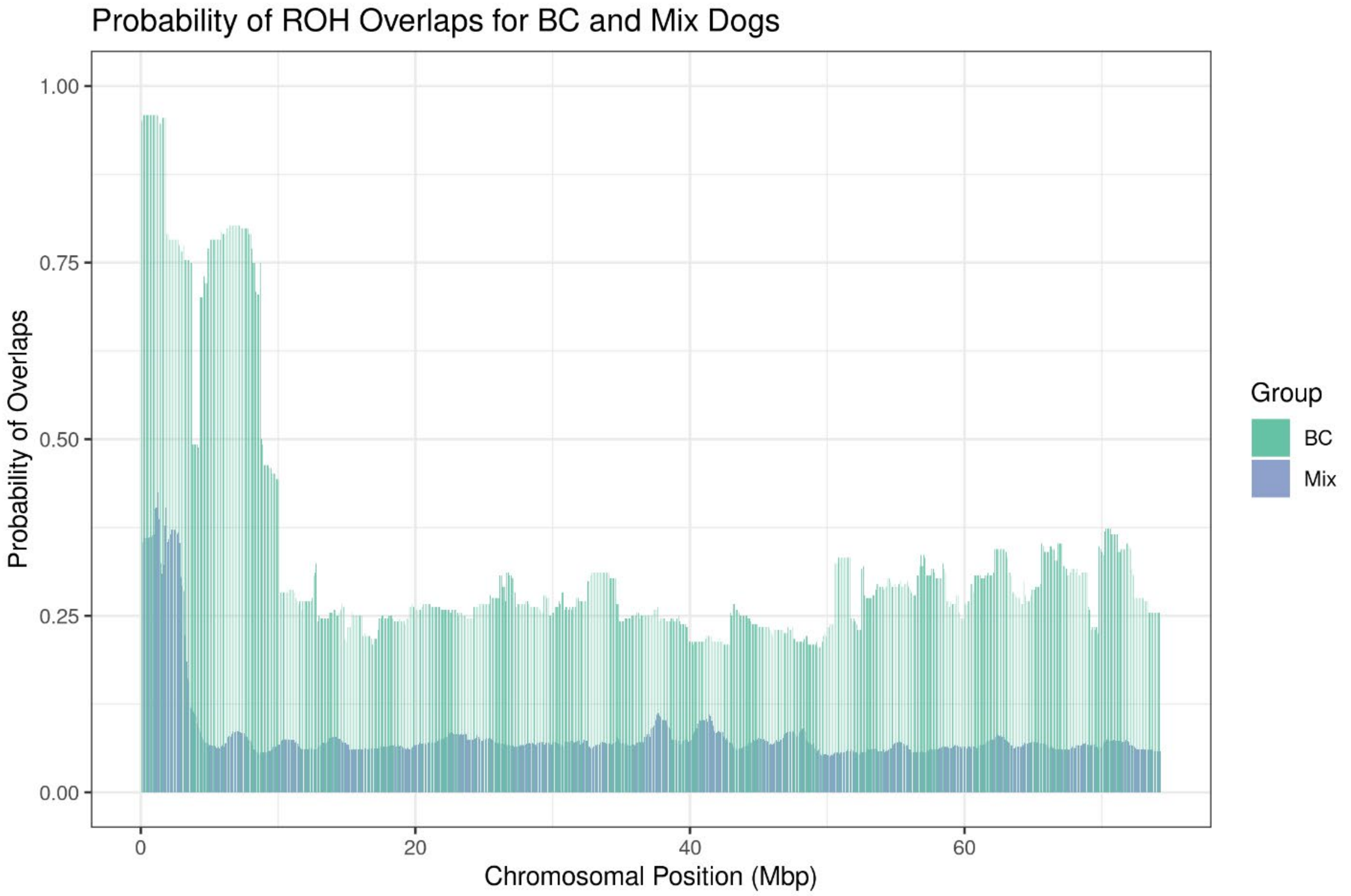

Probability of ROH Overlaps for BC and Mix Dogs
1.00
0.75
0.50
0.25
0.00
Probability of Overlaps
0
20
40
60
Chromosomal Position (Mbp)
Group
BC
Mix

CFA9

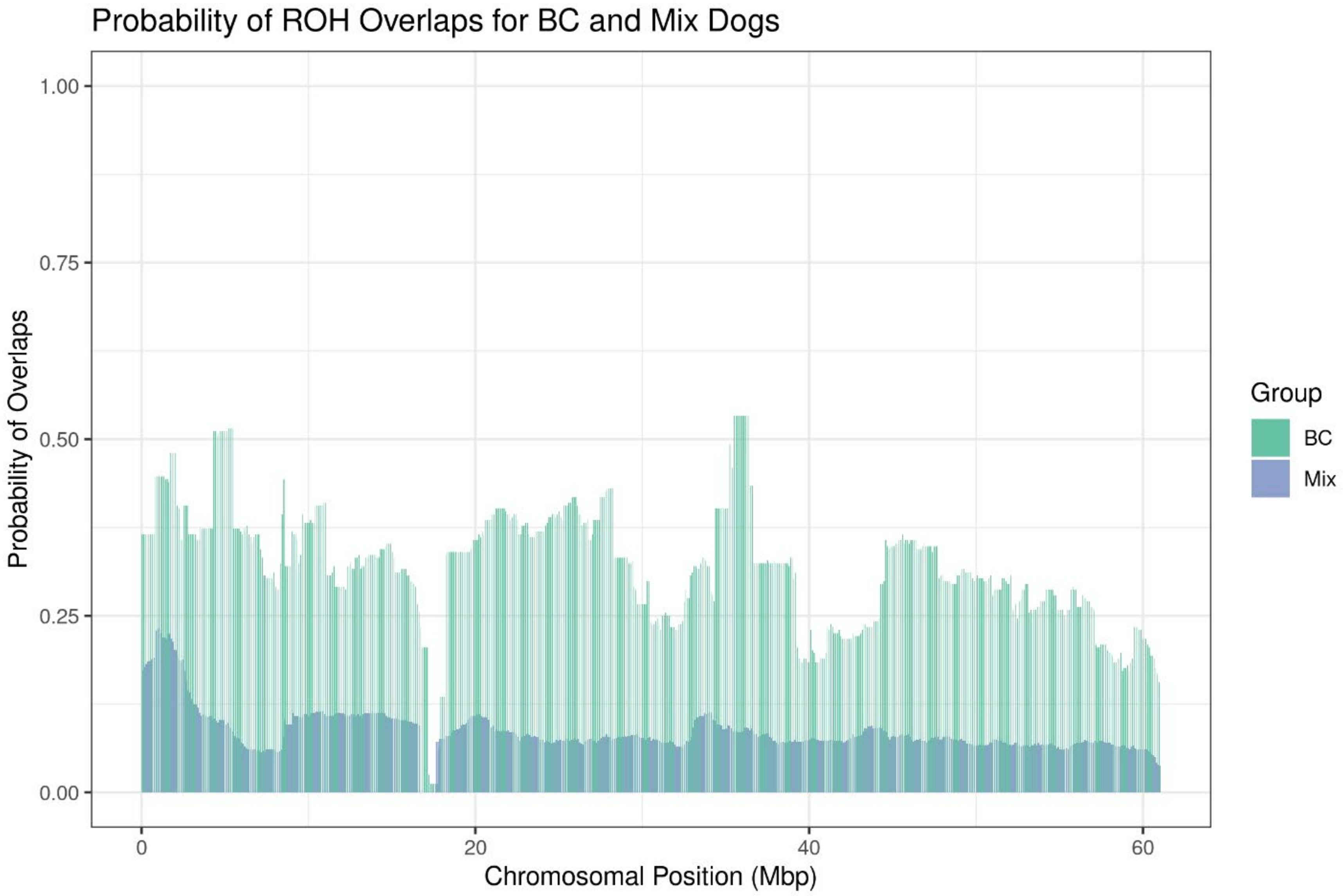

Probability of ROH Overlaps for BC and Mix Dogs
Probability of Overlaps
1.00
0.75
0.50
0.25
0.00
0
20
40
60
Chromosomal Position (Mbp)
Group
BC
Mix

CFA10

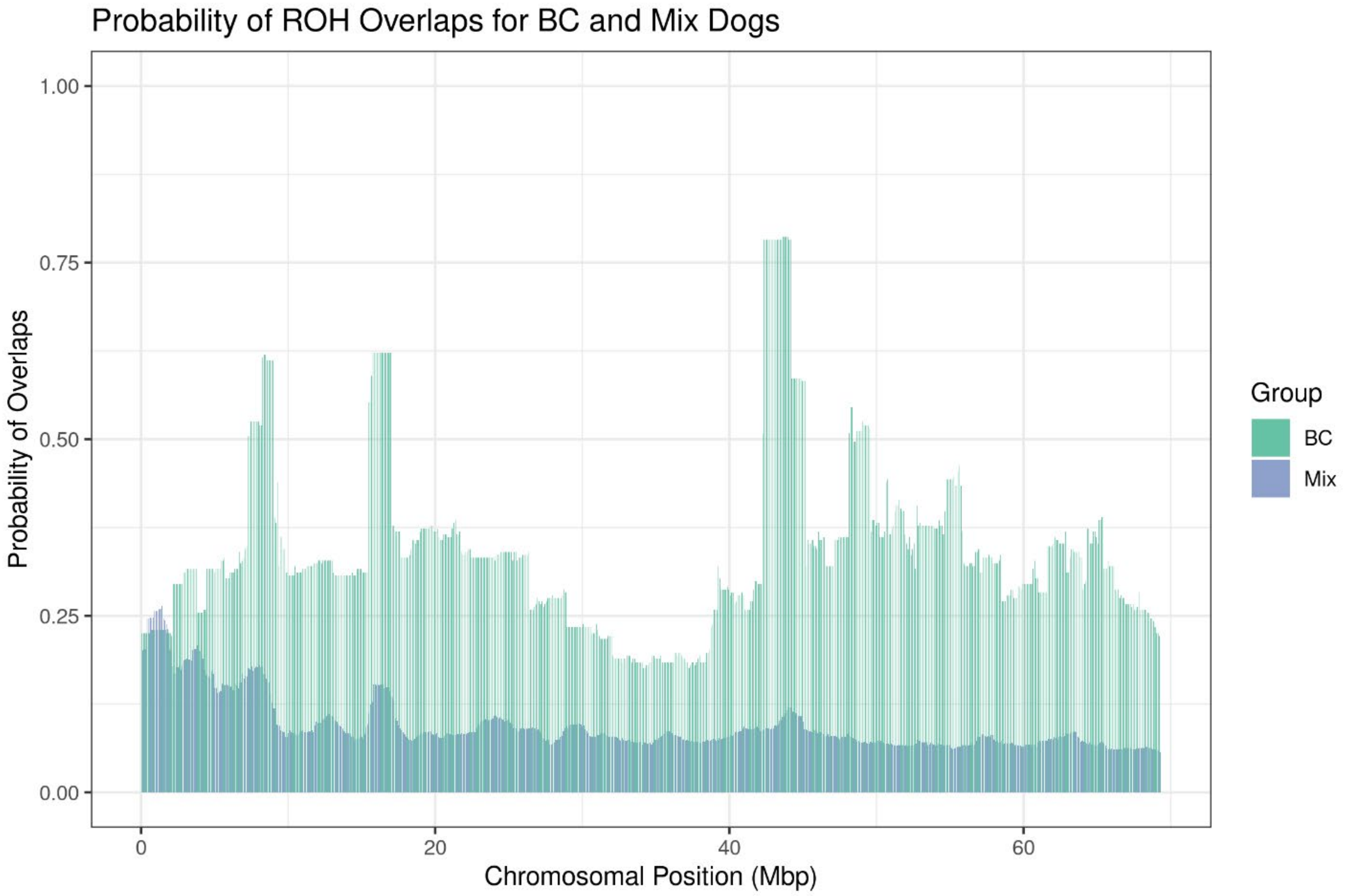

Probability of ROH Overlaps for BC and Mix Dogs
Probability of Overlaps
1.00
0.75
0.50
0.25
0.00
0
20
40
60
Chromosomal Position (Mbp)
Group
BC
Mix

CFA11

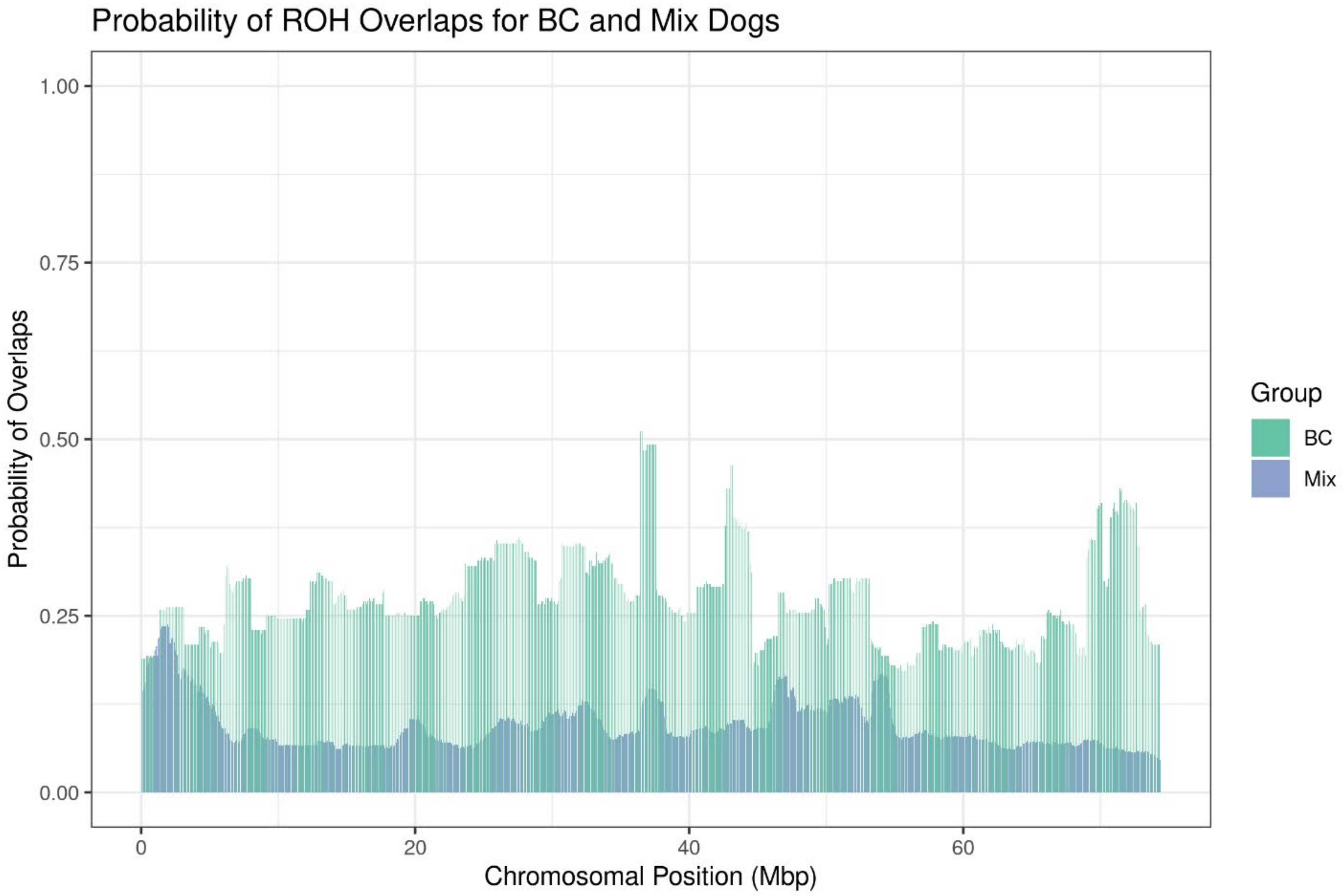
Probability of ROH Overlaps for BC and Mix Dogs
1.00
0.75
0.50
0.25
0.00
Probability of Overlaps
0
20
40
60
Chromosomal Position (Mbp)
Group
BC
Mix

CFA12

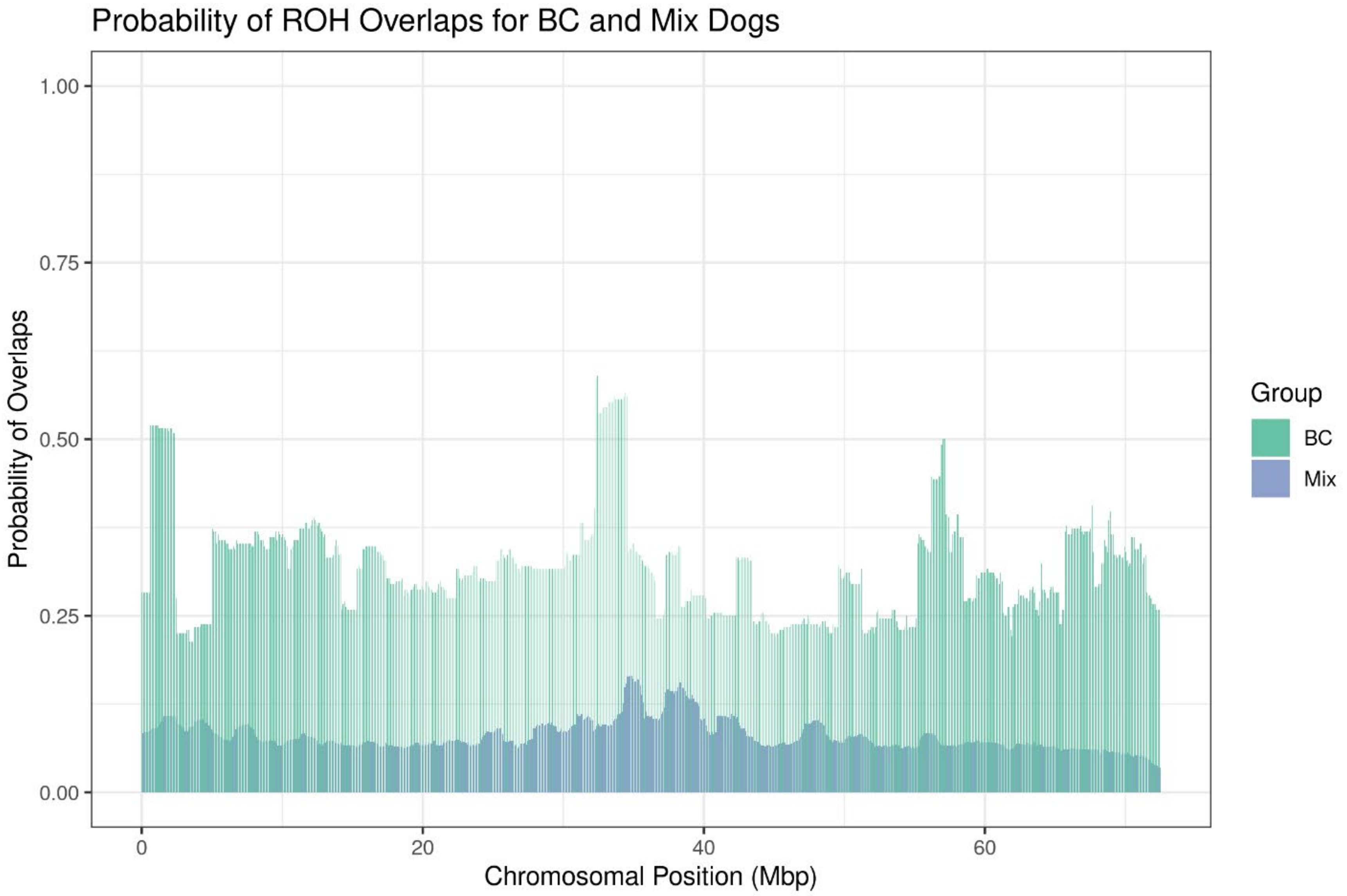
Probability of ROH Overlaps for BC and Mix Dogs
Probability of Overlaps
1.00
0.75
0.50
0.25
0.00
0
20
40
60
Chromosomal Position (Mbp)
Group
BC
Mix

CFA13

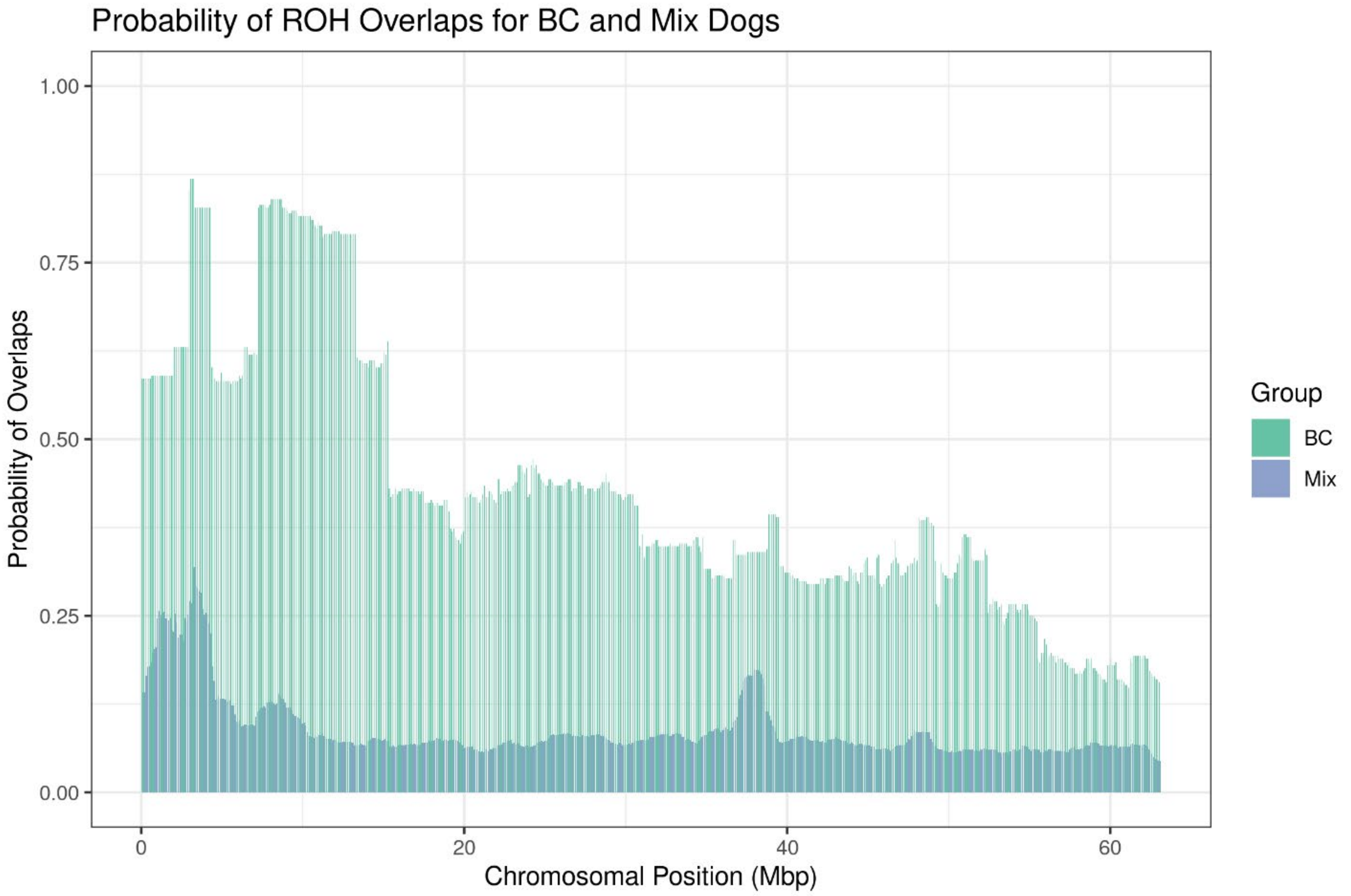

Probability of ROH Overlaps for BC and Mix Dogs
Probability of Overlaps
1.00
0.75
0.50
0.25
0.00
0
20
40
60
Chromosomal Position (Mbp)
Group
BC
Mix

CFA14

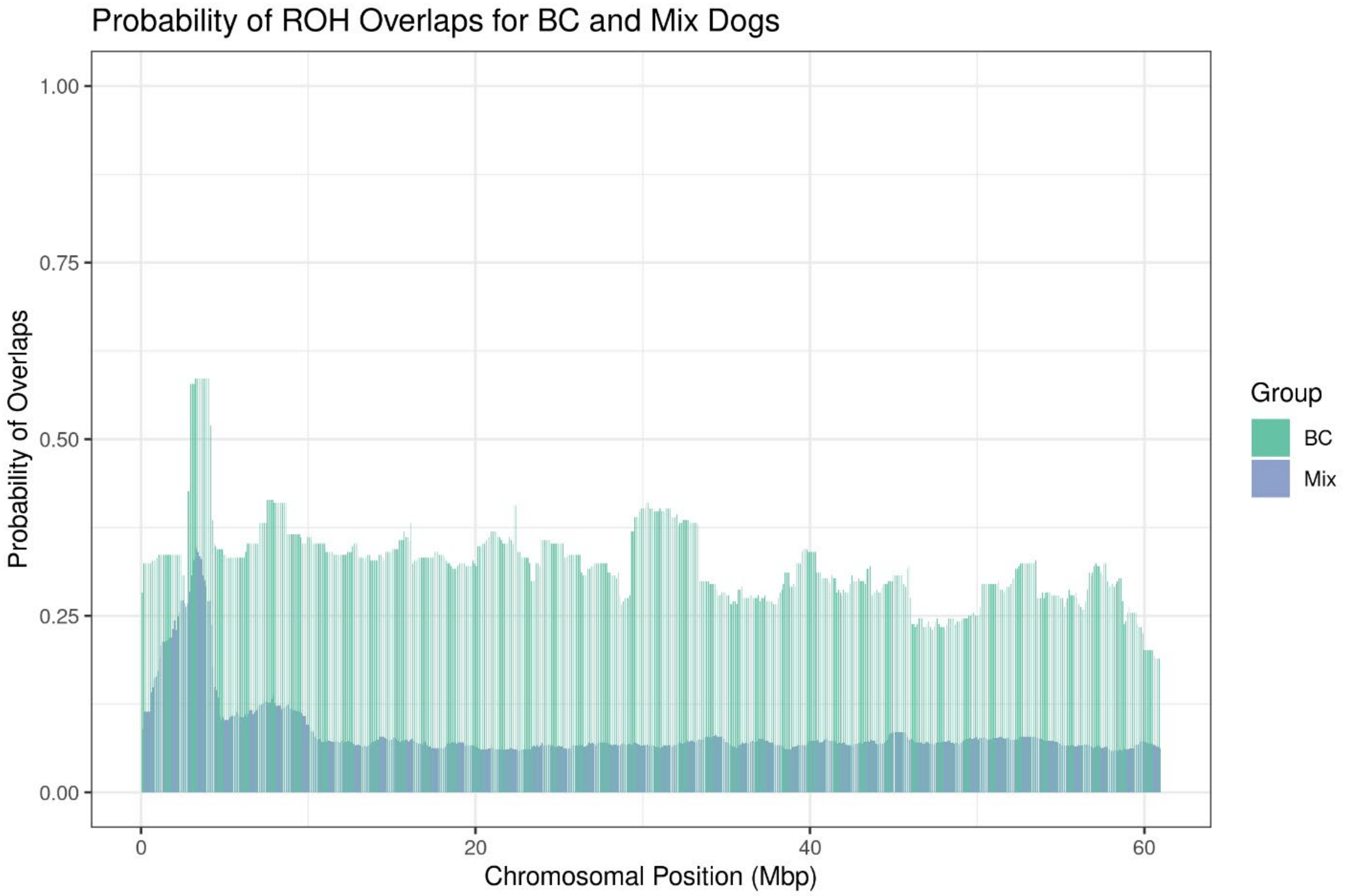

Probability of ROH Overlaps for BC and Mix Dogs
Probability of Overlaps
1.00
0.75
0.50
0.25
0.00
0
20
40
60
Chromosomal Position (Mbp)
Group
BC
Mix

CFA15

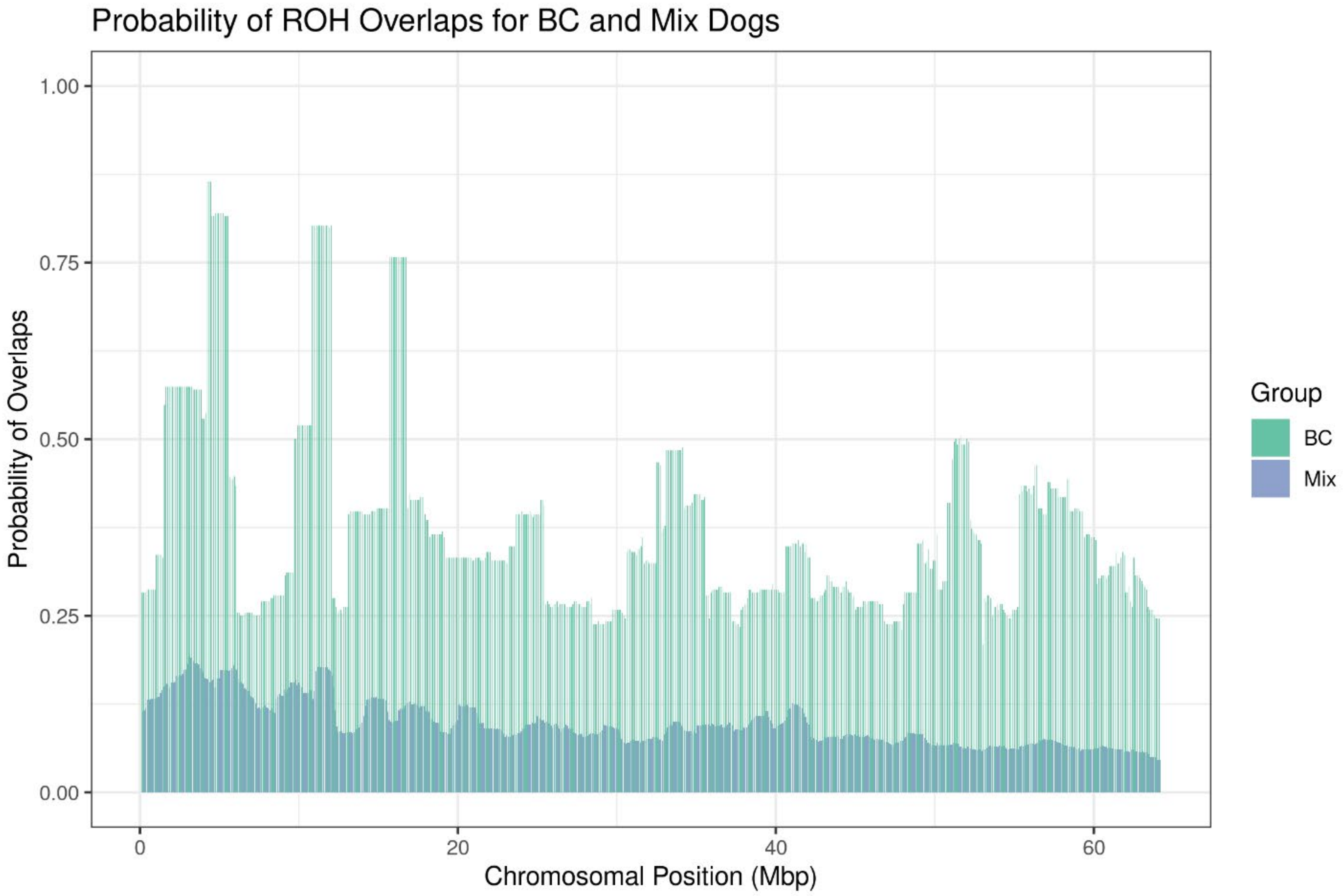

Probability of ROH Overlaps for BC and Mix Dogs
Probability of Overlaps
1.00
0.75
0.50
0.25
0.00
0
20
40
60
Chromosomal Position (Mbp)
Group
BC
Mix

CFA16

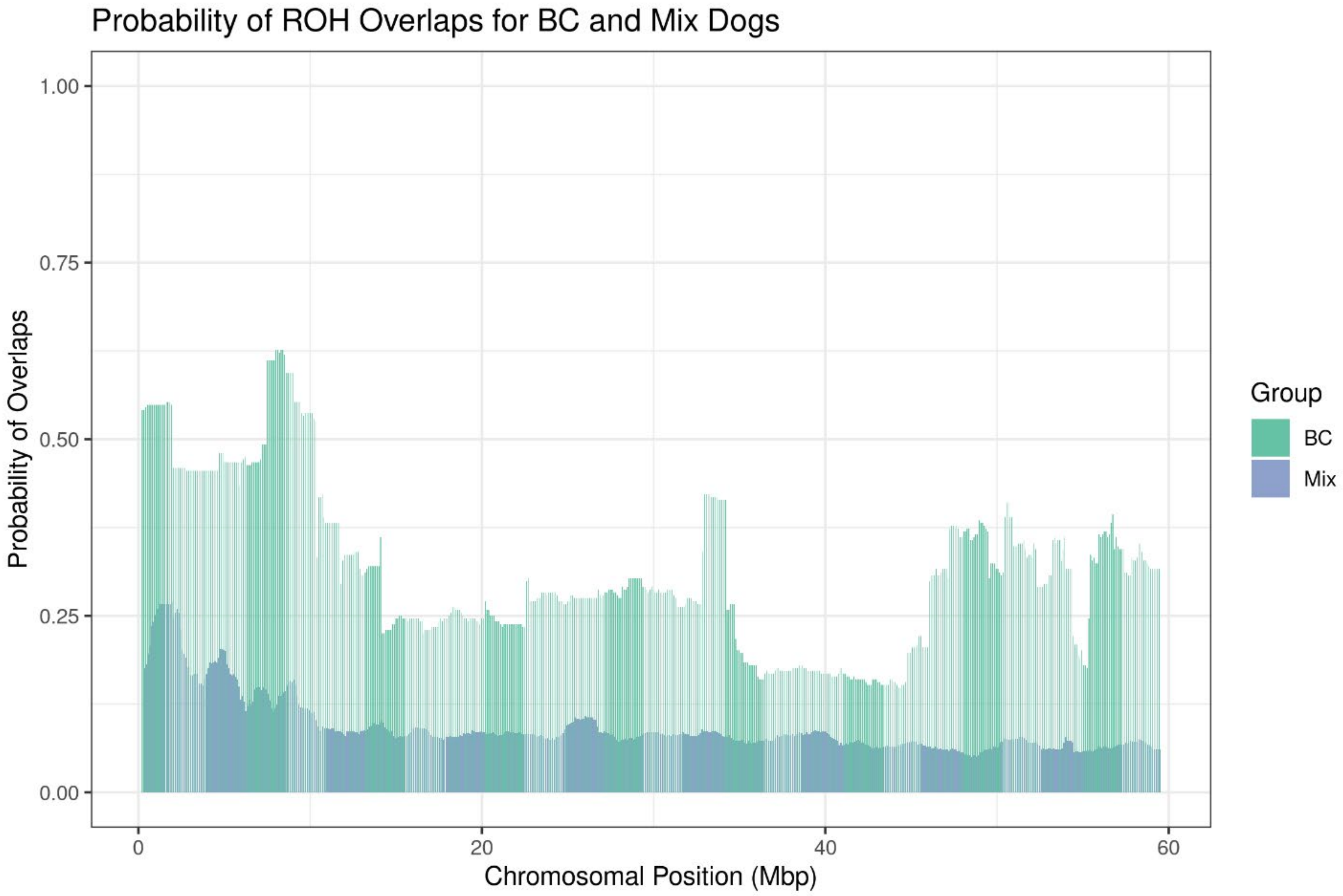
Probability of ROH Overlaps for BC and Mix Dogs
Probability of Overlaps
1.00
0.75
0.50
0.25
0.00
0
20
40
60
Chromosomal Position (Mbp)
Group
BC
Mix

CFA17

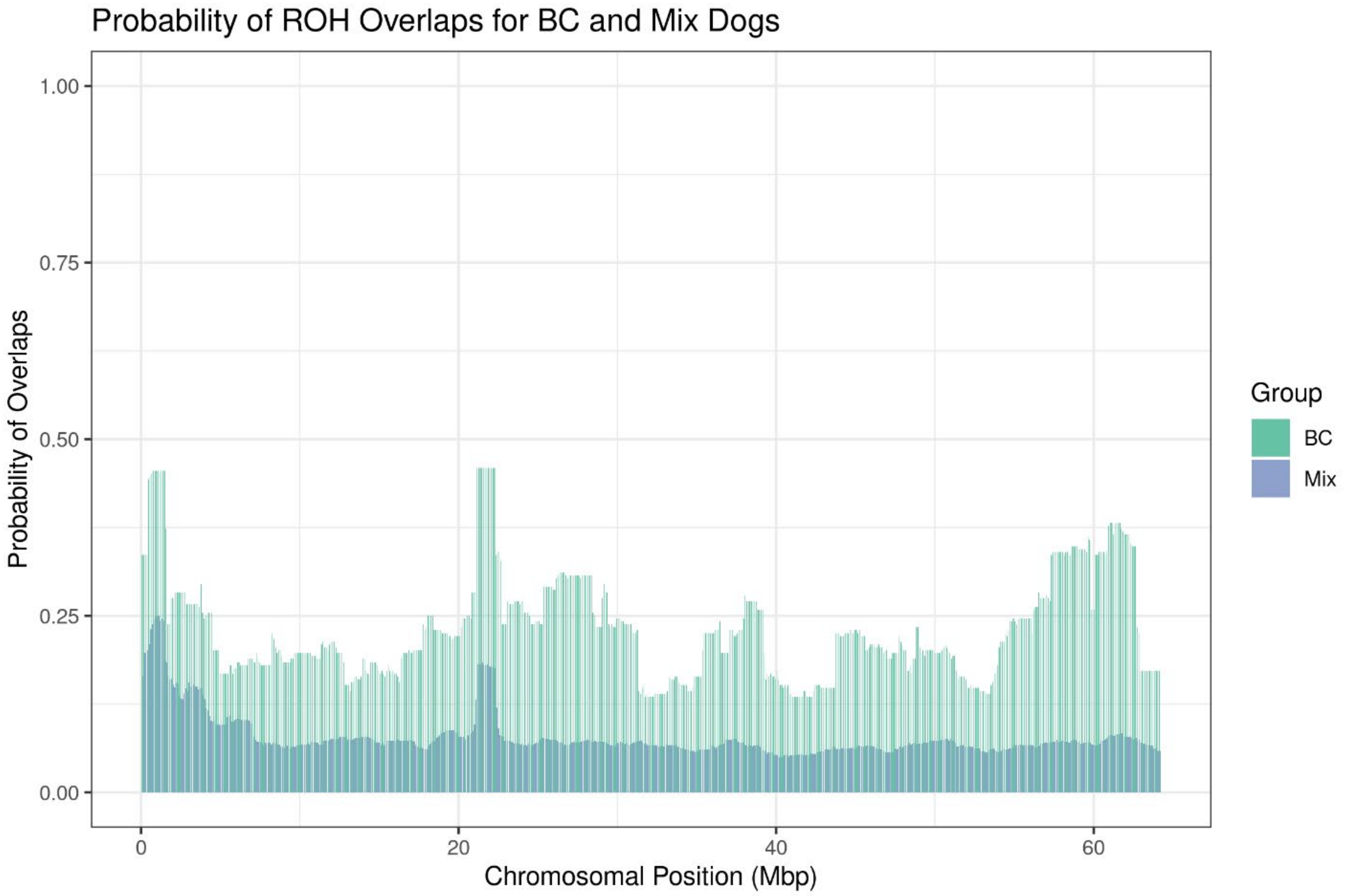

Probability of ROH Overlaps for BC and Mix Dogs
Probability of Overlaps
1.00
0.75
0.50
0.25
0.00
0
20
40
60
Chromosomal Position (Mbp)
Group
BC
Mix

CFA18

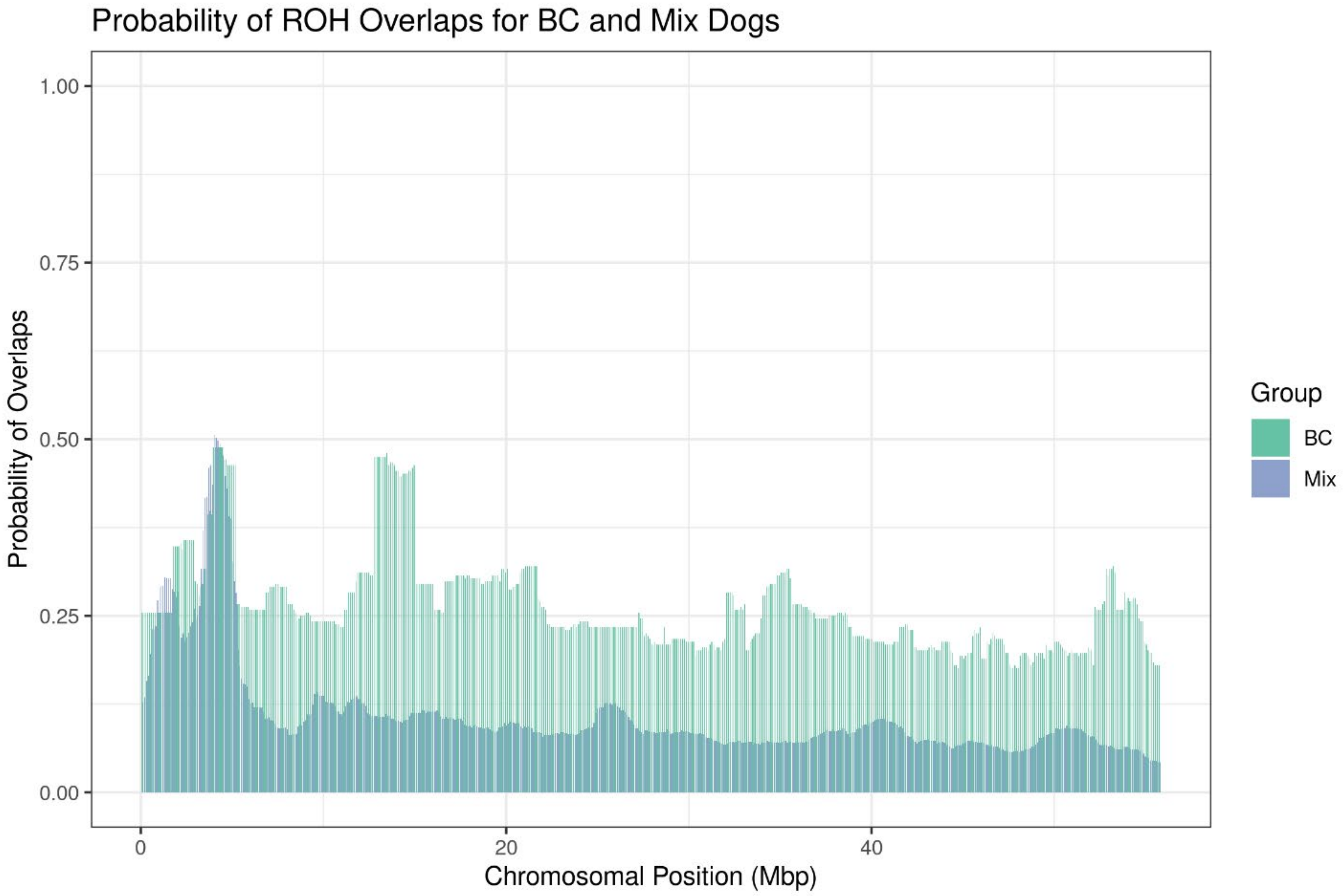
Probability of ROH Overlaps for BC and Mix Dogs
1.00
0.75
0.50
0.25
0.00
Probability of Overlaps
0
20
40
Chromosomal Position (Mbp)
Group
BC
Mix

CFA19

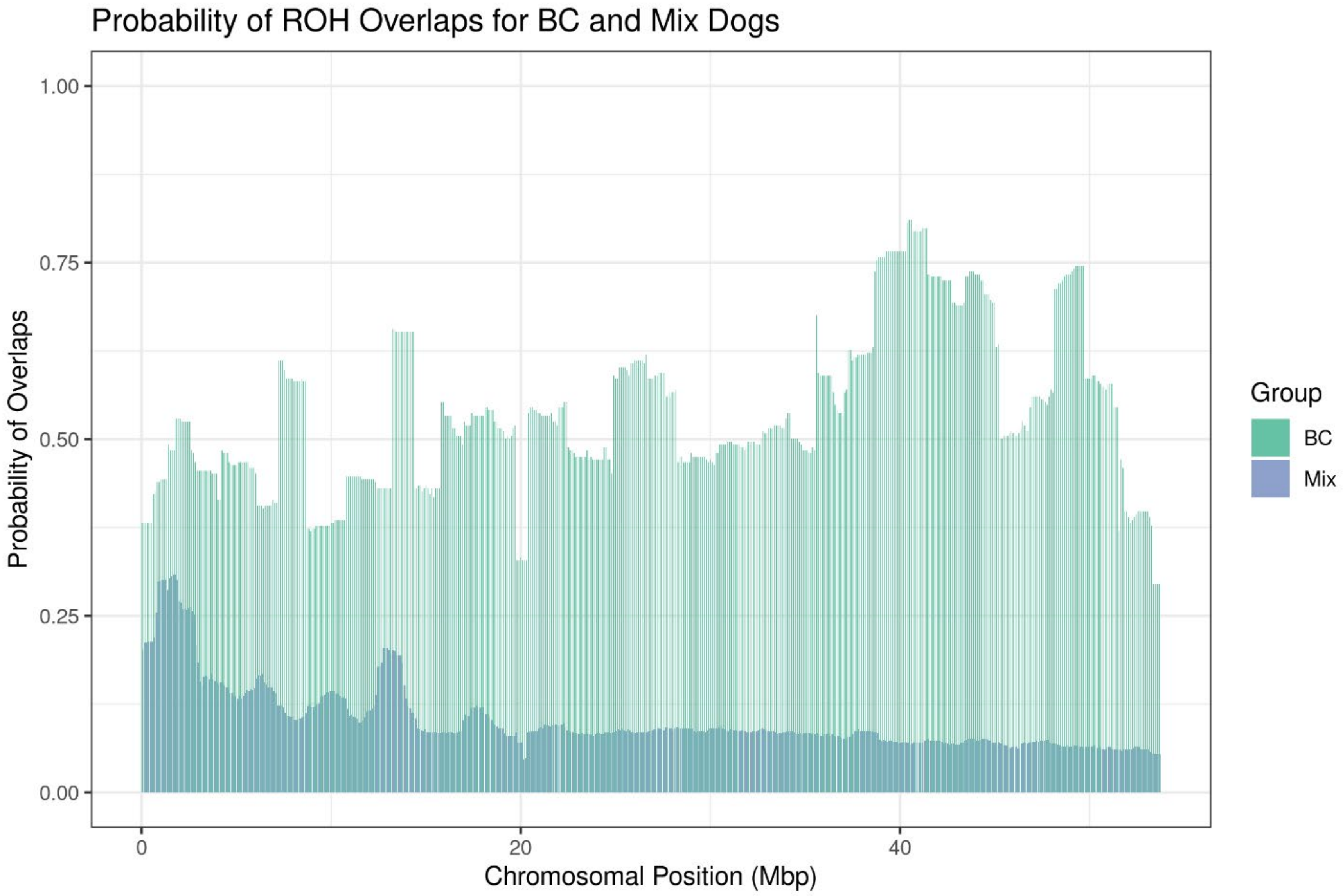

Probability of ROH Overlaps for BC and Mix Dogs
1.00
0.75
0.50
0.25
0.00
Probability of Overlaps
0
20
40
Chromosomal Position (Mbp)
Group
BC
Mix

CFA20

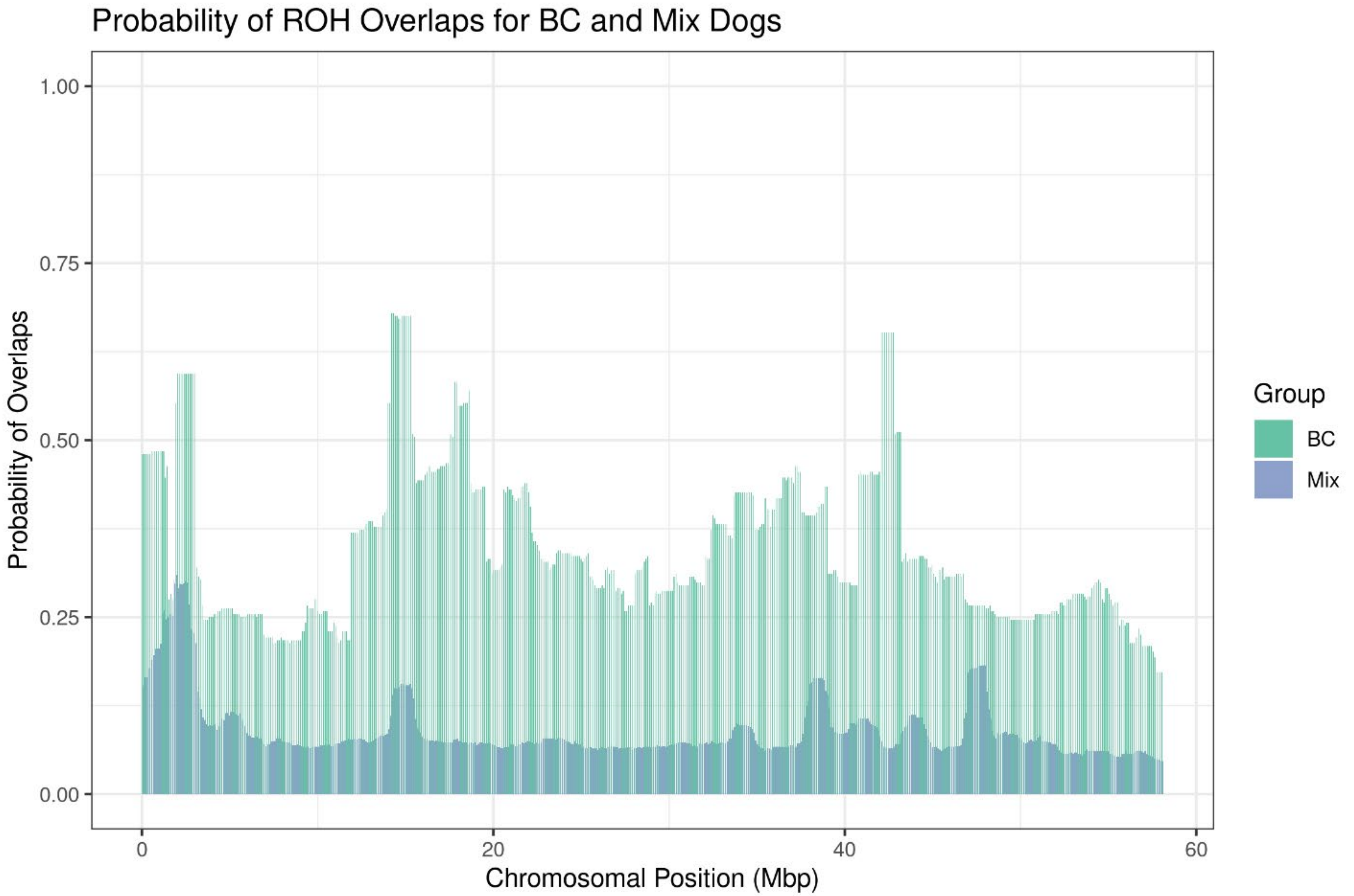

Probability of ROH Overlaps for BC and Mix Dogs
Probability of Overlaps
1.00
0.75
0.50
0.25
0.00
0
20
40
60
Chromosomal Position (Mbp)
Group
BC
Mix

CFA21

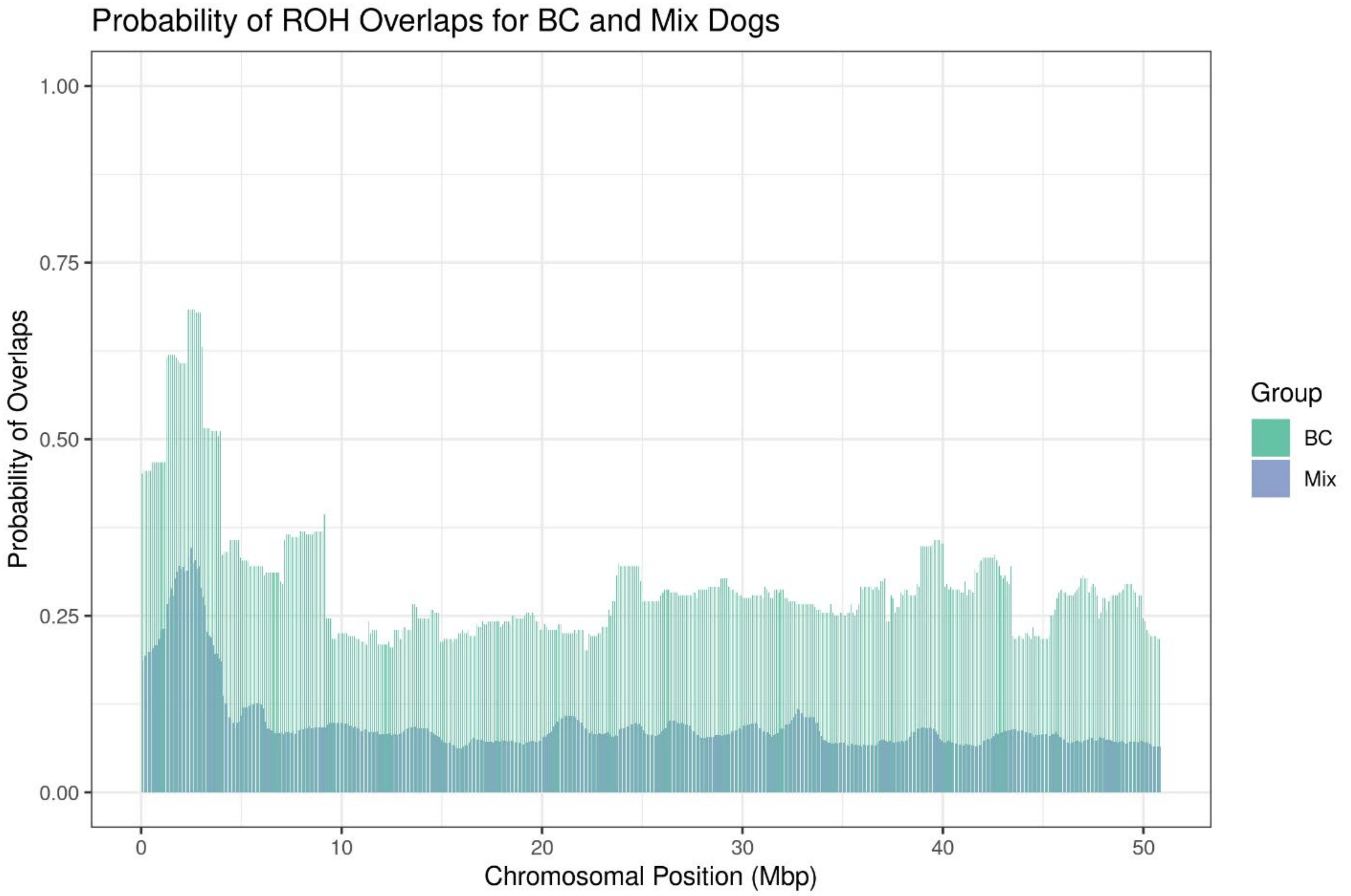

Probability of ROH Overlaps for BC and Mix Dogs
Probability of Overlaps
1.00
0.75
0.50
0.25
0.00
0
10
20
30
40
50
Chromosomal Position (Mbp)
Group
BC
Mix

CFA22

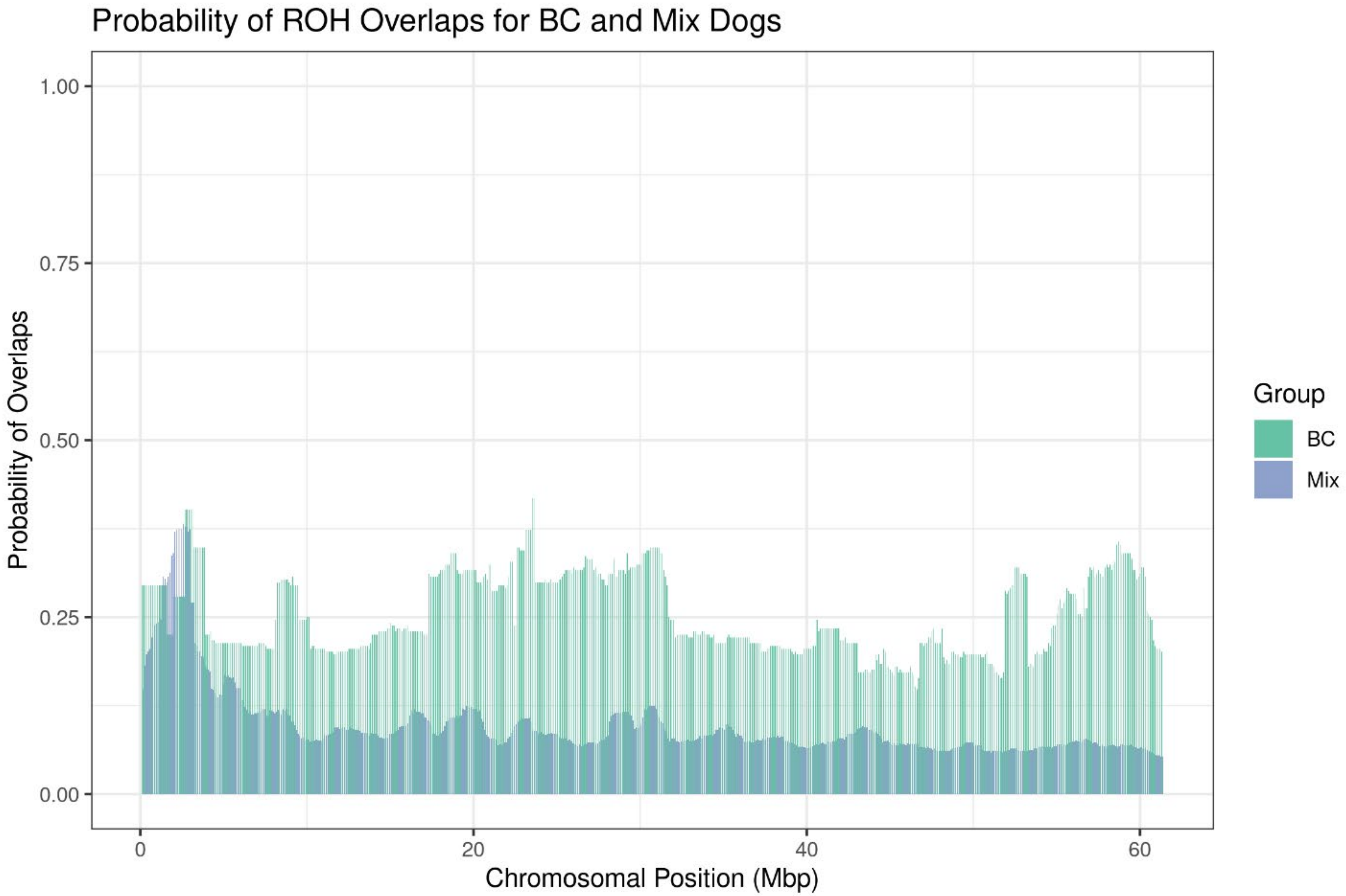

Probability of ROH Overlaps for BC and Mix Dogs
1.00
0.75
0.50
0.25
0.00
Probability of Overlaps
0
20
40
60
Chromosomal Position (Mbp)
Group
BC
Mix

CFA23

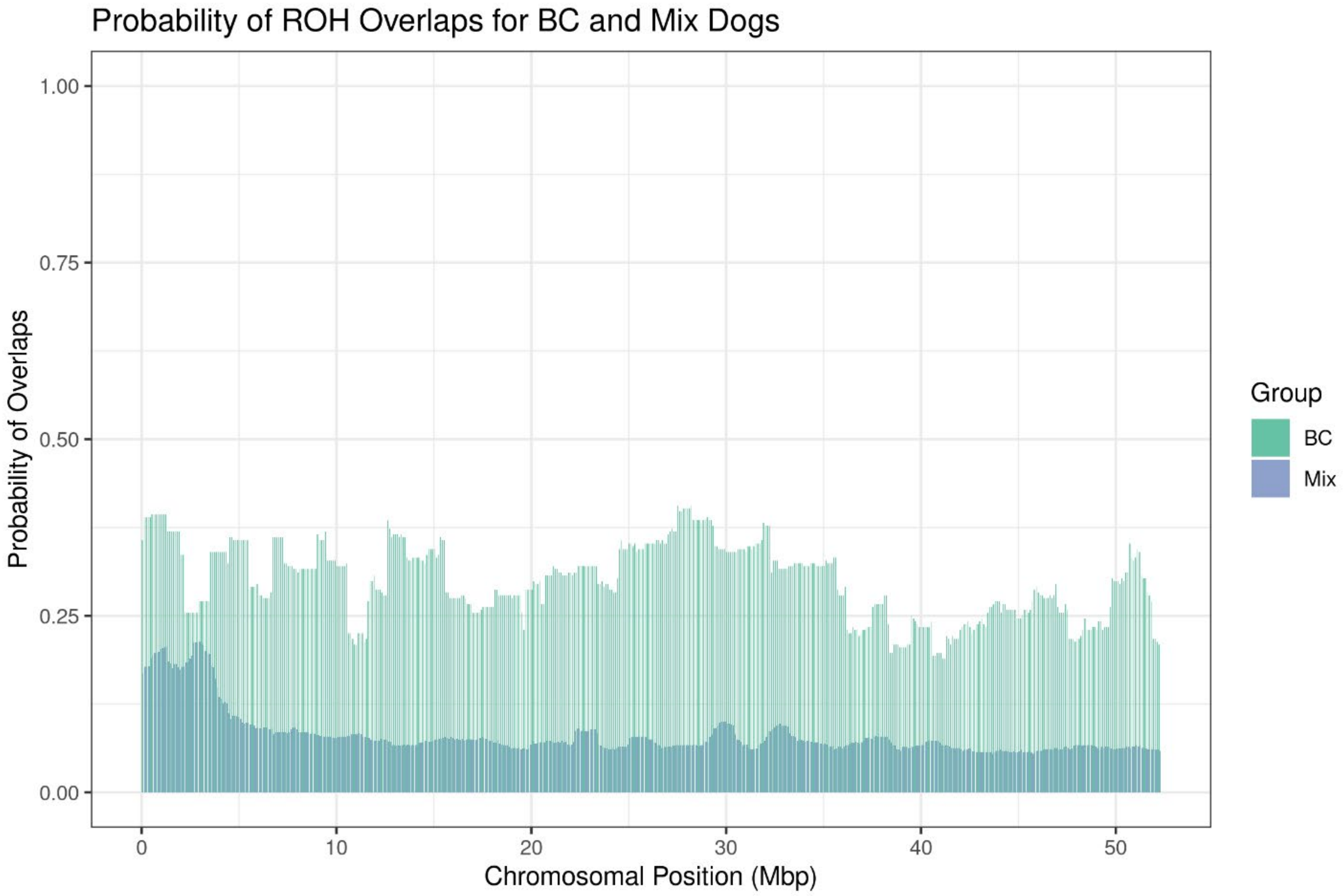
Probability of ROH Overlaps for BC and Mix Dogs
Probability of Overlaps
1.00
0.75
0.50
0.25
0.00
0
10
20
30
40
50
Chromosomal Position (Mbp)
Group
BC
Mix

CFA24

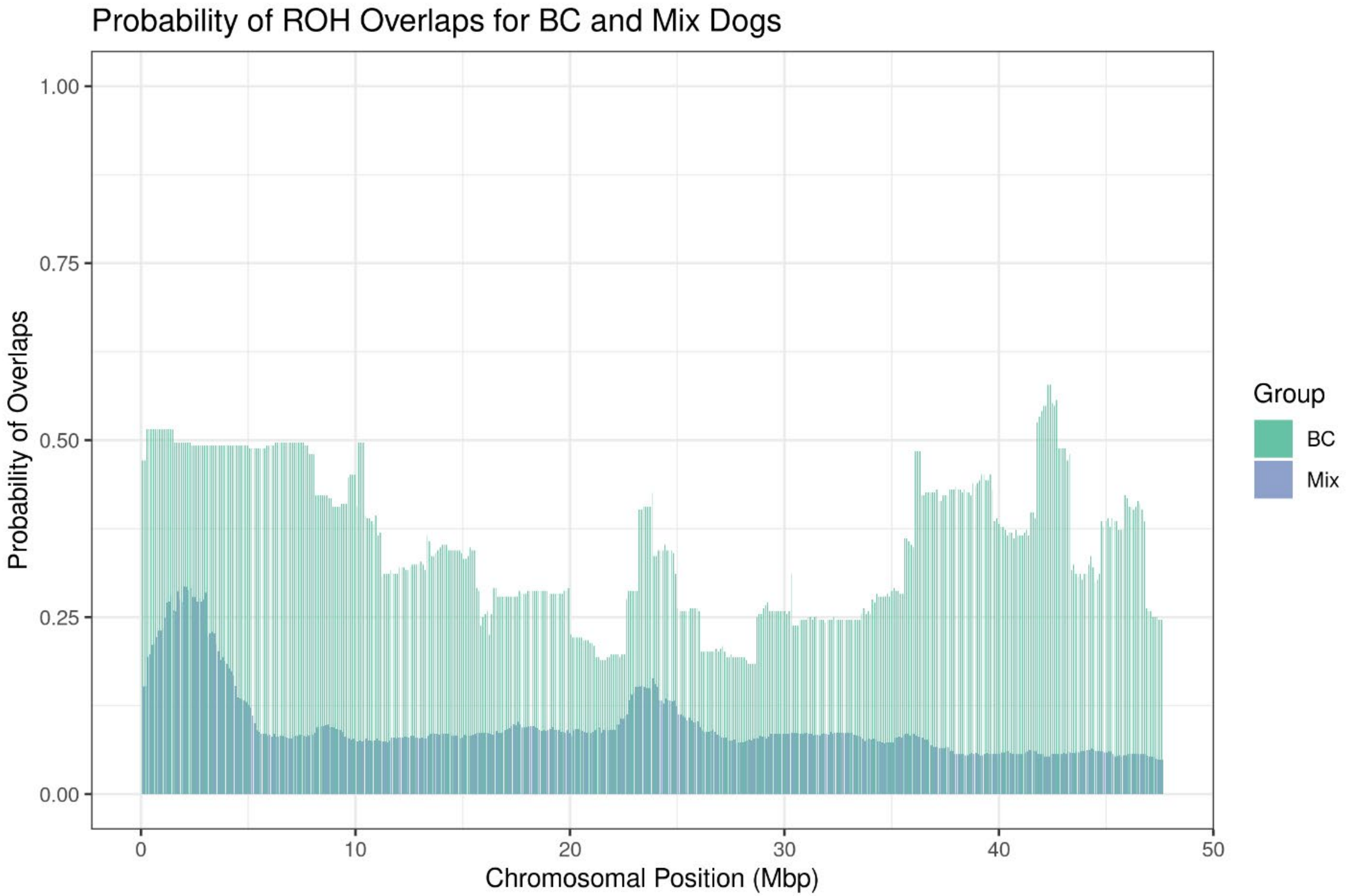
Probability of ROH Overlaps for BC and Mix Dogs
Probability of Overlaps
1.00
0.75
0.50
0.25
0.00
0
10
20
30
40
50
Chromosomal Position (Mbp)
Group
BC
Mix

CFA25

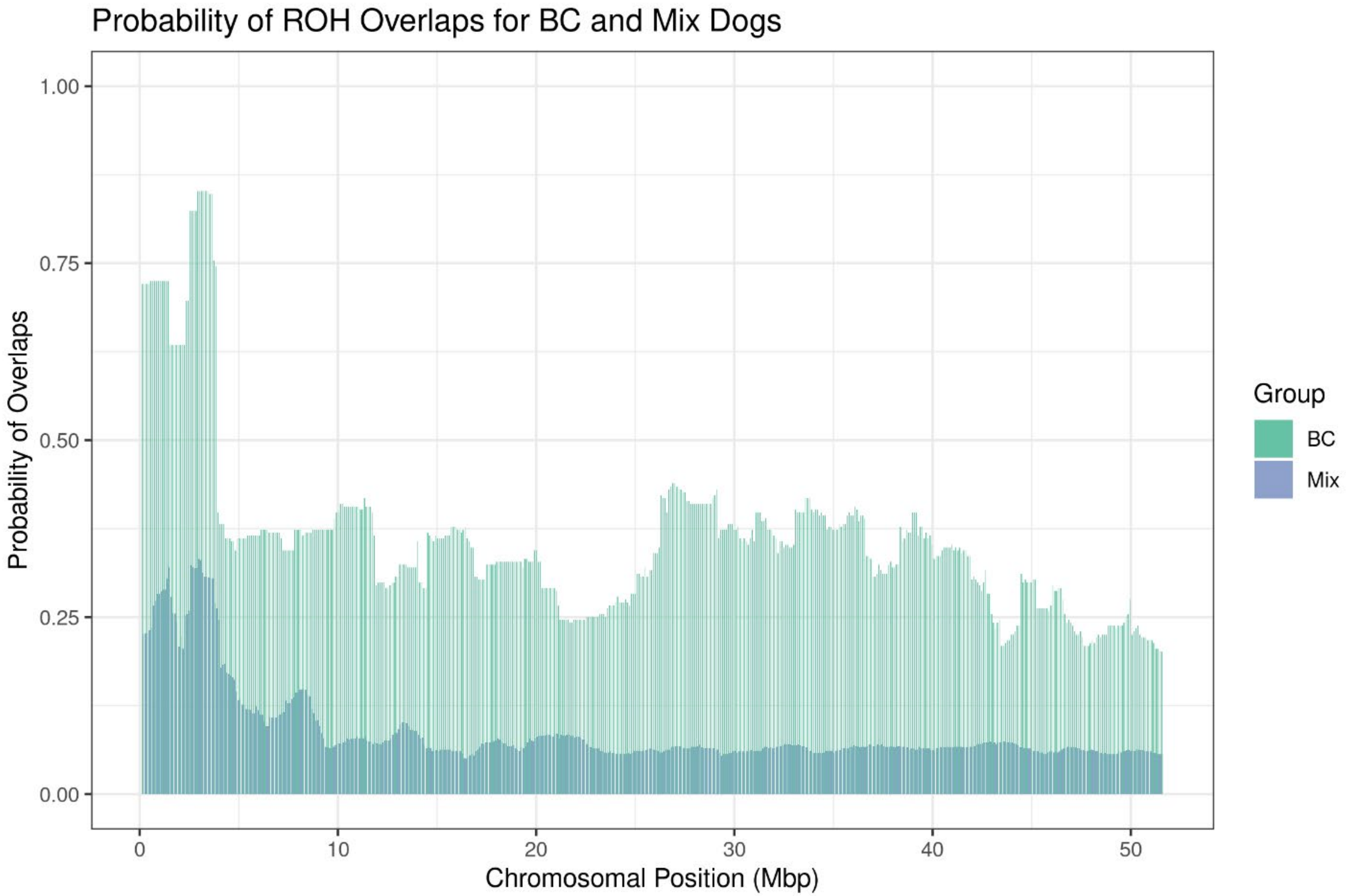

Probability of ROH Overlaps for BC and Mix Dogs
Probability of Overlaps
1.00
0.75
0.50
0.25
0.00
0
10
20
30
40
50
Chromosomal Position (Mbp)
Group
BC
Mix

CFA26

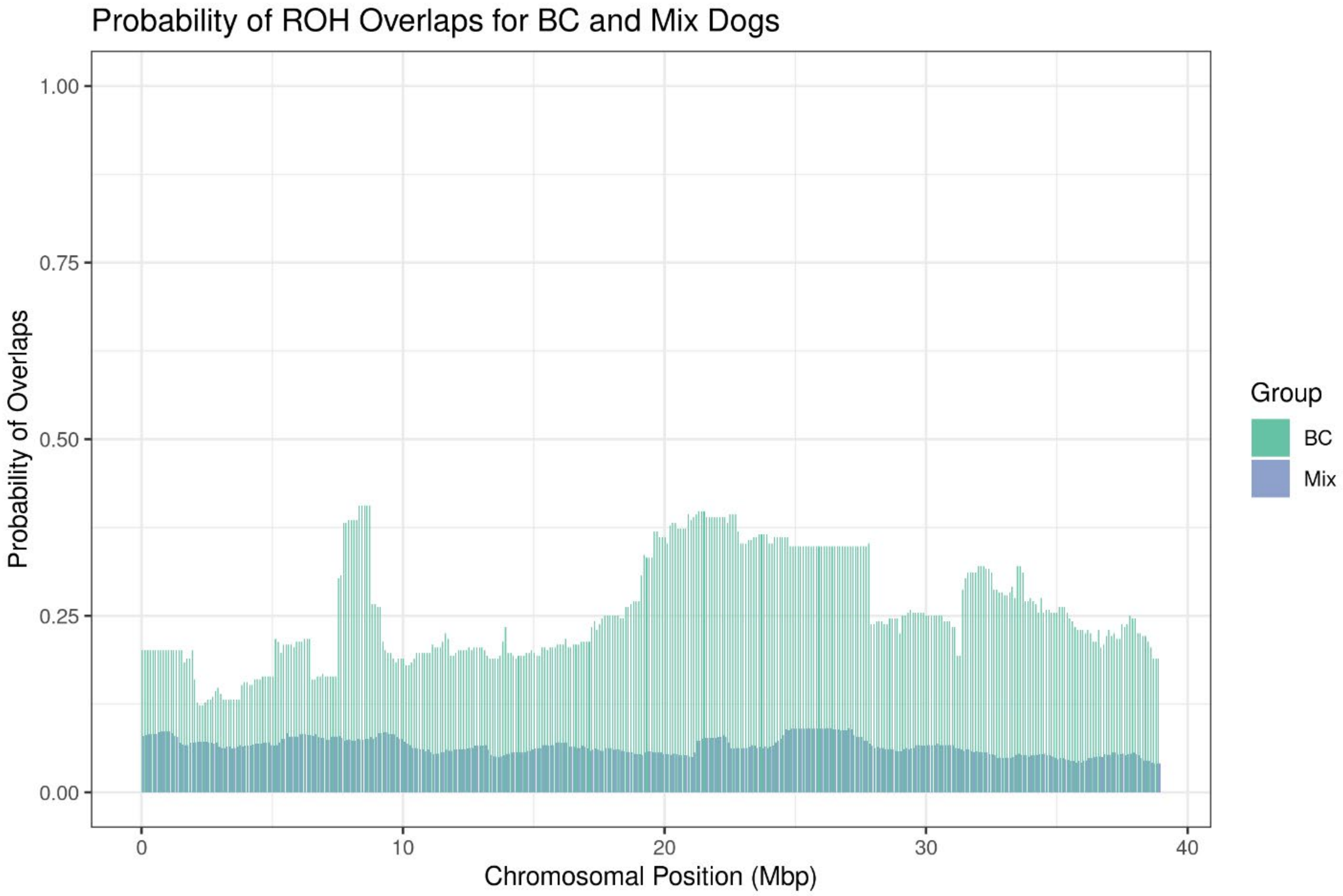

Probability of ROH Overlaps for BC and Mix Dogs
1.00
0.75
0.50
0.25
0.00
Probability of Overlaps
0
10
20
30
40
Chromosomal Position (Mbp)
Group
BC
Mix

CFA27

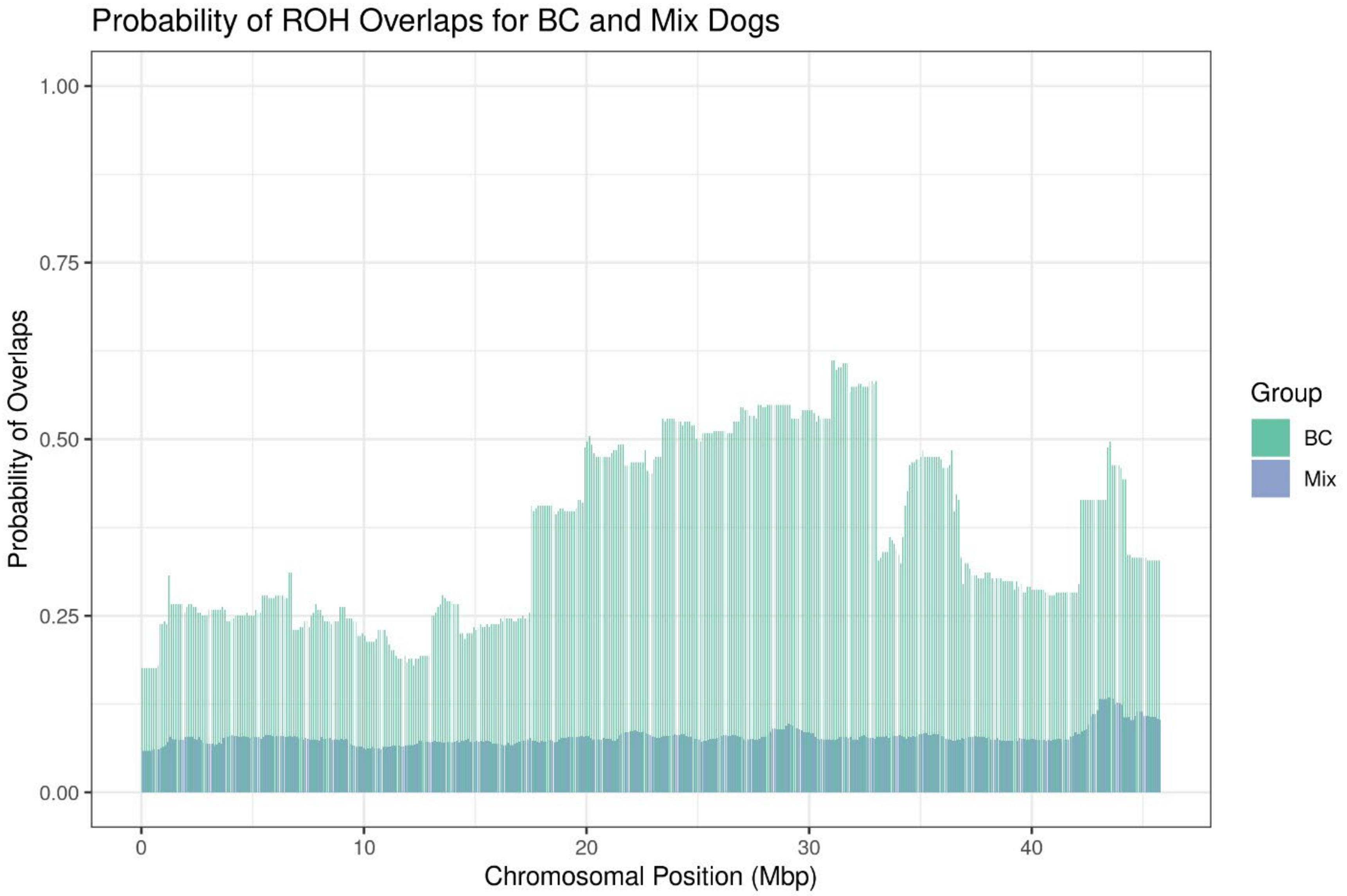

Probability of ROH Overlaps for BC and Mix Dogs
Probability of Overlaps
1.00
0.75
0.50
0.25
0.00
0
10
20
30
40
Chromosomal Position (Mbp)
Group
BC
Mix

CFA28

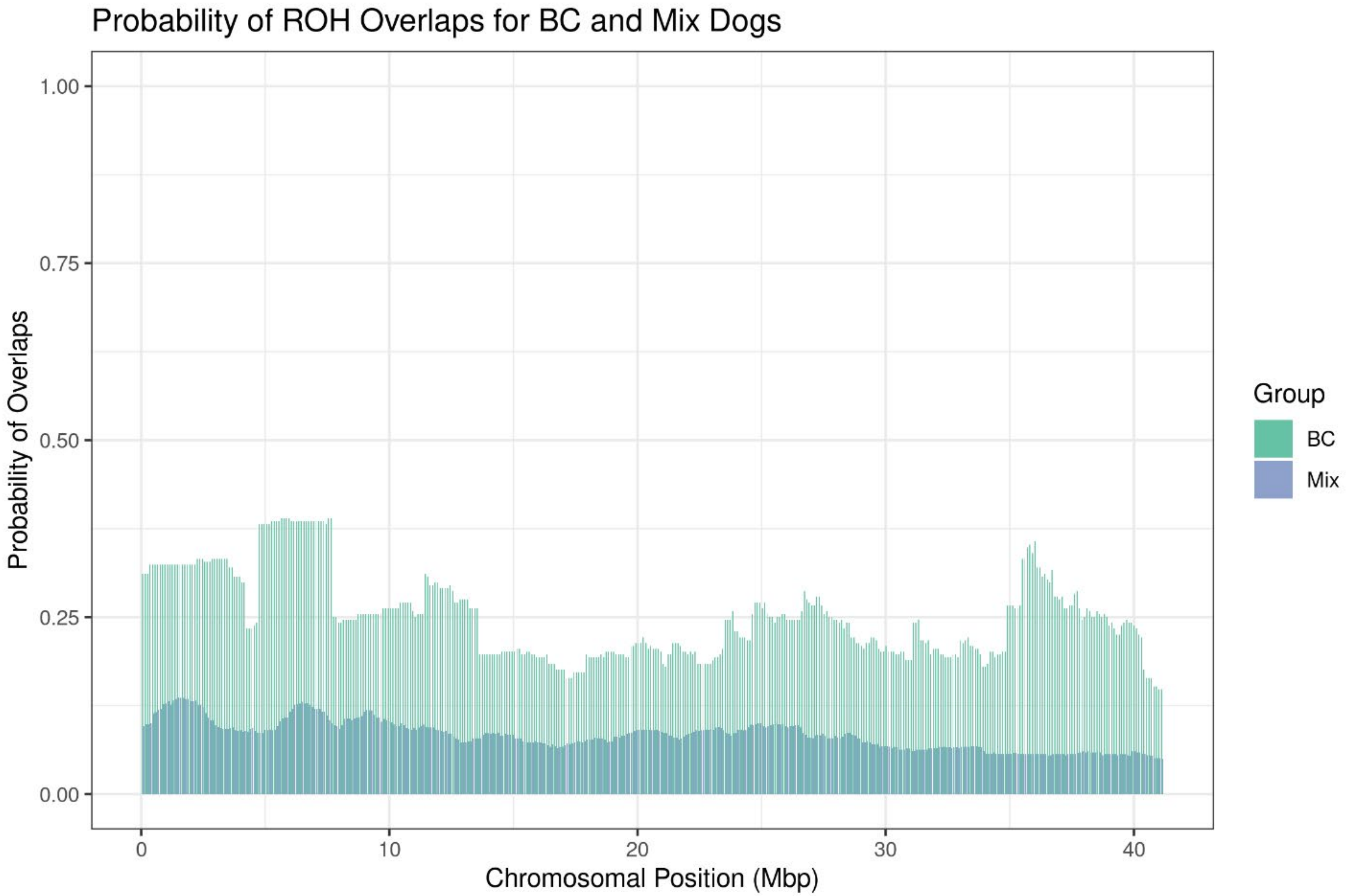

Probability of ROH Overlaps for BC and Mix Dogs
1.00
0.75
0.50
0.25
0.00
Probability of Overlaps
0
10
20
30
40
Chromosomal Position (Mbp)
Group
BC
Mix

CFA29

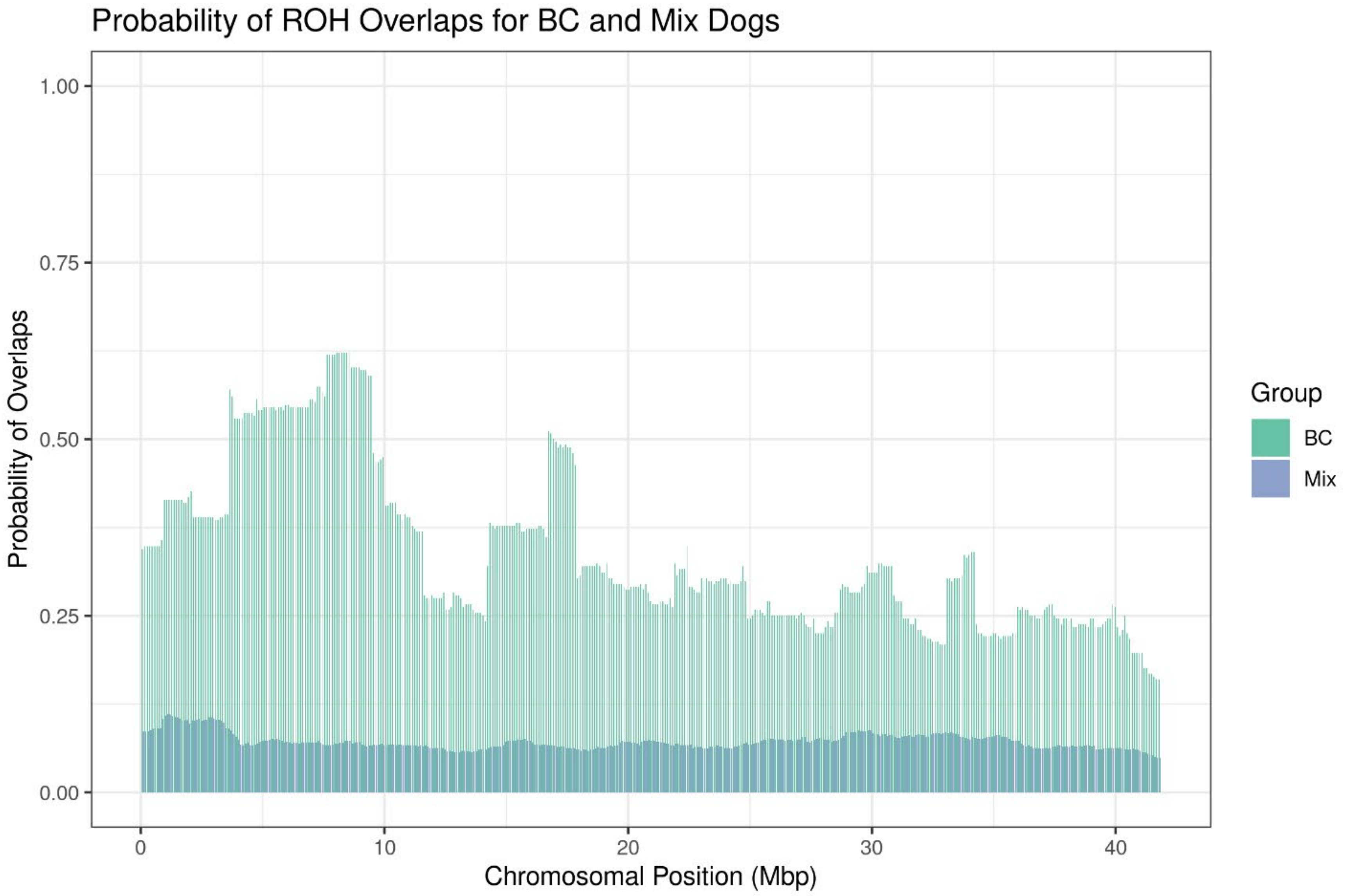

Probability of ROH Overlaps for BC and Mix Dogs
Probability of Overlaps
1.00
0.75
0.50
0.25
0.00
0
10
20
30
40
Chromosomal Position (Mbp)
Group
BC
Mix

CFA30

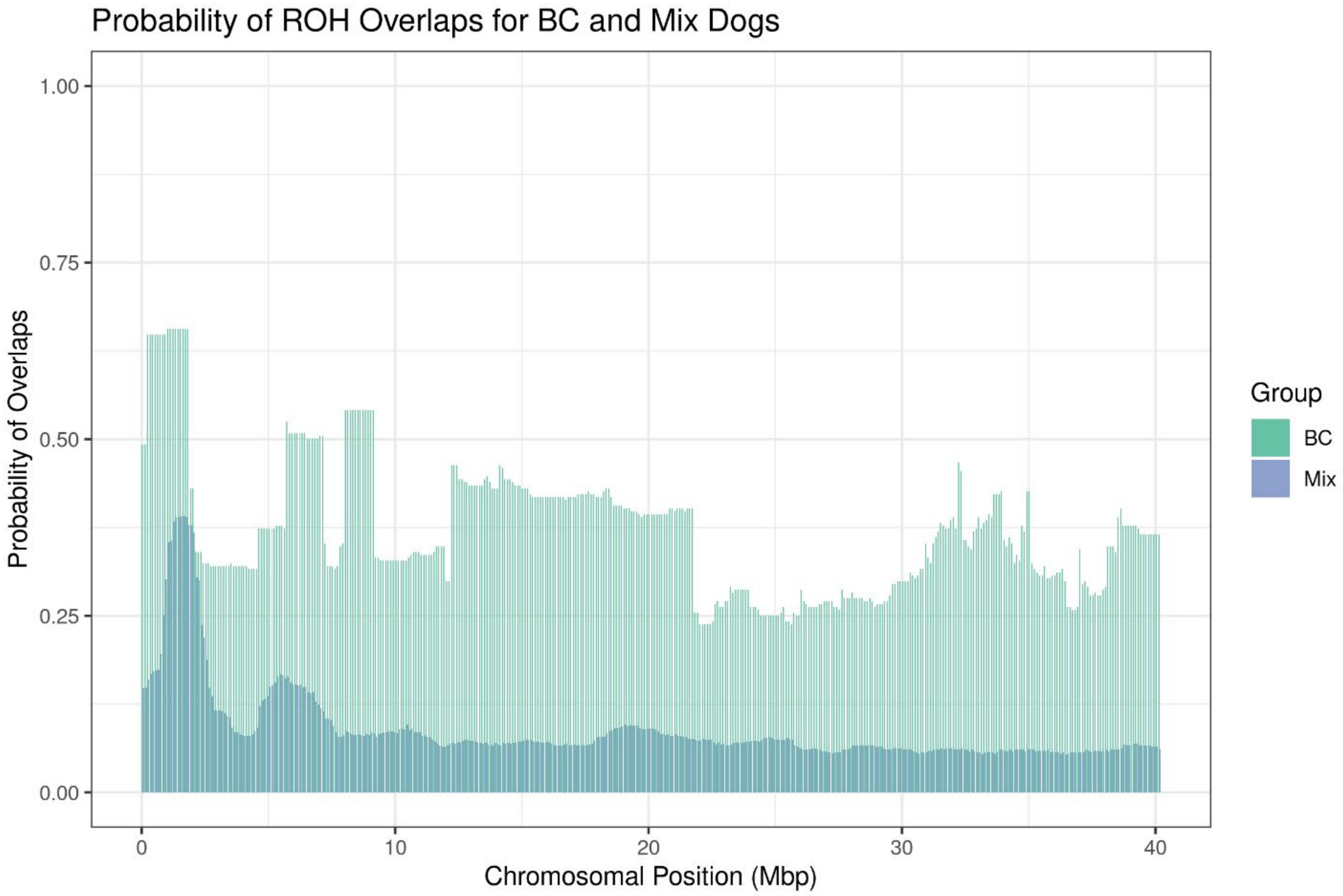

Probability of ROH Overlaps for BC and Mix Dogs
1.00
0.75
0.50
0.25
0.00
Probability of Overlaps
0
10
20
30
40
Chromosomal Position (Mbp)
Group
BC
Mix

CFA31

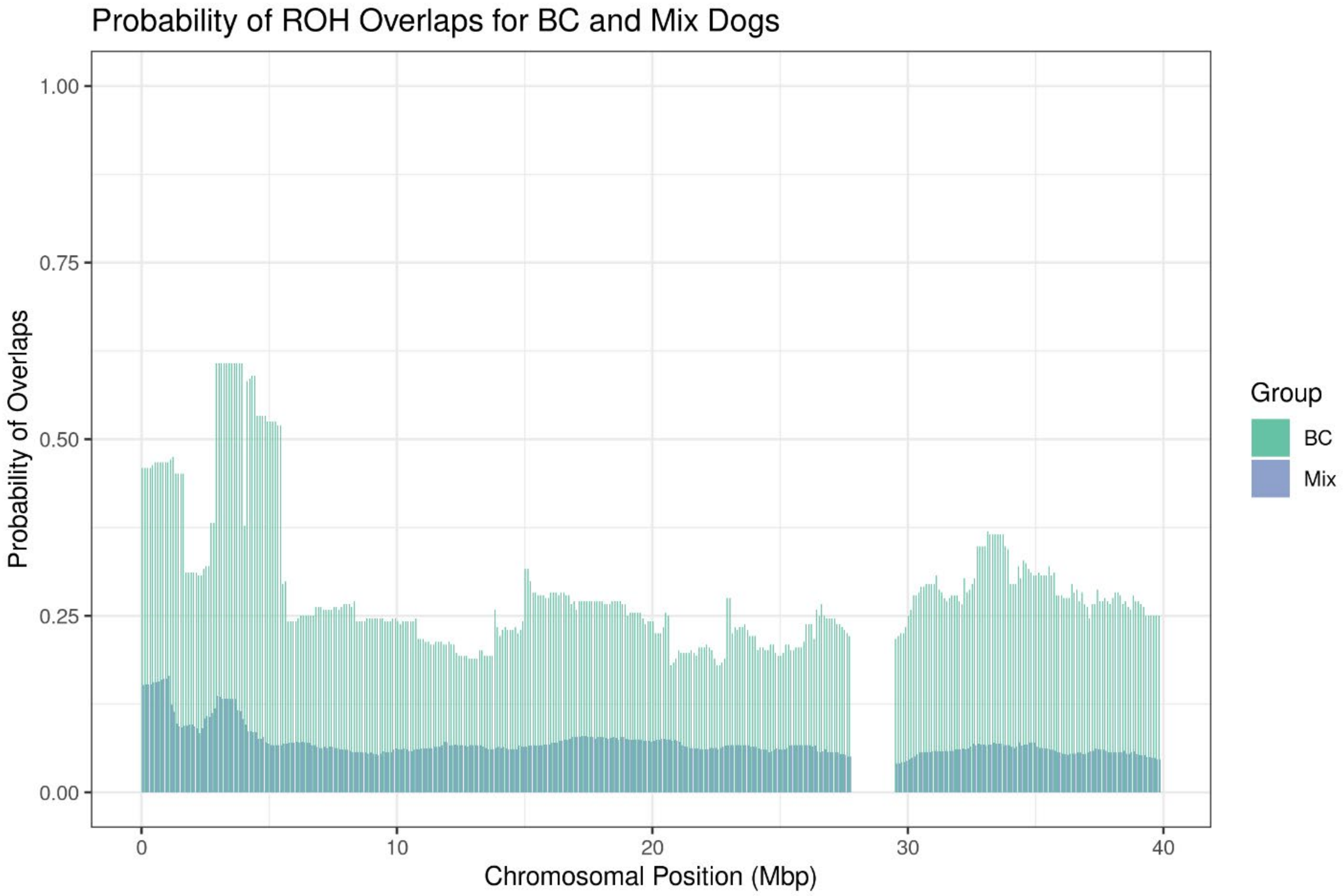

Probability of ROH Overlaps for BC and Mix Dogs
Probability of Overlaps
1.00
0.75
0.50
0.25
0.00
0
10
20
30
40
Chromosomal Position (Mbp)
Group
BC
Mix

CFA32

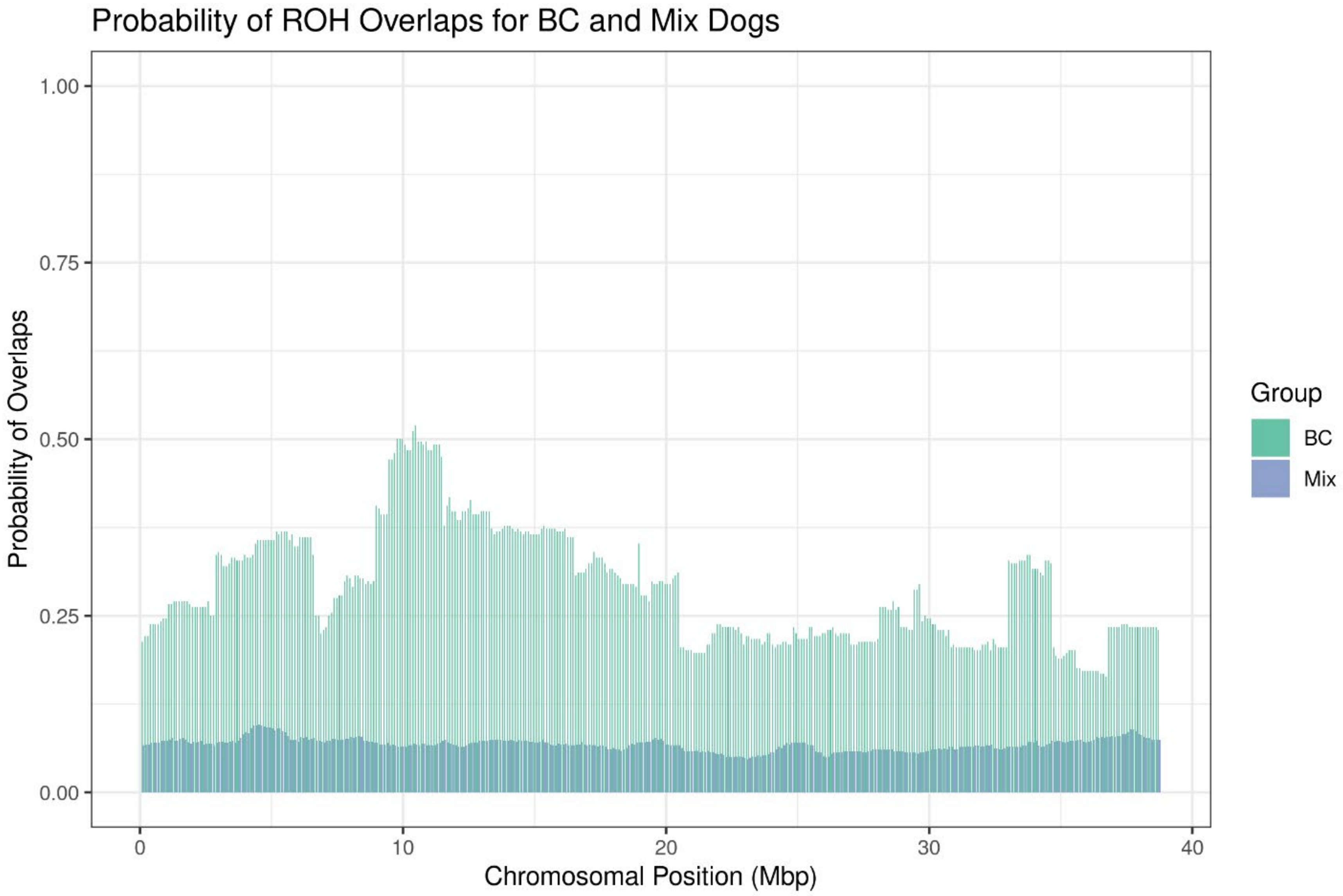

Probability of ROH Overlaps for BC and Mix Dogs
Probability of Overlaps
1.00
0.75
0.50
0.25
0.00
0
10
20
30
40
Chromosomal Position (Mbp)
Group
BC
Mix

CFA33

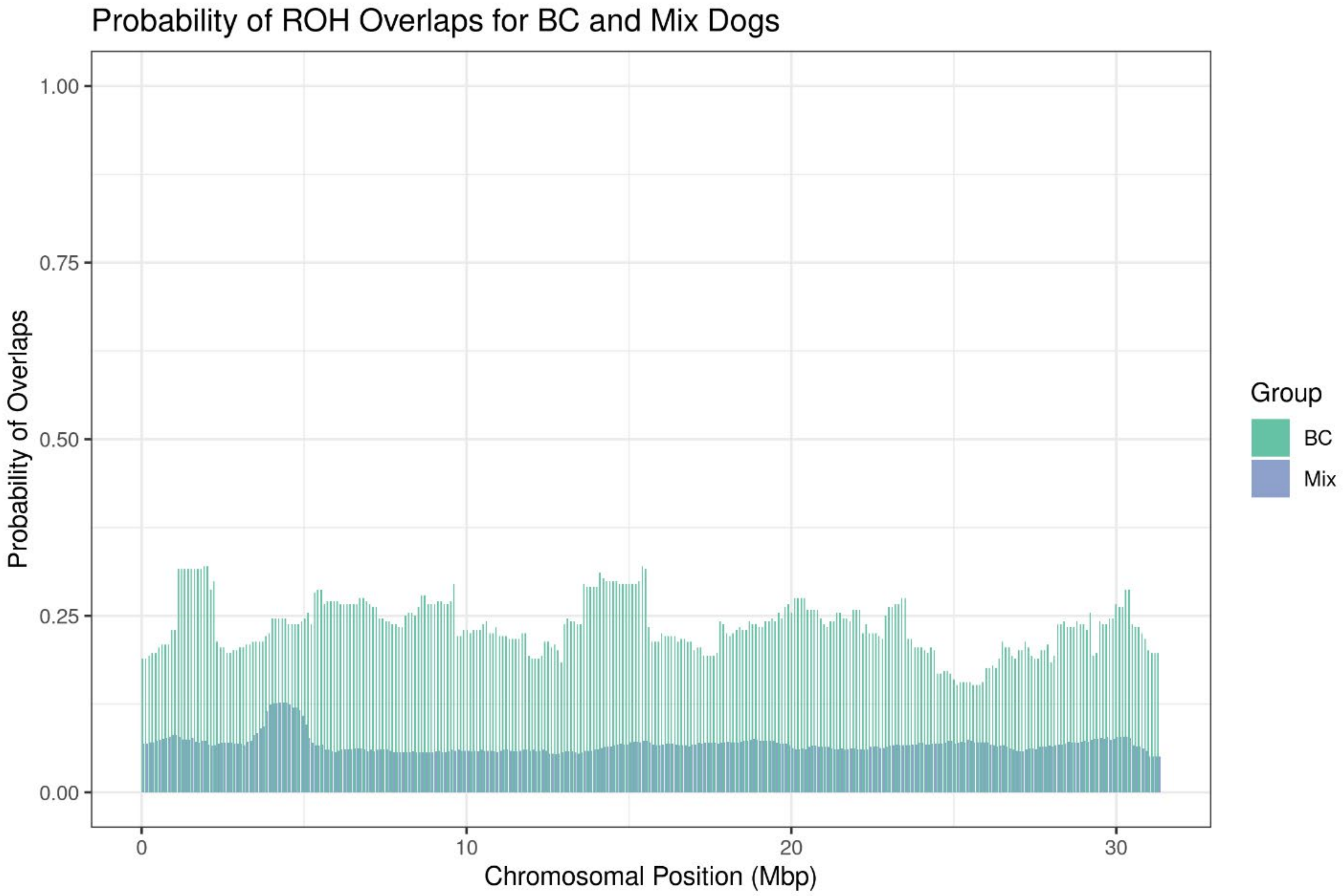

Probability of ROH Overlaps for BC and Mix Dogs
1.00
0.75
0.50
0.25
0.00
Probability of Overlaps
0
10
20
30
Chromosomal Position (Mbp)
Group
BC
Mix

CFA34

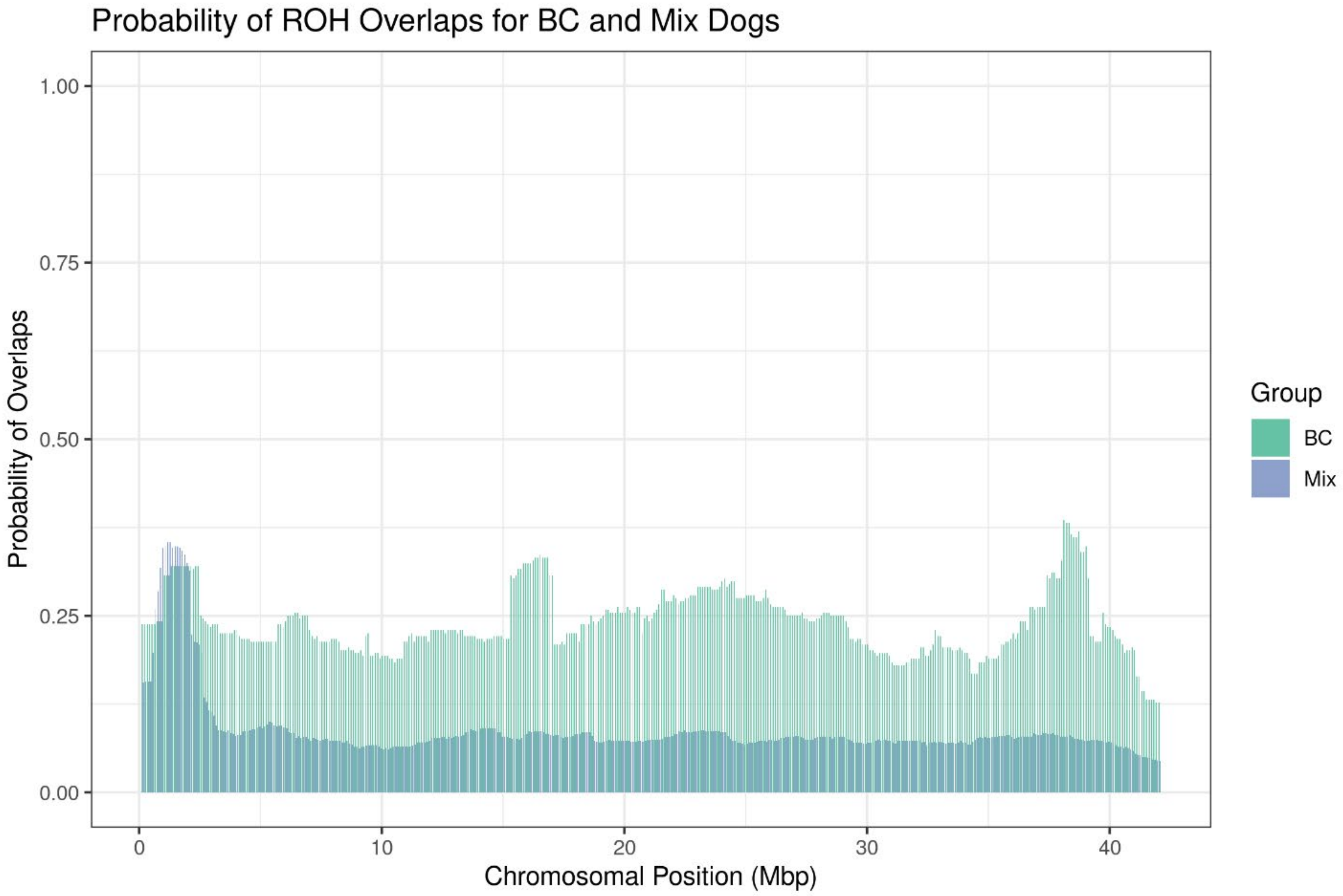

Probability of ROH Overlaps for BC and Mix Dogs
Probability of Overlaps
1.00
0.75
0.50
0.25
0.00
0
10
20
30
40
Chromosomal Position (Mbp)
Group
BC
Mix

CFA35

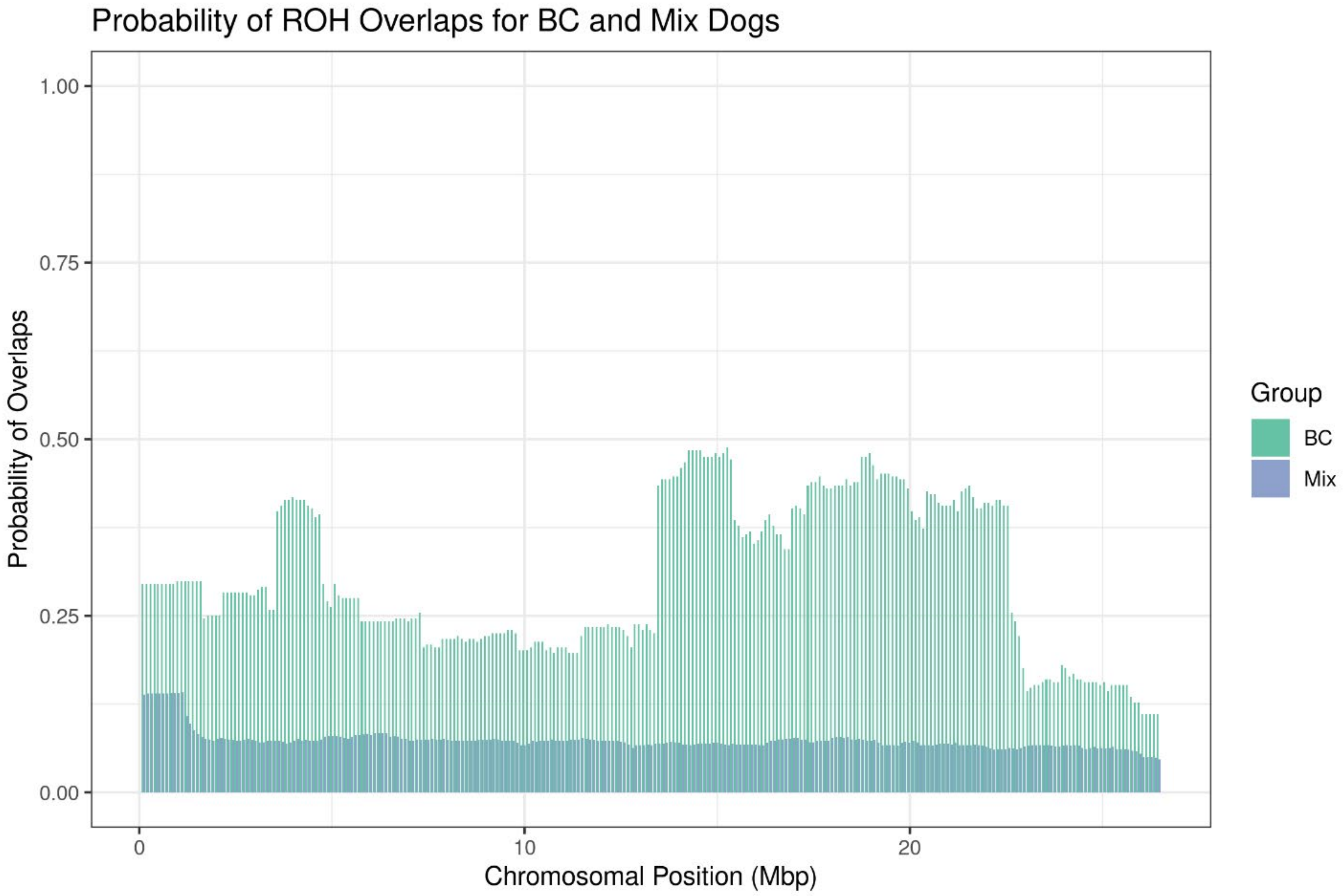
Probability of ROH Overlaps for BC and Mix Dogs
Probability of Overlaps
1.00
0.75
0.50
0.25
0.00
0
10
20
Chromosomal Position (Mbp)
Group
BC
Mix

CFA36

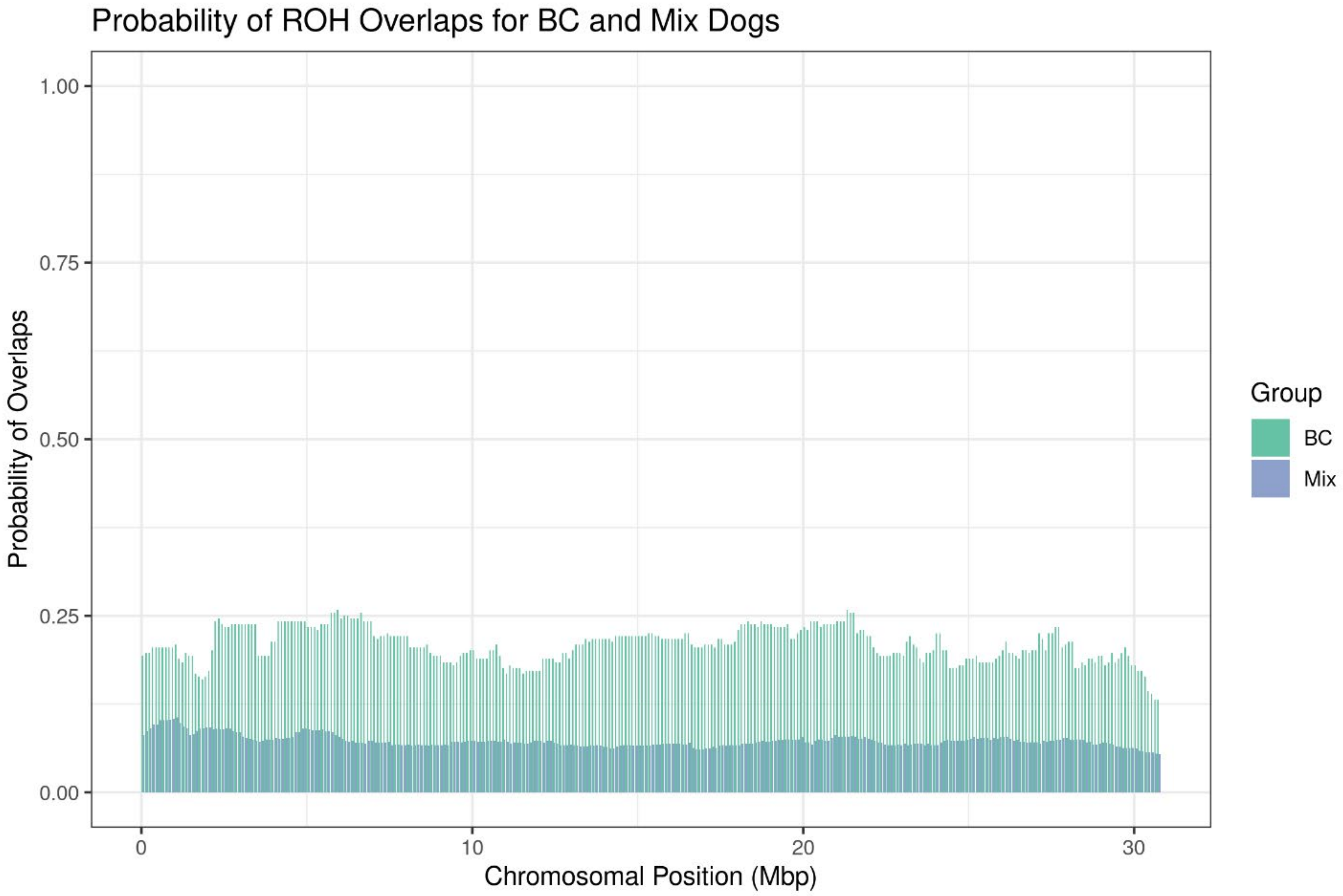

Probability of ROH Overlaps for BC and Mix Dogs
Probability of Overlaps
1.00
0.75
0.50
0.25
0.00
0
10
20
30
Chromosomal Position (Mbp)
Group
BC
Mix

CFA37

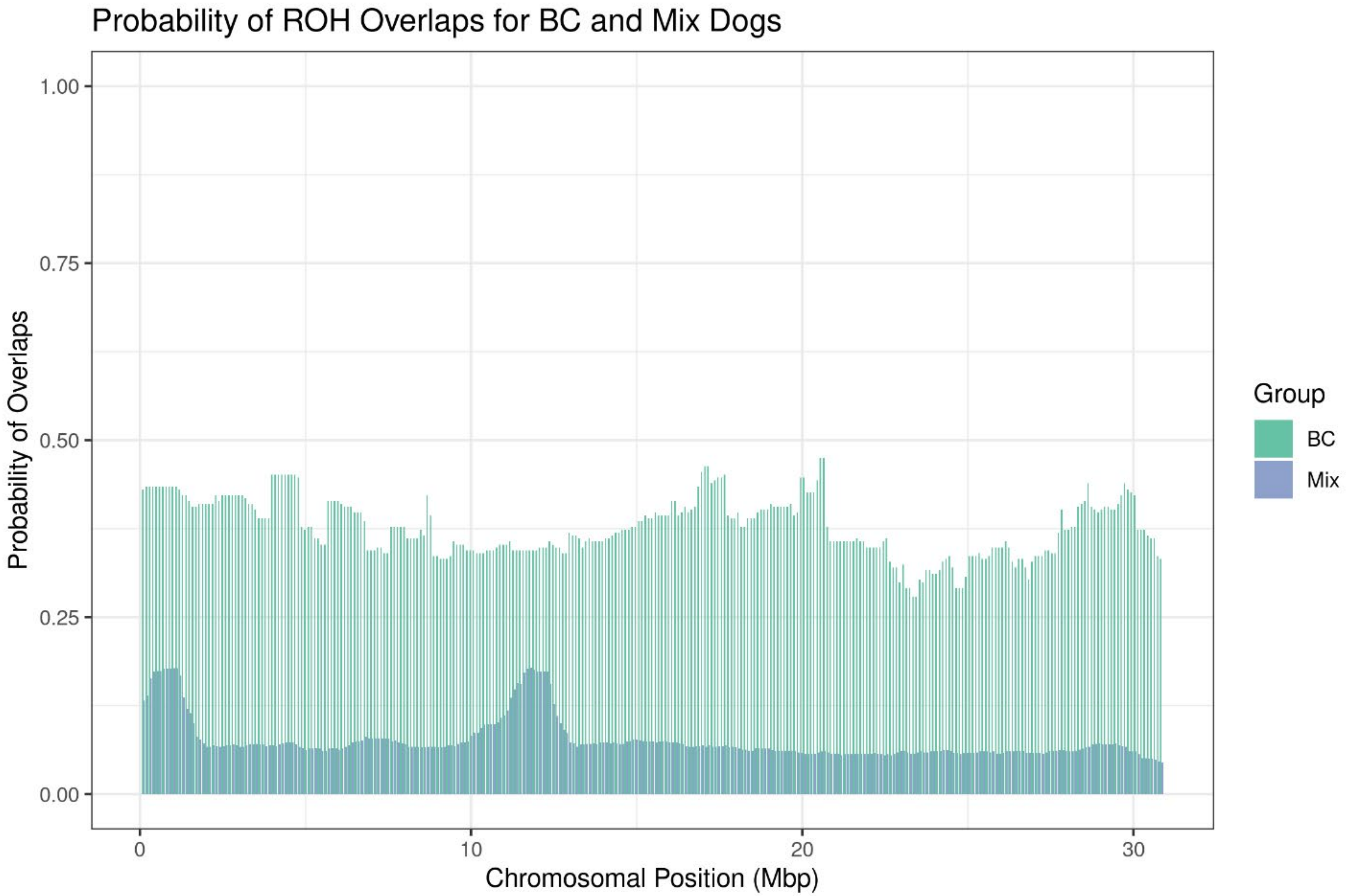
Probability of ROH Overlaps for BC and Mix Dogs
Probability of Overlaps
1.00
0.75
0.50
0.25
0.00
0
10
20
30
Chromosomal Position (Mbp)
Group
BC
Mix

CFA38

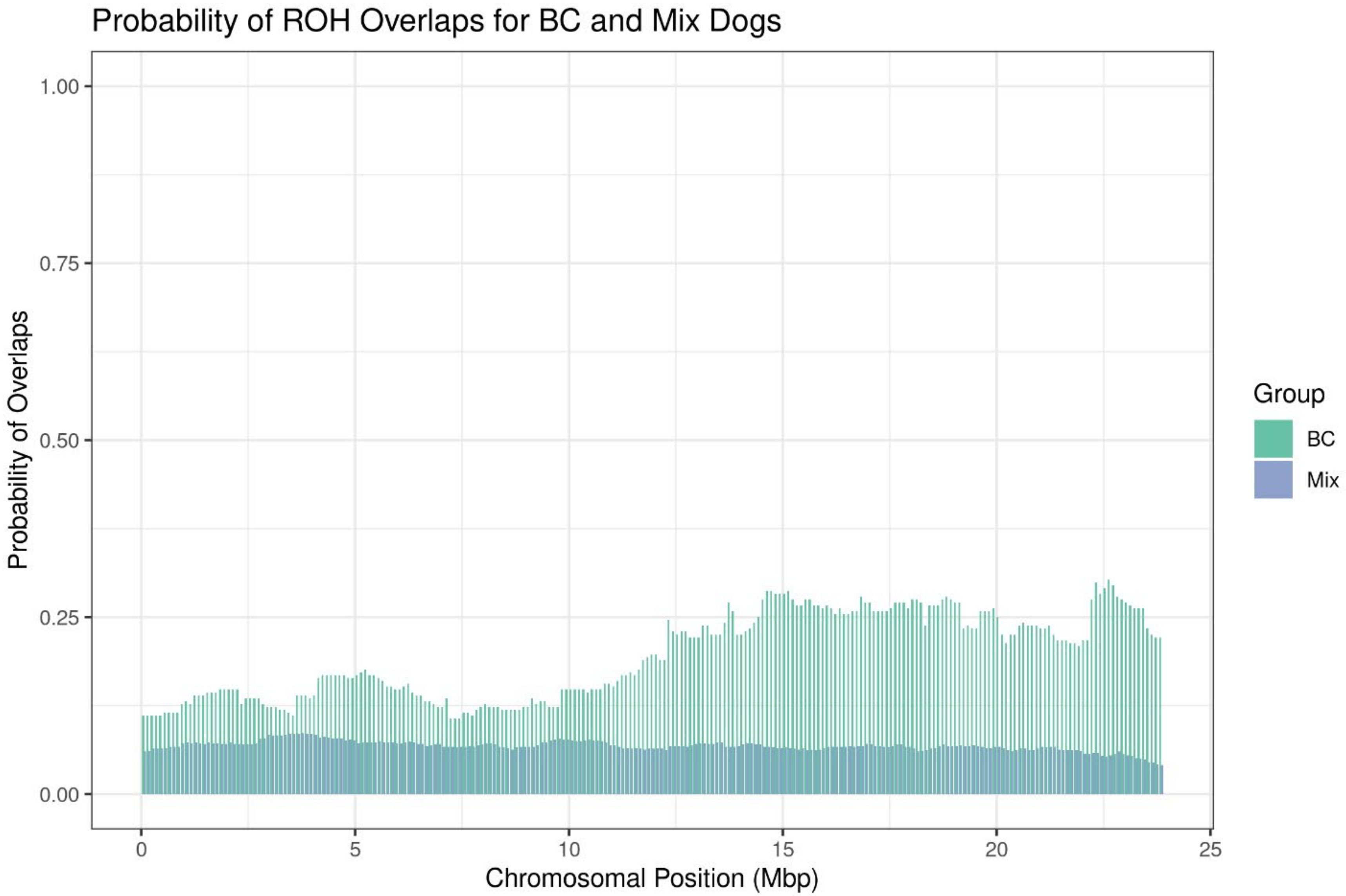
Probability of ROH Overlaps for BC and Mix Dogs
Probability of Overlaps
1.00
0.75
0.50
0.25
0.00
0
5
10
15
20
25
Chromosomal Position (Mbp)
Group
BC
Mix
