# Supplementary material for: Measures of Homozygosity and Relationship to Genetic Diversity in the Bearded Collie Breed
Source: Genes (Basel). 2025 Mar 27;16(4):378. doi: 10.3390/genes16040378 (PMC12026756; doi:10.3390/genes16040378)
Supplement: Supplementary file 1 [file genes-16-00378-s001.zip › SupplementalFileS1.pdf]

Supplemental File S1: PLINK ROH and R detectRuns Parameters.

PLINK v.1.9 command lines for ROH [1]

-- dog
--homozyg-density 50
--homozyg-gap 1000
--homozyg-kb 1000 (70 for WGS, [2])
--homozyg-snp (varied by cohort)
--homozyg-window-het 1 (0 for WGS, [2])
--homozyg-window-missing 5
--homozyg-window-snp (varied by cohort)
--homozyg-window-threshold 0.05

For SNP and WGS data, --homozyg-snp and --homozyg-window-snp were defined using a formula to calculate the average observed heterozygosity across individuals and SNPs to minimize false positives in each cohort [3–6].

$s = \frac{\ln \frac{\alpha}{N \cdot L}}{\ln(1 - \overline{H_O})}$ where N = sample size, L = number of SNPs or variants, $\overline{H}_0$ = averaged observed heterozygosity across individuals and SNPs or variants, calculated with PLINK's --het command.

The minimum number of SNPs to call ROH was 58 for Bearded Collies, 78 for purebred dogs, and 56 for mixed breed dogs. The minimum number of variants to call ROH was 51 for Bearded Collies, 262 for purebred dogs, and 138 for mixed breed dogs.

R detectRuns command lines [7]

```
> FileName_hom <- readExternalRuns(inputFile = "Plink.hom file", program = "plink")
> FileName_FROH <- Froh_inbreeding (FileName_hom,mapFile = "FileName.map", genome_wide = TRUE)
> write.csv (FileName_FROH, file = "FileName_FROH_Values.csv", row.names = FALSE,quote = FALSE)
```

References

1. Chang, C.C.; Chow, C.C.; Tellier, L.C.; Vattikuti, S.; Purcell, S.M.; Lee, J.J. Second-Generation PLINK: Rising to the Challenge of Larger and Richer Datasets. *GigaSci* **2015**, *4*, 7.
2. Dreger, D.L.; Rimbault, M.; Davis, B.W.; Bhatnagar, A.; Parker, H.G.; Ostrander, E.A. Whole Genome Sequence, SNP Chips and Pedigree Structure: Building Demographic Profiles in Domestic Dog Breeds to Optimize Genetic Trait Mapping. *Disease Models & Mechanisms* **2016**, dmm.027037.
3. Lencz, T.; Lambert, C.; DeRosse, P.; Burdick, K.E.; Morgan, T.V.; Kane, J.M.; Kucherlapati, R.; Malhotra, A.K. Runs of Homozygosity Reveal Highly Penetrant Recessive Loci in Schizophrenia. *Proc. Natl. Acad. Sci. U.S.A.* **2007**, *104*, 19942–19947.
4. Meyermans, R.; Gorssen, W.; Buys, N.; Janssens, S. How to Study Runs of Homozygosity Using PLINK? A Guide for Analyzing Medium Density SNP Data in Livestock and Pet Species. *BMC Genomics* **2020**, *21*, 94.

5. Gorssen, W.; Meyermans, R.; Janssens, S.; Buys, N. A Publicly Available Repository of ROH Islands Reveals Signatures of Selection in Different Livestock and Pet Species. *Genet Sel Evol* **2021**, *53*, 2.
6. Mastrangelo, S.; Biscarini, F.; Auzino, B.; Ragatzu, M.; Spaterna, A.; Ciampolini, R. Genome-Wide Diversity and Runs of Homozygosity in the "Braque Français, Type Pyrénées" Dog Breed. *BMC Res Notes* **2018**, *11*, 13.
7. Biscarini, F.; Cozzi, P.; Gaspa, G.; Marras, G. detectRUNS: An R Package to Detect Runs of Homozygosity and Heterozygosity in Diploid Genomes 2018.
