# Supplementary figures and images for: Measures of Homozygosity and Relationship to Genetic Diversity in the Bearded Collie Breed
Source: Genes (Basel). 2025 Mar 27;16(4):378. doi: 10.3390/genes16040378 (PMC12026756; doi:10.3390/genes16040378)

CFA 8 Probability of ROH Overlaps for BC and Mix Dogs

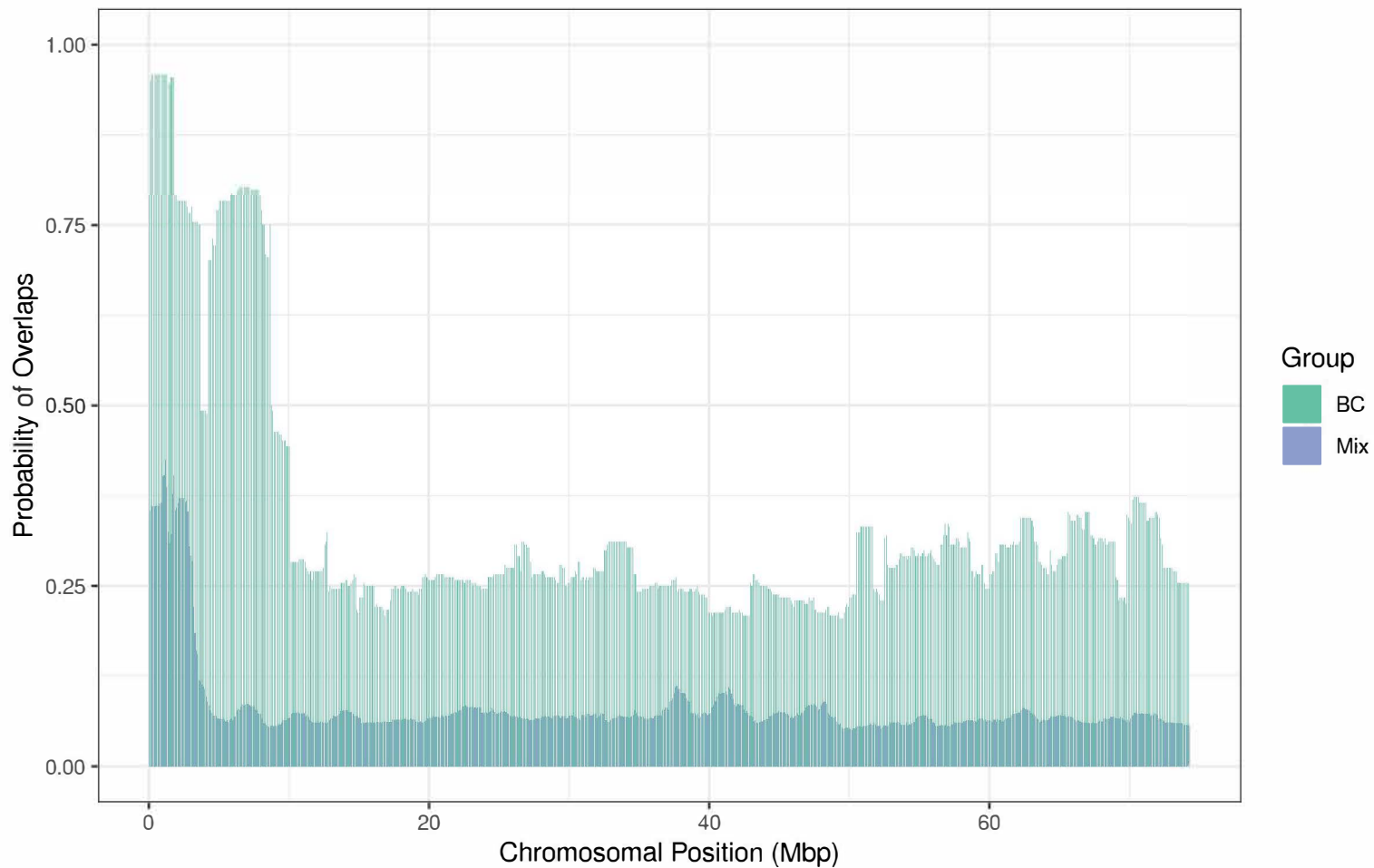

Supplement: Supplementary file 1 [file genes-16-00378-s001.zip › FigureS2.pdf]
